# Synthesis, Structure and In Vitro Cytotoxic Activity of Novel Cinchona—Chalcone Hybrids with 1,4-Disubstituted- and 1,5-Disubstituted 1,2,3-Triazole Linkers

**DOI:** 10.3390/molecules24224077

**Published:** 2019-11-11

**Authors:** Tamás Jernei, Cintia Duró, Antonio Dembo, Eszter Lajkó, Angéla Takács, László Kőhidai, Gitta Schlosser, Antal Csámpai

**Affiliations:** 1 MTA-ELTE Research Group of Peptide Chemistry, Pázmány P. sétány 1/A, H-1117 Budapest, Hungary; jernei91@gmail.com (T.J.); schlosser@caesar.elte.hu (G.S.); 2 Department of Inorganic Chemistry, Eötvös Loránd University (ELTE), Pázmány P. sétány 1/A, H-1117 Budapest, Hungary; cinti1994@gmail.com (C.D.); sapi01@yahoo.fr (A.D.); 3 Department of Genetics, Cell- and Immunobiology, Semmelweis University, Nagyvárad tér 4, H-1089 Budapest, Hungary; lajesz@gmail.com (E.L.); angela.takacs1@gmail.com (A.T.); kohlasz2@gmail.com (L.K.); 4 Department of Analytical Chemistry, Eötvös Loránd University (ELTE), Pázmány P. sétány 1/A, H-1117 Budapest, Hungary

**Keywords:** cinchona, chalcone, 1,2,3-triazole, hybrid compounds, cytotoxicity, structure-activity relationships, cell cycle analysis

## Abstract

By means of copper(I)-and ruthenium(II)-catalyzed click reactions of quinine- and quinidine-derived alkynes with azide-substituted chalcones a systematic series of novel cinchona-chalcone hybrid compounds, containing 1,4-disubstituted- and 1,5-disubstituted 1,2,3-triazole linkers, were synthesized and evaluated for their cytotoxic activity on four human malignant cell lines (PANC-1, COLO-205, A2058 and EBC-1). In most cases, the cyclization reactions were accompanied by the transition-metal-catalyzed epimerization of the C9-stereogenic centre in the cinchona fragment. The results of the in vitro assays disclosed that all the prepared hybrids exhibit marked cytotoxicity in concentrations of low micromolar range, while the C9-epimerized model comprising quinidine- and (*E*)-1-(4-(3-oxo-3-(3,4,5-trimethoxyphenyl)prop-1-en-1-yl)phenyl) fragments, connected by 1,5-disubstituted 1,2,3-triazole linker, and can be regarded as the most potent lead of which activity is probably associated with a limited conformational space allowing for the adoption of a relatively rigid well-defined conformation identified by DFT modelling. The mechanism of action of this hybrid along with that of a model with markedly decreased activity were approached by comparative cell-cycle analyses in PANC-1 cells. These studies disclosed that the hybrid of enhanced antiproliferative activity exerts significantly more extensive inhibitory effects in subG1, S and G2/M phases than does the less cytotoxic counterpart.

## 1. Introduction

It is well-documented that several alkaloids have significant anti-cancer activity, which property is widely utilized in cancer therapy (e.g., vinca alkaloids, taxol, camptothecin, etc. [1]). Although cinchona alkaloids themselves have neither cytotoxic nor cytostatic activities against human cancer cell lines [2], some of their representatives are applicable as an additive component in combined therapies to increase the efficiency of the actual anti-cancer agents expanding the scope of the treatable malignancies [3,4,5]. However, our earlier research has demonstrated that hybrid molecules comprising two cinchona fragments in complex molecular architectures of *C_2_*-symmetry display substantial cytotoxic- and cytostatic effects in the low micromolar range on a variety of human cancer cell lines [6,7]. On the other hand, chalcones—contrary to cinchona alkaloids—are highly privileged building blocks in anticancer drug agent development due to their Michael-acceptor property [8,9,10] and capability of inducing oxidative stress and uncoupling mitochondrial respiration to cancel mitochondrial membrane potential in malignant cells [11]. Thus, chalcones exert strong effects on a variety of cellular targets involved in such signalling pathways that finally lead to apoptosis. In this context, we have recently prepared and evaluated hybrids, comprising ferrocenyl-substituted chalcone and cinchona residues connected with triazole linkers, which were found to display marked cytotoxic activity against HEPG2- and HT-29 cell lines [7]. Moreover, two of their representatives were identified as pro-oxidants sensitizing three multidrug-resistant (MDR) human cancer cell lines and their sensitive counterparts (non-small cell lung carcinoma NCI-H460/R/NCI-H460, colorectal carcinoma DLD1-TxR/DLD1 and glioblastoma U87-TxR/U87) to paclitaxel [12]. As a continuation of our search for novel potent anticancer agents, prompted by the above-mentioned precedences, we envisaged the synthesis and in vitro evaluation of a further set of cinchona-chalcone hybrids with 1,2,3-triazole linkers. The results of their in vitro evaluation on human malignant cell lines (pancreatic cancer PANC-1, colon cancer COLO-205, melanoma A-2058 and non-small cell lung cancer EBC-1) supplemented with a preliminary mechanistic study are expected to provide novel structure-activity relationships that might be used as a guide for the development of further potential anticancer drugs of enhanced activity.

## 2. Results

### 2.1. Synthesis of the Targeted Hybrids

**Scheme 1:**
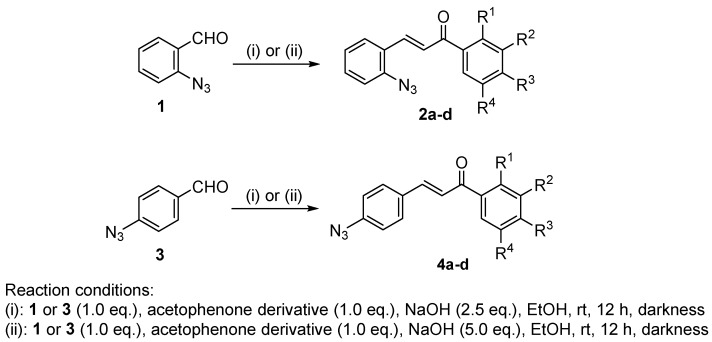
Synthesis of chalcones bearing azido-group.

**Scheme 2:**
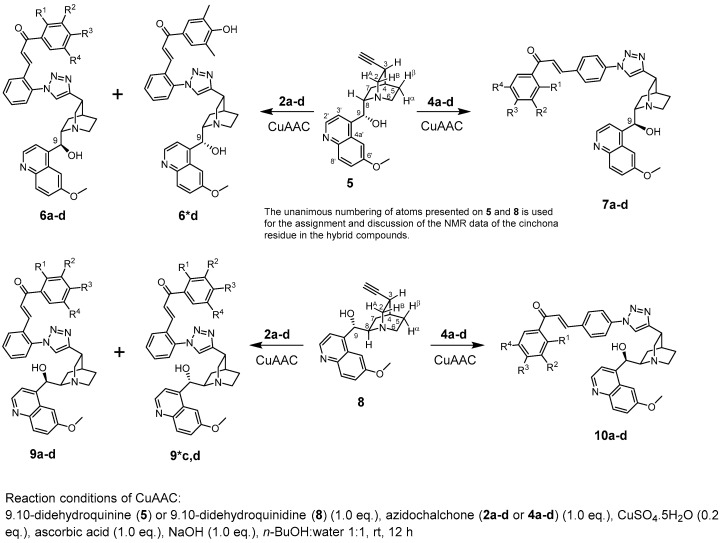
Synthesis of cinchona-chalcone hybrids with the 1,4-disubstituted 1,2,3-triazole linker.

The employed synthetic methods were adapted—in some cases with slight modifications—from our earlier publication [6], which constitutes the basis of the current work. Proceeding via a convergent synthetic pathway, by means of optimized base-catalyzed condensation (cf. Methods i and ii., Scheme 1) of substituted acetophenones with azidobenzaldehydes **1** and **3,** respectively, first we prepared azidochalcones **2a**–**d** and **4a**–**d** in moderate-to-good yields (Table 1). These intermediates were then subjected to the widely used copper(I)-mediated regioselective Sharpless [2+3] cycloaddition [13] with the readily available diastereomeric cinchona-derived alkynes **5** and **8**, respectively, generating the first group of the targeted hybrids types **6**, **7**, **9** and **10** with 1,4-disubstituted 1,2,3-triazole ring as linker (Scheme 2).

Probably due to the deactivation of the in situ formed copper(I) species partially trapped in a five-membered chelate ring coordinated by the C9-OH group and the quinuclidine N1 atom as donor sites, when lower catalyst loadings were first employed, we obtained the hybrids in significantly decreased yields. Thus, the subsequent coupling reactions of the azide and alkyne components conducted in the presence of an increased amount of CuSO_4_ (20%) allowed the isolation of the coupled products in substantially higher yields. On the other hand, the use of higher catalyst loading was expected to induce a complete inversion of the C9-stereogenic centre circumventing the laborious separation of the diastereomeric products. It is of note that the orientation of 9-hydroxyl group proved to have a very low impact on the cytostatic activity of ferrocene-containing cinchona-chalcone hybrids with closely related structures, as it was demonstrated in our previous work [7].

The formation of the epimerized products can be regarded as reactions taking place under thermodynamic control because the extra-stabilization of the epimerized products can be attributed to the intramolecular hydrogen bridge between the C9-hydroxyl group and the quinuclidine N1-atom [7,14]. In accord with the mechanism of the C9-epimerization proceeding via reversible deprotonation of the C9-H group enabled by N1-quinoline coordination to the transition-metal ion, most of the copper(I)-mediated click reactions afforded the targeted hybrids as single diastereomer with epimerized C9 stereogenic centre (**6a**–**c**, **7a**–**d**, **9a**,**b** and **10a**–**d**). However, when chalcones **2c**,**d** were used as azide components, the click reactions produced mixtures of diastereomers containing substantial amounts of isomers with unchanged C9-stereogenic centre (6*d, 9*c,**d**: Table 2). The decreased propensity of these products to undergo C9-epimerization might be the consequence of the presence of multiple donor sites in triazolyl-chalcone fragments (adjacent methoxy groups of chelating character in 9*c and phenolic hydroxyl group in 6*d and 9*d) with increased potential to trap the copper(I) species, decreasing the chance of N1-quinoline coordination, the prerequisite of C9-epimerization. It is also worth to point out that hybrids 6*d and 9*c,**d** with proximal triazole and enone fragments are also capable of trapping Cu(I) in a chelate-like complex to prevent epimerization. On the other hand, their counterparts with para-substitution pattern (7*d and 10*c,**d**), possibly formed as not isolable intermediates, seem to have a more pronounced propensity to undergo C9-epimerization, indirectly indicating a significant contribution to the above-mentioned chelate like complexation to the stereochemical stability of the C9 stereogenic centre in 6*d and 9*c,**d**.

**Scheme 3:**
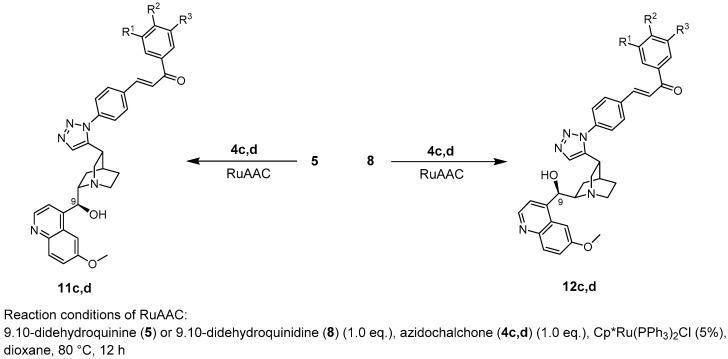
Synthesis of cinchona-chalcone hybrids with the 1,5-disubstituted 1,2,3-triazole linker.

The reactions catalyzed by complex Cp*Ru(PPh_3_)_2_Cl were also accompanied by Ru(II)-mediated C9-epimerization resulting in cinchona products **11c**,**d** and **12c**,**d** (Scheme 3) in low-to-mediocre yields (31–60%: Table 3). The facile C9-epimerization accompanying cycloadditions might again be rationalized in terms of the coordination between the metal-containing species and the quinoline N1-atom.

### 2.2. Structural Elucidation of the Novel Hybrid Compounds

**Figure 1:**
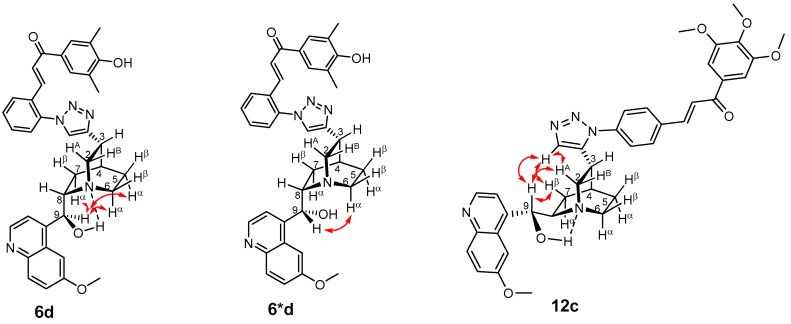
Diagnostic NOE (Nuclear Overhauser Effect) interactions detected in hybrid compounds **6d**, 6*d and **12c.**

The measured HRMS-, ^1^H- and ^13^C-NMR data of the novel hybrid compounds listed in the Materials and Method are consistent with their structure, only the following remarks about the identification of the configuration of the C9 stereogenic centre are necessary to make. Indicating its endo orientation in compound **6d**, proton H9 is involved in NOESY (Nuclear Overhauser Effect Spectroscopy) correlations with quinuclidine protons H5α-, H6 α-, and H7α, while the NOESY spectrum of 6*d with unchanged C9 stereogenic centre reveals a correlation between H9 and H6α(Figure 1). Accordingly, the approximate endo orientation of the hydroxyl group in this compound is confirmed by the signals of the proximal quinuclidine protons H5α-, H6α- and H7α downfield-shifted by 0.2–0.7 ppm relative to those of epiquinines types **6**, **7** and **11** discernible in narrow regions of their ^1^H-NMR spectra.

Again, due to the anisotropic effect of the proximal hydroxyl group, in the ^1^H-NMR spectra of quinidines 9*c and 9*d, the signals of H2A and H7β protons are downfield-shifted by ca. 0.1–0.2 ppm relative to those detected for their epiquinidine counterparts **9c** and **9d**, in which the hydroxyl group is anchored in a five-membered chelate ring by a hydrogen bond to the quinuclidine N1-atom. Accordingly, in **12c**, characteristic NOE’s were detected between proton pairs H9/H7β and H9/H2A. In keeping with their identical relative configuration, epiquinidines **9a**–**d**, **10a**–**d** and **12c**,**d** can be characterized by highly similar chemical shifts in the cinchona regions of their ^1^H- and ^13^C-NMR spectra.

### 2.3. In Vitro Cytotoxic Activity of the Novel Hybrid Compounds

The in vitro cytotoxicity of the novel hybrids expressed in IC_50_ values were determined on four selected human tumour cell cultures (PANC-1: pancreatic carcinoma of ductal origin, COLO-205: colon adenocarcinoma, A2058: malignant melanoma with high invasiveness and EBC-1: lung squamous cell carcinoma). The cells were treated with the compounds at 0.5–250 µM concentration range and the cell viability was determined by real-time impedimetry analysis (adherent cells—PANC-1) or colorimetric alamarBlue assay (cultures characterizing with weak/negligible adhesion—A2058, EBC-1, and COLO-205). We have also measured the cytotoxic activity of three azidochalcones **2c**, **2d,** and **4c** serving as representative references, which were incorporated in the most active cinchona hybrids studied in this work. The comparison of the IC_50_ values measured for the hybrids and the azide references refers to the significant contribution of the cinchona fragments to cytotoxic effect on PANC-1 and A2058 cell lines, however, these azides displayed activities against COLO-205 and EBC-1 cells similar to those exerted by the majority of the hybrids.

**Figure 2:**
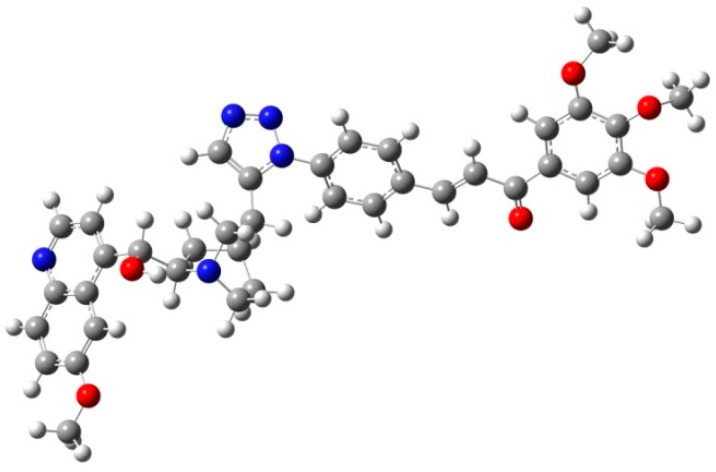
Optimized structure of hybrid **12c** generated by B3LYP/6-31+G(d) method in vacuo.

The data listed in Table 4 indicate that hybrids with 1,4-disubstituted triazole linker (types **6**, **7**, **9** and **10**) produced significant cell-line dependent cytotoxicity characterised by IC_50_ values in the low micromolar range, irrespective of the C8- and C9-configurations of the cinchona fragment. Under the conditions of the in vitro assays employed, EBC-1 proved to be the most susceptible cell line to these hybrid compounds, while PANC-1 was identified as the most resistant one in these assays. On the other hand, regarding the results obtained from the tests on four cell lines, it can be concluded that hybrids containing 3,4,5-trimethoxybenzoyl group in the chalcone moiety (**6c**, **7c**, **9c** and **10c**) proved to be the most promising leads in this group of models. It is of particular interest that, displaying the most promising cytotoxicities against all the cell lines investigated in this work, hybrids with epiquinidine skeleton and 1,5-disubstituted triazole linker (**12c** and **12d**) were found to be superior to their epiquinine counterparts **11c** and **11d**, respectively, indicating the importance of the relative configuration of the cinchona residue in the generation of the antiproliferative effect. Moreover, in keeping with the role of substitution pattern mentioned above, 3,4,5-trimethoxybenzoyl derivative **12c** was identified as the most potent model in the series of our hybrids. The outstanding activity of **12c** might partially be attributed to its relatively rigid molecular architecture, in which the full rotation of the 1,5-disubstituted triazole ring is hindered by the adjacent, thus strongly interfering chalcone- and quinuclidine units. Accordingly, the well-defined rotational position of the triazole ring can be considered as evidenced by the significant NOE’s detected between the triazole-CH and skeletal quinuclidine protons H2A, H9 and H7β. This view gains support from the optimized structure of **12c** (Figure 2) featuring the following interatomic distances from the triazole-CH: 2.780 Å (to H9); 2.449 Å (to H2A); 2.518 Å (to H7β). (This structure was obtained by DFT modelling carried out with B3LYP functional [15,16,17] using 6-31G(d) basis set [18] as implemented in the Gaussian 09 suite of programs [19]). It is likely that this skeletal structure with limited conformational space renders an enhanced potential to selectively bind to a target biomolecule involved in a signal-transferring pathway of cell-proliferation. It is of note that the 3,5-dimethyl-4-hydroxybenzoyl derivative **12d**, essentially adopting the same skeletal conformation, displays a substantially decreased activity exclusively against PANC-1 cell line pointing to an increased importance of the substituents at the chalcone terminal in the evolution of the tumour-selective cytotoxic effect induced by the cinchona-chalcone hybrids studied in this work.

Finally, it must be mentioned here that alkynes **5** and **8** were not tested on the investigated cell lines because these cinchona derivaites have not shown any cytotoxicity against HEPG-2 (human hepatocellular carcinoma) and HT-29 (human colon adenocarcinoma) cell lines as demonstareted in our earlier work [7] of which results are also in agreement with those reported by Kacprzak and coworkers [2].

**Figure 3:**
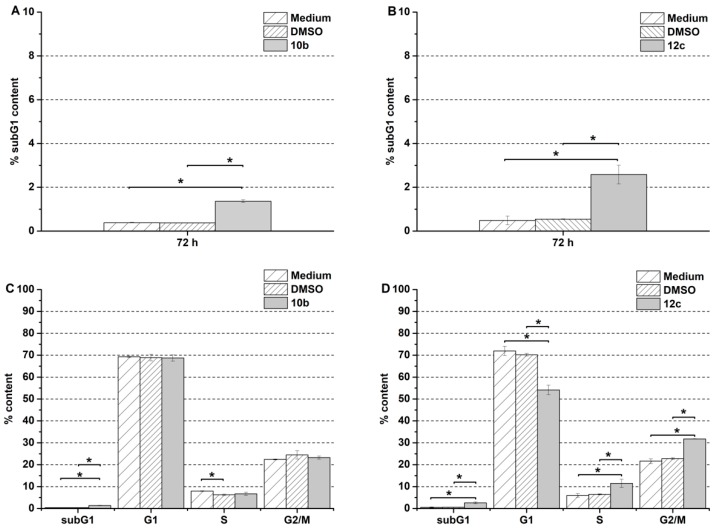
Effects of **10b** and **12c** on the cell cycle progression of PANC-1 cells. The model cell was treated with the compounds at their IC_50_ concentration for 24 h. Results are expressed as a percentage of cells in the subG1 phase ((**A**)—**10b** and (**B**)—**12c**) and the major phases (G1/G0, S and G2/M) of the cell cycle ((**C**)—**10b** and (**D)**—**12c**). Data shown in the figure represent mathematical averages of two parallel measurements.

In order to get preliminary experimental information about the mechanism of antiproliferative action of the hybrids, we undertook comparative cell-cycle analyses using the highly efficient **12c** and **10b**, a substantially less active hybrid, as suitably selected models. In PANC-1 cells **12c** reduced cell viability, as it was shown by a significant increase in the subG1 phase (representing apoptotic cells or cell fragments) (Figure 3B) and also in the percentages of S and G2/M phases (S and G2/M arrests) (Figure 3D). The other hybrid, **10b**, characterized by a weaker tumour growth inhibitory activity, caused a slight, but significant increase in the subG1 phase (Figure 3A) and, in parallel, a decrease in the S phase (Figure 3C). Of the two substances tested, the inhibitory effects of **12c** on the cell cycle were significantly more extensive (see subG1, S, G2/M arrests), which might explain its stronger antiproliferative/cytotoxic activity (lower IC_50_ value) in PANC-1 cells.

## 3. Materials and Methods

All fine chemicals were obtained from commercially available sources (Merck, Fluorochem, Molar Chemicals, VWR) and used without further purification. Dioxane was distilled from sodium benzophenone. Merck Kieselgel (230–400 mesh, 60 Å) was used for flash column chromatography. Melting points (uncorrected) were determined with a Büchi M-560. The ^1^H- and ^13^C NMR spectra of all compounds were recorded in CDCl_3_ or DMSO-*d*_6_ solution in 5 mm tubes at RT, on a Bruker DRX-500 spectrometer at 500 (^1^H) and 125 (^13^C) MHz, with the deuterium signal of the solvent as the lock and TMS as the internal standard. The HSQC, HMBC, COSY and NOESY spectra, which support the exact assignments the of ^1^H- and ^13^C NMR signals, were obtained by using the standard Bruker pulse programs. For each compound characterized in this session the numbering of atoms used for assignment of ^1^H- and ^13^C NMR signals do not correspond to IUPAC rules reflected from the given systematic names. The exact mass measurements were performed using a Q-TOF Premier mass spectrometer (Waters Corporation, 34 Maple St, Milford, MA, USA) in positive electrospray mode.

### 3.1. General Synthesis of Azidochalchones (***2a***–***c***,***4a***–***c***)

To a stirred solution of acetophenone derivative (5.0 mmol, 1.0 eq.) in EtOH (10 mL) powdered NaOH (0.50g, 12.5 mmol, 2.5 eq.) was added. After 10 min of stirring at room temperature, the solution of 2- or 4-azidobenzaldehyde (**1** or **3**) (0.74 g, 5.0 mmol, 1.0 eq.) in EtOH (2.0 mL) was added dropwise to this mixture which was then stirred for 12 h at room temperature in darkness then poured on water (20 mL). The formed precipitate was collected by filtration and washed with water.

(*E*)-3-(2-Azidophenyl)-1-(2-methoxyphenyl)prop-2-en-1-one (**2a**)

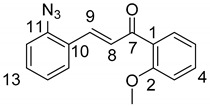

Yellow solid; Yield: 0.94 g (67%); MP.: 59.1–59.9 °C (from water); ^1^H-NMR (CDCl_3_): 7.84 (d, *J* = 16.0 Hz, 1H, H9), 7.65 (dd, *J* = 8.1 Hz, 1.4 Hz, 1H, H15), 7.61 (dd, *J* = 7.6 Hz, 1.8 Hz, 1H, H6), 7.47 (td, *J* = 7.4 Hz, 1.8 Hz, 1H, H4), 7.40 (td, *J* = 7.3 Hz, 1.5 Hz, 1H, H13), 7.37 (d, *J* = 16.0 Hz, 1H, H8), 7.19 (dd, *J* = 8.1 Hz, 0.8 Hz, 1H, H12), 7.15 (t, *J* = 7.6 Hz, 1H, H5), 7.04 (td, *J* = 7.4 Hz, 0.8 Hz, 1H, H5), 7.00 (d, *J* = 8.4 Hz, 1H, H3), 3.96 (s, 3H, OCH_3_); ^13^C-NMR (CDCl_3_): 193.0 (C7), 158.1 (C1), 139.6 (C11), 137.5 (C9), 132.9 (C4), 131.2 (C13), 130.4 (C6), 129.1 (C10), 128.6 (C8), 128.2 (C15), 126.9 (C1), 124.9 (C14), 118.9 (C12), 111.7 (C3), 55.8 (OCH_3_); HRMS exact mass calcd. for C_16_H_14_N_3_O_2_ [MH]^+^, requires *m*/*z*: 280.10805, found *m*/*z*: 280.10785.

(*E*)-3-(2-Azidophenyl)-1-(4-methoxyphenyl)prop-2-en-1-one (**2b**)

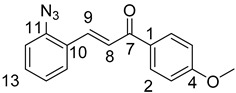

Yellow solid; Yield: 0.77 g (55%); MP.: 69.2–71.1 °C (from water); ^1^H-NMR (CDCl_3_): 8.02 (d, *J* = 8.8 Hz, 2H, H2 and H6), 7.98 (d, *J* = 15.7 Hz, 1H, H9), 7.68 (d, *J* = 7.9 Hz, 1H, H15), 7.56 (d, *J* = 15.7 Hz, 1H, H8), 7.42 (td, *J* = 7.7 Hz, 1.1 Hz, 1H, H13), 7.21 (d, *J* = 8.0 Hz, 1H, H12), 7.17 (t, *J* = 7.1 Hz, 1H, H14), 6.98 (d, *J* = 8.8 Hz, 2H, H3′ and H5), 3.88 (s, 3H, OCH_3_); ^13^C-NMR (CDCl_3_): 188.9 (C7), 163.5 (C4), 139.5 (C11), 138.4 (C9), 131.2 (C13), 131.1 (C1), 130.9 (C2 and C6), 128.6 (C15), 126.8 (C10), 124.9 (C14), 123.8 (C8), 118.9 (C12), 113.9 (C3 and C5), 55.5 (OCH_3_); HRMS exact mass calcd. for C_16_H_14_N_3_O_2_ [MH]^+^, requires *m*/*z*: 280.10805, found *m*/*z*: 280.10729.

(*E*)-3-(2-Azidophenyl)-1-(3,4,5-trimethoxyphenyl)prop-2-en-1-one (**2c**)

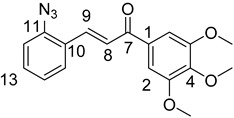

Yellow solid; Yield: 0.73 g (43%); MP.: 111.1–112.1 °C (from water); ^1^H-NMR (CDCl_3_): 7.99 (d, *J* = 15.9 Hz, 1H, H9), 7.68 (dd, *J* = 7.8 Hz, 1.1 Hz, 1H, H15), 7.46 (d, *J* = 15.9 Hz, 1H, H8), 7.43 (td, *J* = 7.7 Hz, 1.3 Hz, 1H, H13), 7.26 (s, 2H, H2 and H6), 7.22 (dd, *J* = 8.0 Hz, 0.7 Hz, 1H, H12), 7.18 (t, *J* = 7.6 Hz, 1H, H14), 3.94 (s, 6H, C3OCH_3_ and C5OCH_3_), 3.93 (s, 3H, C4OCH_3_); ^13^C-NMR (CDCl_3_): 189.6 (C7), 153.1 (C3 and C5), 142.5 (C4), 139.6 (C11), 139.1 (C9) 133.4 (C1), 131.5 (C13), 128.4 (C15), 126.6 (C10), 125.0 (C14), 123.8 (C8), 118.9 (C12), 106.2 (C2 and C6), 61.0 (C4OCH_3_), 56.4 (C3OCH_3_ and C5OCH_3_); HRMS exact mass calcd. for C_18_H_18_N_3_O_4_ [MH]^+^, requires *m*/*z*: 340.12973, found *m*/*z*: 340.12927.

(*E*)-3-(4-Azidophenyl)-1-(2-methoxyphenyl)prop-2-en-1-one (**4a**)

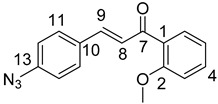

Yellow solid; Yield: 0.98 g (70%); MP.: 89.5–91.7 °C (from water); ^1^H-NMR (CDCl_3_): 7.61 (dd, *J* = 7.6 Hz, 1.8 Hz, 1H, H6), ), 7.58 (d, *J* = 15.8 Hz, 1H, H9, overlapped by H11 and H15), 7.57 (d, *J* = 8.5 Hz, 2H, H11 and H15, overlapped by H9), 7.47 (ddd, *J* = 8.4 Hz, 7.4 Hz, 1.8 Hz, 1H, H4), 7.32 (d, *J* = 15.9 Hz, 1H, H8), 7.04 (d, *J* = 8.5 Hz, 2H, H12 and H14, overlapped by H5), 7.04 (td, *J* = 7.5 Hz, 0.9 Hz, 1H, H5, overlapped by H12 and H14), 7.00 (dd, *J* = 8.4 Hz, 0.9 Hz, 1H, H3), 3.90 (s, 3H, OCH_3_); ^13^C-NMR (CDCl_3_): 192.7 (C7), 158.1 (C2), 142.0 (C9), 141.8 (C13), 132.9 (C4), 132.0 (C10), 130.7 (C6), 129.9 (C11 and C15), 129.3 (C1), 126.6 (C8), 120.8 (C5), 119.5 (C12 and C14), 111.7 (C3), 55.8 (OCH_3_); HRMS exact mass calcd. for C_16_H_14_N_3_O_2_ [MH]^+^, requires *m*/*z*: 280.10805, found *m*/*z*: 280.10690.

(*E*)-3-(4-Azidophenyl)-1-(4-methoxyphenyl)prop-2-en-1-one (**4b**)

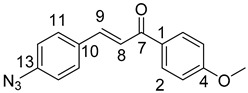

Yellow solid; Yield: 1.05 g (75%); MP.: 108.9−110.8 °C (from water); ^1^H-NMR (CDCl_3_): 8.03 (d, *J* = 8.9 Hz, 2H, H2 and H6), 7.75 (d, *J* = 15.6 Hz, 1H, H8), 7.62 (d, *J* = 8.5 Hz, 2H, H11 and H15), 7.49 (d, *J* = 15.6 Hz, 1H, H9), 7.05 (d, *J* = 8.5 Hz, 2H, H12 and H14), 6.98 (d, *J* = 8.9 Hz, 2H, H3′ and H5), 3.88 (s, 3H, OCH_3_); ^13^C-NMR (CDCl_3_): 188.5 (C7), 163.5 (C4), 142.8 (C9), 141.9 (C13), 131.9 (C10), 131.0 (C1), 130.8 (C2 and C6), 129.9 (C11 and C15), 121.3 (C8), 119.5 (C12 and C14), 113.9 (C3 and C5), 55.5 (OCH_3_); HRMS exact mass calcd. for C_16_H_14_N_3_O_2_ [MH]^+^, requires *m*/*z*: 280.10805, found *m*/*z*: 280.10788.

(*E*)-3-(4-Azidophenyl)-1-(3,4,5-trimethoxyphenyl)prop-2-en-1-one (**4c**)

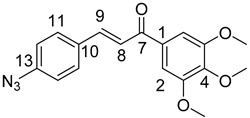

Yellow solid; Yield: 1.28 g (75%); MP.: 136.2-138.0 °C (from water); ^1^H-NMR (CDCl_3_): 7.80 (d, *J* = 15.6 Hz, 1H, H9), 7.67 (d, *J* = 8.5 Hz, 2H, H11 and H15), 7.45 (d, *J* = 15.6 Hz, 1H, H8), 7.29 (s, 2H, H2 and H6), 7.10 (d, *J* = 8.5 Hz, 2H, H12 and H14), 3.97 (s, 6H, C3OCH_3_ and C5OCH_3_), 3.96 (s, 3H, C4CH_3_); ^13^C-NMR (CDCl_3_): 189.0 (C7), 153.2 (C3 and C5), 143.6 (C9) 142.2 (C4 and C13), 133.5 (C1), 131.7 (C10), 130.0 (C11 and C15), 121.1 (C8), 119.6 (C12 and C14), 106.1 (C2 and C6), 61.0 (C4OCH_3_), 56.4 (C3OCH_3_ and C5OCH_3_) HRMS exact mass calcd. for C_18_H_18_N_3_O_4_ [MH]^+^, requires *m*/*z*: 340.12973, found *m*/*z*: 340.12892.

### 3.2. General Synthesis of Phenolic Hydroxyl Group-Containing Azidochalchones (***2d***, ***4d***)

To a stirred solution of the acetophenone component (5.0 mmol, 1.0 eq.) in EtOH (10 mL) powdered NaOH (1.00g, 25.0 mmol, 5.0 eq.) was added. After 10 min of stirring at room temperature, the solution of 2- or 4-azidobenzaldehyde (**1** or **3**) (0.74 g, 5.0 mmol, 1.0 eq.) in EtOH (2.0 mL) was added dropwise to this mixture which was then stirred for 12 h at room temperature in darkness, poured on water (20 mL) and acidified with 2 N hydrochloric acid to pH 3. The formed precipitate was collected by filtration and washed with water.

(*E*)-3-(2-Azidophenyl)-1-(3,5-dimethyl-4-hydroxyphenyl)prop-2-en-1-one (**2d**)

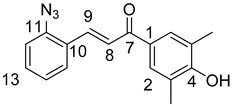

Yellow solid; Yield: 0.37 g (25%); MP.: 134.6–136.7 °C (from water); ^1^H-NMR (DMSO-*d*_6_): 9.22 (s, 1H, OH), 8.06 (dd, *J* = 8.0 Hz, 1.4 Hz, 1H, H15), 7.87 (d, *J* = 15.6 Hz, 1H, H8), 7.81 (d, *J* = 15.6 Hz, 1H, H9), 7.77 (s, 2H, H2 and H6), 7.47 (ddd, *J* = 8.4 Hz, 7.3 Hz, 1.5 Hz, 1H, H13), 7.33 (dd, *J* = 8.1 Hz, 1.1 Hz, 1H, H12), 7.21 (t, *J* = 7.6 Hz, 1H, H14), 2.20 (s, 2H, CH_3_); ^13^C-NMR (DMSO-*d*_6_): 187.6 (C7), 124.7 (C3 and C5), 158.9 (C4), 139.3 (C11), 136.3 (C9) 129.5 (C1), 132.2 (C13), 128.5 (C15), 126.4 (C10), 125.5 (C14), 123.8 (C8), 119.8 (C12), 130.1 (C2 and C6), 17.1 (CH_3_); HRMS exact mass calcd. for C_17_H_16_N_3_O_2_ [MH]^+^, requires *m*/*z*: 294.12370, found *m*/*z*: 294.12301.

(*E*)-3-(4-Azidophenyl)-1-(3,5-dimethyl-4-hydroxyphenyl)prop-2-en-1-one (**4d**)

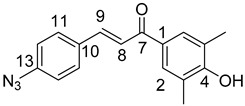

Yellow solid; Yield: 0.37 g (25%); MP.: 146.8–149.2 °C (from water); ^1^H-NMR (CDCl_3_): 7.72 (d, *J* = 15.6 Hz, 1H, H9), 7.70 (s, 2H, H2 and H6), 7.61 (d, *J* = 8.3 Hz, 2H, H11 and H15), 7.46 (d, *J* = 15.6 Hz, 1H, H8), 7.04 (d, *J* = 8.3 Hz, 2H, H12 and H14), 5.19 (br s, 3H, OH), 2.30 (s, 6H, CH_3_); ^13^C-NMR (CDCl_3_): 188.9 (C7), 123.1 (C3 and C5), 156.7 (C4), 129.9 (C11 and C15), 142.6 (C9), 130.6 (C1), 141.9 (C13), 132.0 (C10), 119.5 (C12 and C14), 121.5 (C8), 129.8 (C2 and C6), 15.9 (CH_3_); HRMS exact mass calcd. for C_17_H_16_N_3_O_2_ [MH]^+^, requires *m*/*z*: 294.12370, found *m*/*z*: 294.12326.

### 3.3. General Synthesis of Cinchona-Chalcone Hybrids with 1,4-Disubstituted Triazole Linkers

10,11-Didehydroquinine (**5**) or 10,11-didehydroquinidine (**8**) (322 mg, 1.0 mmol, 1.0 eq.), azidochalchone (**2a**–**d, 4a**–**d**) (1.0 mmol, 1.0 eq.), CuSO_4_·5H_2_O (50 mg, 0.2 mmol, 0.2 eq.), NaOH (40 mg, 1.0 mmol, 1.0 eq.) and L-ascorbic acid (176 mg, 1.0 mmol, 1.0 eq.) were suspended in a mixture of water (1 mL) and n-butanol (1 mL). The resulting mixture was stirred at room temperature for 12 h, diluted with water (15 mL), and extracted with DCM (5 × 25 mL). The combined organic phase was washed with brine, dried on Na_2_SO_4_, and evaporated to dryness. The residue was purified by flash chromatography on silica gel, using DCM:MeOH mixtures (the ratio of DCM and MeOH was varied between 60:1 and 10:1). The analytical sample was sequentially crystallized by EtOH-water and Et_2_O.

### 3.4. General Synthesis of Cinchona-Chalcone Hybrids with 1,5-Disubstituted Triazole Linkers

10,11-Didehydroquinine (**5**) or 10,11-didehydroquinidine (**8**) (322 mg, 1.0 mmol, 1.0 eq.), azidochalchone (**2c**,**d**, **4c**,**d**) (1.0 mmol, 1.0 eq.) and pentamethylcyclopentadienylbis(triphenylphosphine)ruthenium(II) chloride (40 mg, 0,05 mmol, 5%) were dissolved in dioxane (4 mL). The resulting mixture was stirred at 80 °C for 12 h diluted with water (15 mL) and extracted with DCM (5 × 25 mL). The combined organic phase was washed with brine, dried on Na_2_SO_4_, and evaporated to dryness. The residue was purified by flash chromatography on silica gel, using DCM:MeOH mixtures (the ratio of DCM and MeOH was varied between 60:1 and 10:1). The analytical sample was sequentially crystallized by EtOH-water and Et_2_O.

Spectral characterization (^1^H- and ^13^C-NMR data and HRMS) of the novel compounds can be found in the Supplementary Materials.

(*E*)-3-{2-{4-{(1*S*,3*R*,4*S*,6*S*)-6-[(*S*)-Hydroxy(6-methoxyquinolin-4-yl)methyl]quinuclidin-3-yl}-1*H*-1,2,3-triazol-1-yl}phenyl}-1-(2-methoxyphenyl)prop-2-en-1-one (**6a**)

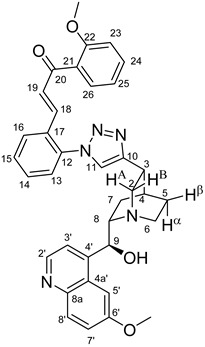

Pale yellow solid; Yield: 578 mg (91%); MP.: ~121 °C (dec., from diethyl ether); ^1^H-NMR (DMSO-*d*_6_ at 45 °C): 8.68 (d, *J* = 4.5 Hz, 1H, H2′), 8.26 (s, 1H, H11), 7.98 (dd, *J* = 7.5 Hz, 1.5 Hz, 1H, H16), 7.90 (d, *J* = 9.2 Hz, 1H, H8′), 7.66 (d, *J* = 2.3 Hz, 1H, H5′), 7.55–7.59 (m, 3H, H3′ and H14 and H15), 7.41 (td, *J* = 8.4 Hz, 1.7 Hz, 1H, H24), 7.38 (dd, *J* = 7.5 Hz, 1.3 Hz, 1H, H13), 7.36 (dd, *J* = 9.2 Hz, 2.7 Hz, 1H, H7′), 7.29 (dd, *J* = 7.5 Hz, 1.7 Hz, 1H, H26), 7.17 (d, *J* = 15.9 Hz, 1H, H19), 6.98 (d, *J* = 8.4 Hz, 1H, H23), 1H, H25), 6.92 (td, *J* = 7.5 Hz, 0.8 Hz, 6.88 (d, *J* = 15.9 Hz, 1H, H18), 6.10 (br s, 1H, C9OH), 5.96 (br m, 1H, H9), 3.97 (s, 3H, C6′OCH_3_), 3.70 (s, 3H, C22OCH_3_), 3.62 (br m, H8), 3.57 (br m, 1H, H2A), 3.39 (br m, 1H, H2B), 3.38 (br m, 1H, H3), 3.82 (br m, 1H, H6α), 3.06 (br m, 1H, H6β), 2.16 (br m, 1H, H4), 2.01 (br m, 1H, H5α), 1.86 (br m, 1H, H5β), 1.94 (br m, 1H, H7α), 1.39 (br m, 1H, H7β); ^13^C-NMR (DMSO-*d*_6_ at 45 °C): 192.7 (C20), 158.2 (C6′), 158.1 (C22), 147.0 (C4′), 148.9 (C10), 147.9 (C2′), 144.5 (C8a′), 137.2 (C18), 136.7 (C12), 133.5 (C21), 133.4 (C24), 131.8 (C8′), 131.4 (C14), 130.8 (C17), 130.7 (C15), 130.3 (C19), 128.9 (C16), 127.7 (C26), 127.1 (C13), 126.7 (C4a′), 125.1 (C11), 121.9 (C7′), 120.9 (C25), 119.6 (C3′), 112.8 (C23), 102.9 (C5′), 68.0 (C9), 60.3 (C8), 57.0 (C6′OCH_3_), 56.5 (C22OCH_3_), 54.5 (C2), 43.2 (C6), 31.9 (C3) 27.9 (C4), 25.3 (C5), 25.2 (C7); HRMS exact mass calcd. for C_36_H_36_N_5_O_4_ [MH]^+^, requires *m*/*z*: 602.27618, found *m*/*z*: 602.27545.

(*E*)-3-{2-{4-{(1*S*,3*R*,4*S*,6*S*)-6-[(*S*)-Hydroxy(6-methoxyquinolin-4-yl)methyl]quinuclidin-3-yl}-1*H*-1,2,3-triazol-1-yl}phenyl}-1-(4-trimethoxyphenyl)prop-2-en-1-one (**6b**)

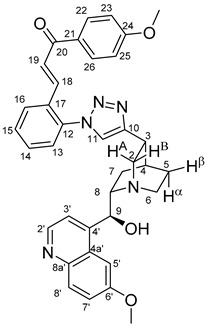

Pale yellow solid; Yield: 433 mg (72%); MP.: ~151 °C (dec., from diethyl ether); ^1^H-NMR (DMSO-*d*_6_ at 45 °C): 8.62 (d, *J* = 4.5 Hz, 1H, H2′), 8.29 (s, 1H, H11), 8.20 (dd, *J* = 7.6 Hz, 1.5 Hz, 1H, H16), 7.99 (d, *J* = 8.9 Hz, 2H, H22 and H26), 7.88 (d, *J* = 9.1 Hz, 1H, H8′), 7.68 (d, *J* = 15.6 Hz, 1H, H19), 7.61 (td, *J* = 7.4 Hz, 1.3 Hz, 1H, H15), 7.59 (td, *J* = 7.5 Hz, 1.6 Hz, 1H, H14), 7.56 (d, *J* = 2.6 Hz, 1H, H5′), 7.49 (d, *J* = 4.5 Hz, 1H, H3′), 7.45 (dd, *J* = 7.5 Hz, 1.5 Hz, 1H, H13), 7.33 (dd, *J* = 9.2 Hz, 2.7 Hz, 1H, H7′), 7.21 (d, *J* = 15.6 Hz, 1H, H18), 7.01 (d, *J* = 8.9 Hz, 2H, H23 and H25), 5.59 (br m, 1H, C9OH), 5.41 (br s, 1H, C9), 3.89 (s, 3H, C6′OCH_3_), 3.83 (s, 3H, C24OCH_3_), 3.42 (br m, 1H, H8), 3.36 (br m, 1H, H2A), 3.24 (br m, 2H, H6α and H6β), 3.14 (br m, 1H, H3), 2.70 (br m, 1H, H2B), 2.14 (br m, 1H, H4), 1.86 (br m, 1H, H5α), 1.72 (br m, 1H, H7α), 1.68 (br m, 1H, H5β), 1.63 (br m, 1H, H7β); ^13^C-NMR (DMSO-*d*_6_ at 45 °C): 187.8 (C20), 163.9 (C24), 157.6 (C6′), 150.7 (C10), 148.6 (C4′), 147.9 (C2′), 144.5 (C8a′), 137.3 (C18), 137.0 (C12), 131.7 (C8′), 131.4 (C14 and C22 and C26), 131.1 (C17), 130.7 (C21), 130.5 (C15), 128.7 (C16), 127.4 (C4a′), 127.0 (C13), 125.8 (C19), 124.9 (C11), 121.4 (C7′), 119.7 (C3′), 114.6 (C23 and C25), 103.3 (C5′), 70.7 (C9), 60.8 (C8), 56.2 (C6′OCH_3_), 56.1 (C24OCH_3_), 55.7 (C2), 42.7 (C6), 33.0 (C3), 28.1 (C4), 27.1 (C7 and C5); HRMS exact mass calcd. for C_36_H_36_N_5_O_4_ [MH]^+^, requires *m*/*z*: 602.27618, found *m*/*z*: 602.27608.

(*E*)-3-{2-{4-{(1*S*,3*R*,4*S*,6*S*)-6-[(*S*)-Hydroxy(6-methoxyquinolin-4-yl)methyl]quinuclidin-3-yl}-1*H*-1,2,3-triazol-1-yl}phenyl}-1-(3,4,5-trimethoxyphenyl)prop-2-en-1-one (**6c**)

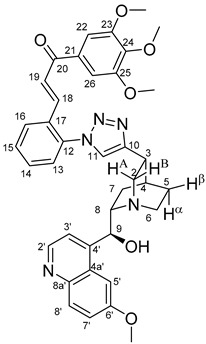

Yellow solid; Yield: 503 mg (74%); MP.: ~157 °C (dec., from diethyl ether); ^1^H-NMR (DMSO-*d*_6_ at 45 °C): 8.62 (d, *J* = 4.5 Hz, 1H, H2′), 8.30 (s, 1H, H11), 8.22 (dd, *J* = 7.6 Hz, 1.4 Hz, 1H, H16), 7.88 (d, *J* = 9.2 Hz, 1H, H8′), 7.71 (d, *J* = 15.6 Hz, 1H, H19), 7.62 (td, *J* = 7.3 Hz, 1.1 Hz, 1H, H15, overlapped by H5′ and H14), 7.59 (td, *J* = 7.6 Hz, 1.3 Hz, 1H, H14, overlapped by H5′ and H15), 7.59 (1H, H5′, overlapped by H14 and H15), 7.50 (d, *J* = 4.3 Hz, 1H, H3′), 7.44 (d, *J* = 7.4 Hz, 1H, H13), 7.33 (dd, *J* = 9.2 Hz, 2.7 Hz, 1H, H7′), 7.28 (s, 2H, H22 and H26), 7.23 (d, *J* = 15.6 Hz, 1H, H18), 5.78 (br m, 1H, C9OH, overlapped by H9), 5.67 (br s, 1H, H9, overlapped by C9OH), 3.92 (s, 3H, C6′OCH_3_), 3.84 (s, 6H, C23OCH_3_ and C25OCH_3_), 3.76 (s, 3H, C24OCH_3_), 3.57 (br m, 1H, H6α), 3.50 (br m, 1H, H8), 3.30-3.41 (br m, 2H, H2A and H2B), 3.28 (br m, 1H, H3), 2.86 (br m, 1H, H6β), 2.16 (br m, 1H, H4), 1.91 (br m, 1H, H5α), 1.86 (br m, 1H, H7α), 1.77 (br m, 1H, H5β), 1.57 (br m, 1H, H7β); ^13^C-NMR (DMSO-*d*_6_ at 45 °C): 188.3 (C20), 157.7 (C6′), 153.5 (C23 and C25), 153.1 (C10), 150.2 (C4′), 147.9 (C2′), 144.5 (C8a′), 143.3 (C24), 137.8 (C18), 137.0 (C12), 133.1 (C21), 131.7 (C8′), 131.5 (C14), 130.9 (C17), 130.5 (C15), 128.8 (C16), 127.1 (C4a′), 127.0 (C13), 125.7 (C19), 124.9 (C11), 121.5 (C7′), 119.6 (C3′), 107.3 (C22 and C26), 103.2 (C5′), 70.3 (C9), 60.7 (C24OCH_3_), 60.6 (C8), 56.9 (C23OCH_3_ and C25OCH_3_), 56.4 (C6′OCH_3_), 55.1 (C2), 42.9 (C6), 32.5 (C3), 28.3 (C4), 26.5 (C5), 22.4 (C7); HRMS exact mass calcd. for C_38_H_40_N_5_O_6_ [MH]^+^, requires *m*/*z*: 662.29731, found *m*/*z*: 662.29623.

(*E*)-3-{2-{4-{(1*S*,3*R*,4*S*,6*S*)-6-[(*S*)-Hydroxy(6-methoxyquinolin-4-yl)methyl]quinuclidin-3-yl}-1*H*-1,2,3-triazol-1-yl}phenyl}-1-(4-hydroxy-3,5-dimethylphenyl)prop-2-en-1-one (**6d**)

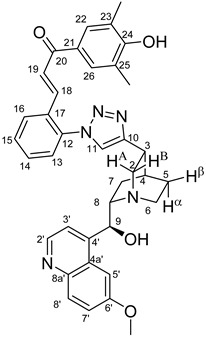

Orange solid; Yield: 184 mg (30%); MP.: ~179 °C (dec., from diethyl ether); ^1^H-NMR (DMSO-*d*_6_ at 45 °C): 9.01 (br s, 1H, C24OH), 8.63 (d, *J* = 4.5 Hz, 1H, H2′), 8.27 (s, 1H, H11), 8.18 (dd, *J* = 7.7 Hz, 1.5 Hz, 1H, H16), 7.89 (d, *J* = 9.2 Hz, 1H, H8′), 7.65 (s, 2H, H22 and H26, overlapped by H19), 7.65 (d, *J* = 15.6 Hz, 1H, H19, overlapped by H22 and H26), 7.60 (td, *J* = 7.5 Hz, 1.2 Hz, 1H, H15), 7.56–7.58 (m, 2H, H5′ and H14), 7.50 (d, *J* = 4.5 Hz, 1H, H3′), 7.42 (dd, *J* = 7.6 Hz, 1.2 Hz, 1H, H13), 7.34 (dd, *J* = 9.2 Hz, 2.7 Hz, 1H, H7′), 7.18 (d, *J* = 15.6 Hz, 1H, H18), 5.69 (br m, 1H, C9OH), 5.52 (br s, 1H, C9), 3.90 (s, 3H, C6′OCH_3_), 3.47 (br m, 1H, H8), 3.46 (br m, 1H, H6α) 3.27–3.35 (br m, 2H, H2A and H2B), 3.22 (br m, 1H, H3), 2.79 (br m, 1H, H6β), 2.21 (s, 6H, CH_3_), 2.16 (br m, 1H, H4), 1.87 (br m, 1H, H5α), 1.80 (br m, 1H, H7α), 1.72 (br m, 1H, H5β), 1.61 (br m, 1H, H7β); ^13^C-NMR (DMSO-*d*_6_ at 45 °C): 187.8 (C20), 158.9 (C24), 157.7 (C6′), 150.3 (C10), 148.3 (C4′), 147.9 (C2′), 144.5 (C8a′), 136.8 (C12), 136.5 (C18), 131.7 (C8′), 131.2 (C14 and C17), 130.5 (C15), 130.0 (C22 and C26), 129.3 (C21), 128.6 (C16), 127.2 (C4a′), 127.0 (C13), 126.1 (C19), 124.9 (C11), 124.8 (C23 and C25), 121.4 (C7′), 119.6 (C3′), 103.3 (C5′), 70.1 (C9) 60.6 (10), 56.4 (C6′OCH_3_), 55.3 (C2), 42.8 (C6), 32.7 (C3), 28.3 (C4), 26.8 (C5), 22.8 (C7), 16.8 (CH_3_); HRMS exact mass calcd. for C_37_H_38_N_5_O_4_ [MH]^+^, requires *m*/*z*: 616.29183, found *m*/*z*: 616.29167.

(*E*)-3-{2-{4-{(1*S*,3*R*,4*S*,6*S*)-6-[(*R*)-Hydroxy(6-methoxyquinolin-4-yl)methyl]quinuclidin-3-yl}-1*H*-1,2,3-triazol-1-yl}phenyl}-1-(4-hydroxy-3,5-dimethylphenyl)prop-2-en-1-one (6*d)

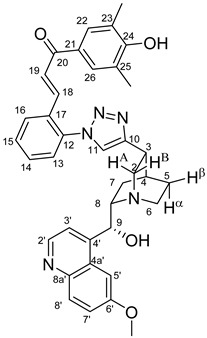

Yellow solid; Yield: 308 mg (50%); MP.: ~171 °C (dec., from diethyl ether); ^1^H-NMR (DMSO-*d*_6_ at 45 °C): 9.03 (br s, 1H, C24OH), 8.66 (d, *J* = 4.5 Hz, 1H, H2′), 8.34 (s, 1H, H11), 8.15 (dd, *J* = 7.7 Hz, 1.5 Hz, 1H, H16), 7.89 (d, *J* = 9.2 Hz, 1H, H8′), 7.70 (d, *J* = 2.6 Hz, 1H, H5′), 7.63 (s, 2H, H22 and H26, overlapped by H19), 7.63 (d, *J* = 15.6 Hz, 1H, H19, overlapped by H22 and H26), 7.58 (td, *J* = 7.6 Hz, 1.1 Hz, 1H, H15, overlapped by H3′), 7.52 (td, *J* = 7.7 Hz, 1.3 Hz, 1H, H14), 7.50 (d, *J* = 4.5 Hz, 1H, H3′, overlapped by H15), 7.34–7.36 (2H, H7′ and H13), 7.07 (d, *J* = 15.6 Hz, 1H, H18), 6.34 (br m, 1H, C9OH), 6.26 (br s, 1H, C9), 4.06 (br m, 1H, H6α), 4.02 (s, 3H, C6′OCH_3_), 3.61–3.76 (br m, 4H, H8 and H2A and H2B and H3), 3.28 (br m, 1H, H6β), 2.30 (br m, 1H, H4), 2.22 (s, 6H, CH_3_), 2.09–2.12 (br m, 2H, H7α and H5α), 2.00 (t, *J* = 11.1 Hz, 1H, H5β), 1.37 (br m, 1H, H7β); ^13^C-NMR (DMSO-*d*_6_ at 45 °C): 187.7 (C20), 158.9 (C24), 158.5 (C6′), 148.1 (C10), 147.8 (C2′), 145.8 (C4′), 144.4 (C8a′), 136.6 (C12), 136.2 (C18), 131.8 (C8′), 131.2 (C17), 131.1 (C14), 130.6 (C15), 130.0 (C22 and C26), 129.2 (C21), 128.6 (C16), 127.0 (C13), 126.3 (C19), 126.2 (C4a′), 125.5 (C11), 124.8 (C23 and C25), 122.2 (C7′), 119.6 (C3′), 102.7 (C5′), 66.5 (C9) 60.1 (C8), 57.4 (C6′OCH_3_), 53.7 (C2), 43.5 (C6), 31.3 (C3), 27.7 (C4), 24.3 (C5), 18.7 (C7), 16.9 (CH_3_); HRMS exact mass calcd. for C_37_H_38_N_5_O_4_ [MH]^+^, requires *m*/*z*: 616.29183, found *m*/*z*: 616.29091.

(*E*)-3-{4-{4-{(1*S*,3*R*,4*S*,6*S*)-6-[(*S*)-Hydroxy(6-methoxyquinolin-4-yl)methyl]quinuclidin-3-yl}-1*H*-1,2,3-triazol-1-yl}phenyl}-1-(2-methoxyphenyl)prop-2-en-1-one (**7a**)

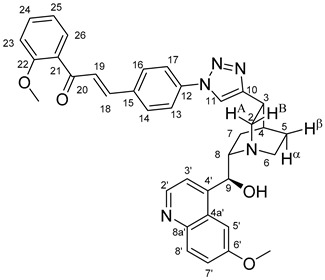

Pale yellow solid; Yield: 337mg (56%); MP.: ~170 °C (dec., from diethyl ether); ^1^H-NMR (DMSO-*d*_6_ at 45 °C): 8.64 (d, *J* = 4.4 Hz, 1H, H2′), 8.62 (s, 1H, H11), 7.89 (d, *J* = 9.2 Hz, 1H, H8′), 7.83–7.85 (m, 4H, H13 and H14 and H16 and H17), 7.59 (d, *J* = 2.6 Hz, 1H, H5′), 7.46–7.53 (m, 4H, H3′ and H18 and H24 and H26), 7.39 (d, *J* = 16.0 Hz, 1H, H19), 7.36 (dd, *J* = 9.2 Hz, 2.6 Hz, 1H, H7′), 7.15 (d, *J* = 8.3 Hz, 1H, H23), 7.03 (td, *J* = 7.5 Hz, 0.8 Hz, 1H, H25), 5.83-5.71 (br s, 2H, C9OH and H9) 3.94 (s, 3H, C6′OCH_3_), 3.83 (s, 3H, C22OCH_3_), 3.45–3.48 (br m, 4H, H8 and H2A and H2B and H6α), 3.28 (br m, 1H, H3), 2.89 (br m, 1H, H6β), 2.22 (br m, 1H, H4), 1.92 (br m, 1H, H5α), 1.85 (br m, 1H, H7α), 1.78 (br m, 1H, H5β), 1.51 (br m, 1H, H7β); ^13^C-NMR (DMSO-*d*_6_ at 45 °C): 192.5 (C20), 158.4 (C22), 157.9 (C6′), 150.1 (C10), 147.9 (C2′), 145.8 (C4′), 144.5 (C8a′), 141.4 (C18), 138.1 (C12), 135.2 (C15), 133.5 (C24), 131.7 (C8′), 130.3 (C14 and C16), 129.9 (C26), 129.4 (C21), 128.5 (C19), 127.0 (C4a′), 121.7 (C7′), 121.1 (C25), 120.8 (C11), 120.6 (C13 and C17), 119.6 (C3′), 113.1 (C23), 103.1 (C5′), 68.2 (C9), 56.6 (C6′OCH_3_), 56.5 (C22OCH_3_), 60.4 (C8), 54.9 (C2), 43.0 (C6), 32.5 (C3), 27.5 (C4), 27.0 (C7 and C5); HRMS exact mass calcd. for C_36_H_37_N_5_O_4_ [MH]^+^, requires *m*/*z*: 602.27618, found *m*/*z*: 602.27570.

(*E*)-3-{4-{4-{(1*S*,3*R*,4*S*,6*S*)-6-[(*S*)-Hydroxy(6-methoxyquinolin-4-yl)methyl]quinuclidin-3-yl}-1*H*-1,2,3-triazol-1-yl}phenyl}-1-(4methoxyphenyl)prop-2-en-1-one (**7b**)

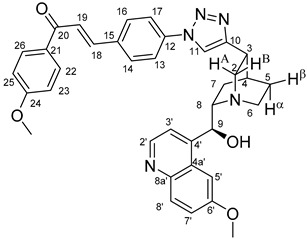

Pale yellow solid; Yield: 475 mg (79%); MP.: ~154 °C (dec., from diethyl ether); ^1^H-NMR (DMSO-*d*_6_ at 45 °C): 8.64 (d, *J* = 4.5 Hz, 1H, H2′), 8.62 (s, 1H, H11), 8.11 (d, *J* = 8.9 Hz, 2H, H22 and H26), 7.99 (d, *J* = 8.7 Hz, 2H, H16 and H14), 7.88–7.90 (m, 4H, H8′ and H13 and H17 and H19), 7.68 (d, *J* = 15.5 Hz, 1H, H18), 7.57 (d, *J* = 2.6 Hz, 1H, H5′), 7.49 (d, *J* = 4.5 Hz, 1H, H3′), 7.35 (dd, *J* = 9.2 Hz, 2.7 Hz, 1H, H7′), 7.05 (d, *J* = 8.9 Hz, 2H, H23 and H25), 5.67 (br m, 1H, C9OH), 5.50 (br s, 1H, C9), 3.91 (s, 3H, C6′OCH_3_), 3.84 (s, 3H, C24OCH_3_), 3.45 (br m, 1H, H6α), 3.39 (br m, 1H, H8), 3.27 (br m, 2H, H2A and H2B), 3.15 (br m, 1H, H3), 2.75 (br m, 1H, H6β), 2.19 (br m, 1H, H4), 1.86 (br m, 1H, H5α), 1.76 (br m, 1H, H7α), 1.70 (br m, 1H, H5β), 1.58 (br m, 1H, H7β); ^13^C-NMR (DMSO-*d*_6_ at 45 °C): 188.0 (C20), 163.9 (C24), 157.7 (C6′), 151.3 (C10), 148.4 (C4′), 147.9 (C2′), 144.5 (C8a′), 142.0 (C18), 138.2 (C12), 135.4 (C15), 131.7 (C8′), 131.4 (C23 and C25), 131.0 (C21), 130.5 (C14 and C16), 127.2 (C4a′), 123.8 (C19), 121.5 (C7′), 120.7 (C11), 120.5 (C13 and C17), 119.6 (C3′), 114.6 (C22 and C26), 103.3 (C5′), 70.2 (C9), 60.6 (C8), 56.4 (C6′OCH_3_), 56.1 (C24OCH_3_), 55.3 (C2), 42.7 (C6), 32.9 (C3), 27.7 (C4), 27.0 (C7 and C5); HRMS exact mass calcd. for C_36_H_36_N_5_O_4_ [MH]^+^, requires *m*/*z*: 602.27618, found *m*/*z*: 602.27553.

(*E*)-3-{4-{4-{(1*S*,3*R*,4*S*,6*S*)-6-[(*S*)-Hydroxy(6-methoxyquinolin-4-yl)methyl]quinuclidin-3-yl}-1*H*-1,2,3-triazol-1-yl}phenyl}-1-(3,4,5-trimethoxyphenyl)prop-2-en-1-one (**7c**)

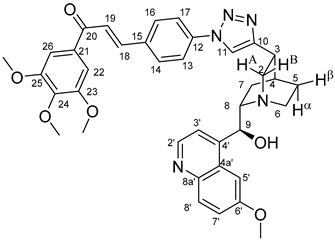

Yellow solid; Yield: 404 mg (61%); MP.: ~150 °C (dec., from diethyl ether); ^1^H-NMR (DMSO-*d*_6_ at 45 °C): 8.64 (d, *J* = 4.5 Hz, 1H, H2′), 8.63 (s, 1H, H11), 8.02 (d, *J* = 8.6 Hz, 2H, H16 and H14), 7.87–7.90 (m, 4H, H8′ and H13 and H17 and H19), 7.72 (d, *J* = 15.5 Hz, 1H, H18), 7.57 (d, *J* = 2.5 Hz, 1H, H5′), 7.50 (d, *J* = 4.5 Hz, 1H, H3′), 7.39 (s, 2H, H22 and H26), 7.35 (dd, *J* = 9.2 Hz, 2.7 Hz, 1H, H7′), 5.77–5.44 (br s, 2H, C9OH, H9), 3.92 (s, 3H, C6′OCH_3_), 3.87 (s, 6H, C23OCH_3_ and C25OCH_3_), 3.76 (s, 3H, C24OCH_3_), 3.40 (br m, 1H, H8), 3.32-3.25 (br m, 2H, H2A and H2B), 3.24 (br m, 1H, H3), 3.07 (br m, 1H, H6α, overlapped by HDO from solvent), 2.88 (br m, 1H, H6β), 2.20 (br m, 1H, H4), 1.88 (br m, 1H, H7α), 1.79 (br m, 1H, H5α), 1.73 (br m, 1H, H5β), 1.59 (br m, 1H, H7β); ^13^C-NMR (DMSO-*d*_6_ at 45 °C): 188.5 (C20), 157.7 (C6′), 153.5 (C23 and C25), 150.7 (C10), 149.8 (C4′), 147.9 (C2′), 144.5 (C8a′), 143.2 (C24), 142.5 (C18), 138.2 (C12), 135.4 (C15), 133.4 (C21), 131.7 (C8′), 130.7 (C14 and C16), 127.1 (C4a′), 123.7 (C19), 121.5 (C7′), 120.7 (C11), 120.5 (C13 and C17), 119.6 (C3′), 107.4 (C22 and C26), 103.2 (C5′), 70.3 (C9), 60.7 (C24OCH_3_), 60.6 (C8), 57.0 (C23OCH_3_ and C25OCH_3_), 56.4 (C6′OCH_3_), 55.3 (C2), 42.6 (C6), 32.8 (C3), 27.6 (C4), 26.8 (C5), 26.5 (C7); HRMS exact mass calcd. for C_38_H_40_N_5_O_6_ [MH]^+^, requires *m*/*z*: 662.29731, found *m*/*z*: 662.29683.

(*E*)-3-{4-{4-{(1*S*,3*R*,4*S*,6*S*)-6-[(*S*)-Hydroxy(6-methoxyquinolin-4-yl)methyl]quinuclidin-3-yl}-1*H*-1,2,3-triazol-1-yl}phenyl}-1-(4-hydroxy-3,5-dimethylphenyl)prop-2-en-1-one (**7d**)

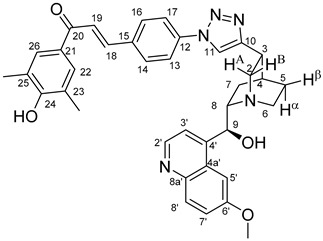

Yellow solid; Yield: 129 mg (21%); MP.: ~152 °C (dec., from diethyl ether); ^1^H-NMR (DMSO-*d*_6_ at 45 °C): 8.65 (d, *J* = 4.4 Hz, 1H, H2, overlapped by H11), 8.65 (s, 1H, H11, overlapped by H2′), 7.94 (d, *J* = 8.7 Hz, 2H, H16 and H14), 7.88 (d, *J* = 9.2 Hz, 1H, H8′), 7.84 (d, *J* = 15.6 Hz, 1H, H19), 7.81 (d, *J* = 8.7 Hz, 2H, H13 and H17), 7.74 (s, 2H, H22 and H26), 7.69 (d, *J* = 2.6 Hz, 1H, H5′), 7.61 (d, *J* = 15.6 Hz, 1H, H18), 7.55 (d, *J* = 4.4 Hz, 1H, H3′), 7.35 (dd, *J* = 9.2 Hz, 2.7 Hz, 1H, H7′), 6.02 (br s, 1H, H9), 3.98 (s, 3H, C6′OCH_3_), 3.85 (br m, 1H, H6α), 3.58 (br m, 1H, H8), 3.57 (br m, 1H, H2B), 3.48 (br m, 1H, H2A), 3.41 (br m, 1H, H3), 3.06 (br m, 1H, H6β), 2.28 (br m, 1H, H4), 2.04 (s, 6H, CH_3_), 2.02 (br m, 1H, H5α), 1.98 (br m, 1H, H7α), 1.88 (br m, 1H, H5β), 1.42 (br m, 1H, H7β); ^13^C-NMR (DMSO-*d*_6_ at 45 °C): 187.9 (C20), 158.8 (C24), 158.2 (C6′), 149.7 (C10), 147.9 (C2′), 147.1 (C4′), 144.5 (C8a′), 141.2 (C18), 137.9 (C12), 135.6 (C15), 131.8 (C8′), 130.4 (C14 and C16), 130.0 (C22 and C26), 129.6 (C21), 126.7 (C4a′), 124.9 (C23 and C25), 124.2 (C19), 122.0 (C7′), 121.1 (C11), 120.6 (C13 and C17), 119.5 (C3′), 103.0 (C5′), 67.9 (C9), 60.3 (C8), 57.1 (C6′OCH_3_), 54.4 (C2), 43.2 (C6), 32.0 (C3), 27.6 (C4), 25.4 (C5), 20.4 (C7), 16.9 (CH_3_); HRMS exact mass calcd. for C_37_H_38_N_5_O_4_ [MH]^+^, requires *m*/*z*: 616.29183, found *m*/*z*: 616.29159.

(*E*)-3-{2-{4-{(1*S*,3*R*,4*S*,6*R*)-6-[(*R*)-Hydroxy(6-methoxyquinolin-4-yl)methyl]quinuclidin-3-yl}-1*H*-1,2,3-triazol-1-yl}phenyl}-1-(2-methoxyphenyl)prop-2-en-1-one (**9a**)

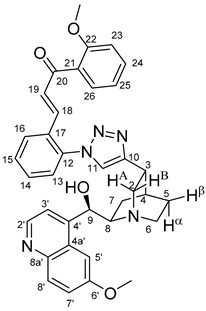

Yellow solid; Yield: 373 mg (62%); MP.: ~138 °C (dec., from diethyl ether); ^1^H-NMR (DMSO-*d*_6_ at 45 °C): 8.72 (s, 1H, H11), 8.64 (d, *J* = 4.4 Hz, 1H, H2′), 7.97 (d, *J* = 8.7 Hz, 1H, H26), 7.89 (d, *J* = 9.2 Hz, 1H, H8′), 7.96-7.92 (m, 3H, H14 and H15 and H16), 7.49 (d, *J* = 7.8 Hz, 1H, H13), 7.60 (d, *J* = 2.8 Hz, 1H, H5′), 7.54 (d, *J* = 16.0 Hz, 1H, H18), 7.50 (1H, H24, overlapped by H3′), 7.45 (1H, H3′, overlapped by H24), 7.44 (d, *J* = 16.0 Hz, 1H, H19), 7.35 (dd, *J* = 9.2 Hz, 2.7 Hz, 1H, H7′), 7.17 (d, *J* = 8.3 Hz, 1H, H23), 7.04 (t, *J* = 7.5 Hz, 1H, H25), 5.75–5.45 (br s, 2H, H9 and C9OH), 3.92 (s, 3H, C6′OCH_3_), 3.86 (s, 3H, C22OCH_3_), 3.72 (br m, 1H, H2A), 3.18 (br m, H8), 3.12 (1H, H2B, overlapped by HDO from solvent), 3.11 (1H, H3, overlapped by HDO from solvent), 2.83 (br m, 2H, H6α and H6β), 2.16 (br m, 1H, H4), 1.95 (br m, 1H, H7β), 1.68 (br m, 2H, H5α and H5β), 1.45 (br m, 1H, H7α); ^13^C-NMR (DMSO-*d*_6_ at 45 °C): 192.5 (C20), 158.4 (C22), 157.6 (C6′), 150.9 (C10), 149.3 (C4′), 147.9 (C2′), 144.5 (C8a′), 141.4 (C18), 138.3 (C12), 135.3 (C17), 133.5 (C24), 131.6 (C8′), 130.4 (C14 and C15), 130.0 (C13), 129.4 (C21), 128.6 (C19), 127.4 (C4a′), 121.5 (C7′), 121.1 (C25), 120.7 (C11 and C16 and C26), 119.6 (C3′), 113.1 (C23), 103.3 (C5′), 69.7 (C9), 60.8 (C8), 56.5 (C22OCH_3_), 56.2 (C6′OCH_3_), 49.4 (C6), 48.1 (C2), 32.9 (C3) 28.0 (C4), 25.6 (C5), 23.7 (C7); HRMS exact mass calcd. for C_36_H_36_N_5_O_4_ [MH]^+^, requires *m*/*z*: 602.27618, found *m*/*z*: 602.27540.

(*E*)-3-{2-{4-{(1*S*,3*R*,4*S*,6*R*)-6-[(*R*)-Hydroxy(6-methoxyquinolin-4-yl)methyl]quinuclidin-3-yl}-1*H*-1,2,3-triazol-1-yl}phenyl}-1-(4-methoxyphenyl)prop-2-en-1-one (**9b**)

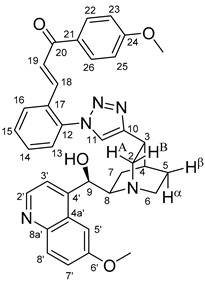

Yellow solid; Yield: 259 mg (43%); MP.: ~129 °C (dec., from diethyl ether); ^1^H-NMR (DMSO-*d*_6_ at 45 °C): 8.60 (d, *J* = 4.5 Hz, 1H, H2′), 8.40 (s, 1H, H11), 8.26 (m, 1H, H16), 8.04 (d, *J* = 8.9 Hz, 2H, H22 and H26), 7.88 (d, *J* = 9.2 Hz, 1H, H8′), 7.77 (d, *J* = 15.6 Hz, 1H, H19), 7.64−7.69 (m, 2H, H14 and H15), 7.60 (d, *J* = 7.1 Hz, 1H, H13), 7.59 (d, *J* = 2.7 Hz, 1H, H5′), 7.47 (d, *J* = 4.5 Hz, 1H, H3′), 7.36 (d, *J* = 15.6 Hz, 1H, H18), 7.34 (dd, *J* = 9.2 Hz, 2.6 Hz, 1H, H7′), 7.03 (d, *J* = 8.8 Hz, 2H, H23 and H25), 5.57 (br m, 1H, H9 and C9OH), 3.88 (s, 3H, C6′OCH_3_), 3.84 (s, 3H, C24OCH_3_), 3.69 (br m, 1H, H2A), 3.16 (br m, H8), 3.15 (1H, H3, overlapped by HDO from solvent), 3.12 (1H, H2B, overlapped by HDO from solvent), 2.81 (br m, 2H, H6α and H6β), 2.10 (br m, 1H, H4), 2.00 (br m, 1H, H7β), 1.66 (br m, 2H, H5α and H5β), 1.43 (br m, 1H, H7α); ^13^C-NMR (DMSO-*d*_6_ at 45 °C): 187.8 (C20), 164.0 (C24), 157.6 (C6′), 150.3 (C10), 148.8 (C4′), 147.9 (C2′), 144.5 (C8a′), 137.3 (C18), 137.0 (C12), 131.6 (C8′), 131.5 (C14), 131.4 (C22 and C26), 130.7 (C21), 130.6 (C15), 128.9 (C16), 127.4 (C4a′), 127.1 (C13), 125.9 (C17), 125.8 (C19), 125.1 (C11), 121.5 (C7′), 119.7 (C3′), 114.6 (C23 and C25), 103.2 (C5′), 70.4 (C9), 61.0 (C8), 56.2 (C6′OCH_3_), 56.1 (C24OCH_3_), 49.5 (C6), 48.4 (C2), 33.1 (C3) 28.6 (C4), 26.0 (C5), 23.7 (C7); HRMS exact mass calcd. for C_36_H_37_N_5_O_4_ [MH]^+^, requires *m*/*z*: 602.27618, found *m*/*z*: 602.27521.

(*E*)-3-{2-{4-{(1*S*,3*R*,4*S*,6*R*)-6-[(*R*)-Hydroxy(6-methoxyquinolin-4-yl)methyl]quinuclidin-3-yl}-1*H*-1,2,3-triazol-1-yl}phenyl}-1-(3,4,5-trimethoxyphenyl)prop-2-en-1-one (**9c**)

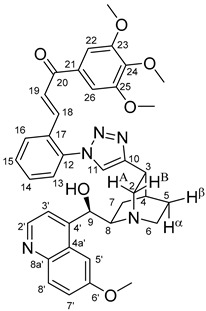

Yellow solid; Yield: 300 mg (50%); MP.: ~158 °C (dec., from diethyl ether); ^1^H-NMR (DMSO-*d*_6_ at 45 °C): 8.66 (br s, 1H, H2′), 8.45 (s, 1H, H11), 8.35 (m, 1H, H16), 7.93 (d, *J* = 9.1 Hz, 1H, H8′), 7.86 (d, *J* = 15.5 Hz, 1H, H19), 7.74 (1H, H15, overlapped by H14), 7.72 (1H, H14, overlapped by H15), 7.68 (m, 1H, H13), 7.62 (d, *J* = 2.5 Hz, 1H, H5′), 7.51 (d, *J* = 4.4 Hz, 1H, H3′), 7.43 (d, *J* = 15.6 Hz, 1H, H18), 7.38 (dd, *J* = 9.1 Hz, 2.5 Hz, 1H, H7′, overlapped by H22 and H26), 7.38 (s, 2H, H22 and H26, overlapped by H7′), 5.61 (br s, 1H, C9OH), 5.56 (br m, 1H, H9), 3.92 (s, 3H, C6′OCH_3_), 3.89 (s, 6H, C23OCH_3_ and C25OCH_3_), 3.79 (s, 3H, C24OCH_3_), 3.78 (br m, 1H, H8), 3.73 (br m, 1H, H2A), 3.15 (1H, H3, overlapped by HDO from solvent), 3.13 (1H, H2B, overlapped by HDO from solvent), 2.84 (br m, 1H, H6α), 2.80 (br m, 1H, H6β), 2.12 (br m, 1H, H4), 2.00 (br m, 1H, H7β), 1.47 (br m, 1H, H7α), 1.69 (br m, 2H, H5α and H5β); ^13^C-NMR (DMSO-*d*_6_ at 45 °C): 188.3 (C20), 157.4 (C6′), 153.5 (C23 and C25), 149.2 (C10), 147.9 (C2′), 146.8 (C4′), 144.5 (C8a′), 143.3 (C24), 137.9 (C18), 137.1 (C12), 133.1 (C21), 131.7 (C8′ and C14), 131.0 (C17), 130.7 (C15), 128.9 (C16), 127.2 (C13), 127.5 (C4a′), 125.7 (C19), 125.0 (C11), 121.4 (C7′), 119.6 (C3′), 107.3 (C22 and C26), 103.2 (C5′), 68.7 (C9), 60.8 (C8), 56.9 (C23OCH_3_ and C24OCH_3_ and C25OCH_3_), 56.1 (C6′OCH_3_), 49.6 (C6), 48.5 (C2), 33.4 (C3) 28.8 (C4), 26.2 (C5), 23.7 (C7); HRMS exact mass calcd. for C_38_H_40_N_5_O_6_ [MH]^+^, requires *m*/*z*: 662.29731, found *m*/*z*: 662.29652.

(*E*)-3-{2-{4-{(1*S*,3*R*,4*S*,6*R*)-6-[(*S*)-Hydroxy(6-methoxyquinolin-4-yl)methyl]quinuclidin-3-yl}-1*H*-1,2,3-triazol-1-yl}phenyl}-1-(3,4,5-trimethoxyphenyl)prop-2-en-1-one (9*c)

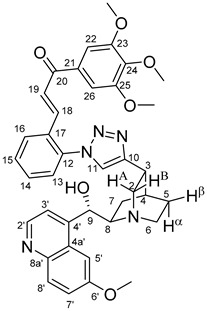

Yellow solid; Yield: 102 mg (17%); MP.: ~151 °C (dec., from diethyl ether); ^1^H-NMR (DMSO-*d*_6_ at 45 °C): 8.66 (br s, 1H, H2′), 8.48 (s, 1H, H11), 8.36 (m, 1H, H16), 7.95 (d, *J* = 9.2 Hz, 1H, H8′), 7.88 (d, *J* = 15.6 Hz, 1H, H19), 7.75 (1H, H15, overlapped by H14), 7.74 (1H, H14, overlapped by H15), 7.66 (m, 1H, H13), 7.61 (d, *J* = 2.5 Hz, 1H, H5′), 7.55 (d, *J* = 4.3 Hz, 1H, H3′), 7.42 (d, *J* = 15.6 Hz, 1H, H18, overlapped by H7′), 7.41 (dd, *J* = 9.1 Hz, 2.5 Hz, 1H, H7′, overlapped by H18), 7.39 (s, 2H, H22 and H26), 6.00 (br s, 1H, C9OH, overlapped by H9), 5.96 (br m, 1H, H9, overlapped by C9OH), 4.13 (br m, 1H, H2A), 3.98 (s, 3H, C6′OCH_3_), 3.89 (s, 6H, C23OCH_3_ and C25OCH_3_), 3.79 (s, 3H, C24OCH_3_), 3.47 (1H, H2B, overlapped by HDO from solvent), 3.40 (2H, H8 and H3, overlapped by HDO from solvent), 3.18 (br m, 1H, H6α), 3.10 (br m, 1H, H6β), 2.25 (br m, 1H, H4), 2.19 (br m, 1H, H7β), 1.95 (br m, 1H, H7α), 1.86 (br m, 1H, H5α), 1.79 (br m, 1H, H5β); ^13^C-NMR (DMSO-*d*_6_ at 45 °C): 188.3 (C20), 157.9 (C6′), 153.5 (C23 and C25), 149.2 (C10), 147.8 (C2′), 147.2 (C4′), 144.4 (C8a′), 143.2 (C24), 137.7 (C18), 137.0 (C12), 133.0 (C21), 131.7 (C8′ and C14), 131.0 (C17), 130.7 (C15), 129.0 (C16), 127.2 (C13), 126.8 (C4a′), 125.8 (C19), 125.2 (C11), 121.8 (C7′), 119.6 (C3′), 107.3 (C22 and C26), 102.8 (C5′), 68.7 (C9), 65.3 (C8), 56.9 (C23OCH_3_ and C25OCH_3_), 56.6 (C6′OCH_3_), 56.5 (C24OCH_3_), 49.1 (C6), 48.1 (C2), 32.1 (C3) 28.1 (C4), 27.9 (C7), 24.3 (C5); HRMS exact mass calcd. for C_38_H_40_N_5_O_6_ [MH]^+^, requires *m*/*z*: 662.29731, found *m*/*z*: 662.29604.

(*E*)-3-{2-{4-{(1*S*,3*R*,4*S*,6*R*)-6-[(*R*)-Hydroxy(6-methoxyquinolin-4-yl)methyl]quinuclidin-3-yl}-1*H*-1,2,3-triazol-1-yl}phenyl}-1-(4-hydroxy-3,5-dimethylphenyl)prop-2-en-1-one (**9d**)

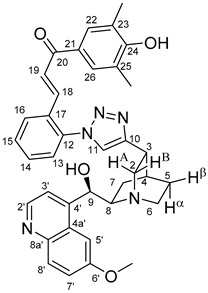

Orange solid; Yield: 226 mg (37%); MP.: ~174 °C (dec., from diethyl ether); ^1^H-NMR (DMSO-*d*_6_ at 45 °C): 8.59 (d, *J* = 4.4 Hz, 1H, H2′), 8.37 (s, 1H, H11), 8.24 (dd, *J* = 7.6 Hz, 2.5 Hz, 1H, H16), 7.88 (d, *J* = 9.2 Hz, 1H, H8′), 7.73 (d, *J* = 15.6 Hz, 1H, H19), 7.69 (s, 2H, H22 and H26), 7.66 (td, *J =* 7.3 Hz, 2.2 Hz, 1H, H14, overlapped by H15), 7.64 (td, *J* = 7.4 Hz, 2.1 Hz, 1H, H15, overlapped by H14), 7.58 (m, 2H, H5′ and H13), 7.45 (d, *J* = 4.4 Hz, 1H, H3′), 7.33 (dd, *J* = 9.1 Hz, 2.8 Hz, 1H, H7′), 7.32 (d, *J* = 15.6 Hz, 1H, H18), 5.54 (br s, 2H, H9 and C9OH), 3.88 (s, 3H, C6′OCH_3_), 3.80 (br m, 2H, H6α and H6β), 3.71 (br m, 1H, H2A), 3.16 (br m, 1H, H8, overlapped by HDO from solvent), 3.13 (br m, 1H, H3, overlapped HDO from solvent), 3.09 (br m, 1H, H2B), 2.21 (s, 6H, CH_3_), 2.09 (br m, 1H, H4), 1.98 (br m, 1H, H7β), 1.60–1.70 (br m, 2H, H5α and H5β), 1.44 (br m, 1H, H7α); ^13^C-NMR (DMSO-*d*_6_ at 45 °C): 187.8 (C20), 158.9 (C24), 157.5 (C6′), 150.4 (C10), 149.1 (C4′), 147.8 (C2′), 144.5 (C8a′), 137.0 (C12), 136.6 (C18), 131.6 (C8′), 131.3 (two coalesced lines, C14 and C17), 130.5 (C15), 130.0 (C22 and C26), 129.3 (C21), 128.8 (C16), 127.5 (C4a′), 127.1 (C13), 126.2 (C19), 125.0 (C11), 124.8 (C23 and C25), 121.4 (C7′), 119.5 (C3′), 103.2 (C5′), 70.8 (C9), 61.2 (C8), 56.1 (C6′OCH_3_), 49.5 (C6), 48.5 (C2), 33.3 (C3) 28.6 (C4), 26.3 (C5), 23.9 (C7), 16.9 (CH_3_); HRMS exact mass calcd. for C_37_H_38_N_5_O_4_ [MH]^+^, requires *m*/*z*: 616.29183, found *m*/*z*: 616.29137.

(*E*)-3-{2-{4-{(1*S*,3*R*,4*S*,6*R*)-6-[(*S*)-Hydroxy(6-methoxyquinolin-4-yl)methyl]quinuclidin-3-yl}-1*H*-1,2,3-triazol-1-yl}phenyl}-1-(4-hydroxy-3,5-dimethylphenyl)prop-2-en-1-one (9*d)

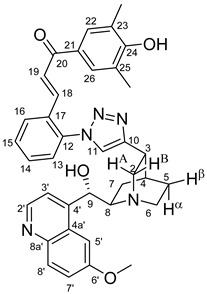

Yellow solid; Yield: 324 mg (53%); MP.: ~172 °C (dec., from diethyl ether); ^1^H-NMR (DMSO-*d*_6_ at 45 °C): 9.05 (br s, 1H, C24OH), 8.57 (d, *J* = 4.0 Hz, 1H, H2′), 8.42 (s, 1H, H11), 8.27 (dd, *J* = 6.9 Hz, 2.4 Hz, 1H, H16), 7.91 (d, *J* = 9.1 Hz, 1H, H8′), 7.78 (d, *J* = 15.6 Hz, 1H, H19), 7.72 (s, 2H, H22 and H26), 7.66–7.68 (m, 2H, H14 and H15), 7.64 (d, *J* = 2.6 Hz, 1H, H5′), 7.60 (dd, *J* = 7.6 Hz, 2.3 Hz, 1H, H13), 7.51 (d, *J* = 4.0 Hz, 1H, H3′), 7.36 (dd, *J* = 9.1 Hz, 2.6 Hz, 1H, H7′), 7.32 (d, *J* = 15.6 Hz, 1H, H18), 6.38 (br s, 1H, H9), 6.22 (br s, 1H, C9OH), 4.39 (br m, 1H, H2A), 4.00 (s, 3H, C6′OCH_3_), 3.69 (br m, 1H, H2B), 3.54 (br m, 1H, H3, overlapped by H8), 3.53 (br m, 1H, H8, overlapped by H3), 3.38 (br m, 1H, H6α), 3.26 (br m, 1H, H6β), 2.32 (br m, 1H, H7β, overlapped by H4), 2.31 (br m, 1H, H4, overlapped by H7β), 2.22 (s, 6H, CH_3_), 1.95 (br m, 1H, H5α), 1.86 (br m, 1H, H5β), 1.19 (br m, 1H, H7α); ^13^C-NMR (DMSO-*d*_6_ at 45 °C): 187.7 (C20), 159.0 (C24), 158.4 (C6′), 148.3 (C10), 147.8 (C2′), 145.9 (C4′), 144.4 (C8a′), 136.9 (C12), 136.3 (C18), 131.7 (C8′), 131.4 (C14), 130.7 (C15 and C17), 130.1 (C22 and C26), 129.3 (C21), 128.8 (C16), 127.2 (C13), 126.3 (C4a′ and C19), 125.3 (C11), 124.9 (C23 and C25), 122.2 (C7′), 119.5 (C3′), 102.6 (C5′), 66.9 (C9), 60.0 (C8), 59.9 (C6′OCH_3_), 48.9 (C6), 47.9 (C2), 31.5 (C3) 27.5 (C4), 23.3 (C5), 18.4 (C7), 16.9 (CH_3_); HRMS exact mass calcd. for C_37_H_38_N_5_O_4_ [MH]^+^, requires *m*/*z*: 616.29183, found *m*/*z*: 616.29097.

(*E*)-3-{4-{4-{(1*S*,3*R*,4*S*,6*R*)-6-[(*R*)-Hydroxy(6-methoxyquinolin-4-yl)methyl]quinuclidin-3-yl}-1*H*-1,2,3-triazol-1-yl}phenyl}-1-(2-methoxyphenyl)prop-2-en-1-one (**10a**)

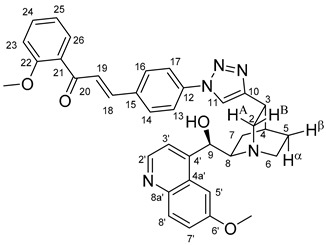

Yellow solid; Yield: 114 mg (19%); MP.: ~173 °C (dec., from diethyl ether); ^1^H-NMR (DMSO-*d*_6_ at 45 °C): 8.77 (s, 1H, H11), 8.65 (d, *J* = 4.5 Hz, 1H, H2′), 7.93-7.96 (m, 4H, H13 and H14 and H16 and H17), 7.91 (d, *J* = 9.2 Hz, 1H, H8′), 7.62 (d, *J* = 2.6 Hz, 1H, H5′), 7.54 (d, *J* = 16.0 Hz, 1H, H18), 7.48–7.53 (m, 3H, H3′ and H24 and H26), 7.45 (d, *J* = 16.0 Hz, 1H, H19), 7.37 (dd, *J* = 9.2 Hz, 2.6 Hz, 1H, H7′), 7.17 (d, *J* = 8.3 Hz, 1H, H23), 7.04 (td, *J* = 7.2 Hz, 0.9 Hz, 1H, H25), 6.42–6.08 (br s, 2H, H9 and C9OH), 3.98 (s, 3H, C6′OCH_3_), 3.86 (s, 3H, C22OCH_3_), 3.68-3.73 (br s, 2H, H6α and H6β), 3.52 (br s, 1H, H8), 3.43 (br s, 1H, H3), 3.20–3.34 (br s, 2H, H2A and H2B), 2.35 (br s, 1H, H4), 2.25 (br s, 1H, H7β), 1.84–1.90 (br s, 2H, H5α and H5β), 1.28 (br s, 1H, H7β); ^13^C-NMR (DMSO-*d*_6_ at 45 °C): 192.5 (C20), 158.4 (C22), 158.1 (C6′), 149.2 (C10), 147.8 (C2′), 146.0 (C4′), 144.4 (C8a′), 141.4 (C18), 138.2 (C12), 135.4 (C15), 133.5 (C24), 131.8 (C8′), 130.5 (C14 and C16), 130.0 (C26), 129.4 (C21), 128.7 (C19), 126.7 (C4a′), 122.0 (C7′), 121.1 (C25), 121.0 (C11), 120.8 (C13 and C17), 119.4 (C3′), 113.1 (C23), 102.8 (C5′), 66.2 (C9), 60.1 (C8), 57.0 (C6′OCH_3_), 56.5 (C22OCH_3_), 49.0 (C2), 47.9 (C6), 31.5 (C3), 27.1 (C4), 26.0 (C7), 23.3 (C5); HRMS exact mass calcd. for C_36_H_37_N_5_O_4_ [MH]^+^, requires *m*/*z*: 602.27618, found *m*/*z*: 602.27546.

(*E*)-3-{4-{4-{(1*S*,3*R*,4*S*,6*R*)-6-[(*R*)-Hydroxy(6-methoxyquinolin-4-yl)methyl]quinuclidin-3-yl}-1*H*-1,2,3-triazol-1-yl}phenyl}-1-(4-methoxyphenyl)prop-2-en-1-one (**10b**)

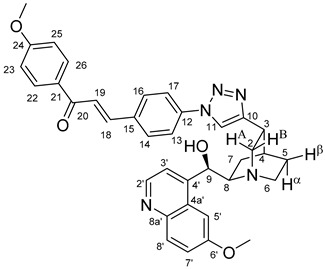

Yellow solid; Yield: 373 mg (62%); MP.: ~154 °C (dec., from diethyl ether); ^1^H-NMR (DMSO-*d*_6_ at 45 °C): 8.73 (s, 1H, H11), 8.64 (d, *J* = 4.5 Hz, 1H, H2′), 8.13 (d, *J* = 8.9 Hz, 2H, H22 and H26), 8.06 (d, *J* = 8.6 Hz, 2H, H14 and H16), 7.99 (d, *J* = 8.6 Hz, 2H, H13 and H17), 7.93 (d, *J* = 15.6 Hz, 1H, H19), 7.89 (d, *J* = 9.2 Hz, 1H, H8′), 7.73 (d, *J* = 15.6 Hz, 1H, H18), 7.60 (d, *J* = 2.7 Hz, 1H, H5′), 7.46 (d, *J* = 4.5 Hz, 1H, H3′), 7.35 (dd, *J* = 9.2 Hz, 2.7 Hz, 1H, H7′), 7.06 (d, *J* = 8.9 Hz, 2H, H23 and H25), 5.54 (br s, 1H, C9OH), 5.46 (br s, 1H, H9), 3.91 (s, 3H, C6′OCH_3_), 3.85 (s, 3H, C24OCH_3_), 3.65 (br s, 1H, H2A), 3.14 (1H, H8, overlapped by HDO from solvent), 3.06 (1H, H3, overlapped by HDO from solvent), 3.05 (1H, H2B, overlapped by HDO from solvent), 2.75 (br s, 2H, H6α and H6β), 2.13 (s, 1H, H4), 1.91 (s, 1H, H7β), 1.64 (m, 2H, H5α and H5β), 1.51 (s, 1H, H7α); ^13^C-NMR (DMSO-*d*_6_ at 45 °C): 188.0 (C20), 163.9 (C24), 157.5 (C6′), 151.2 (C10), 149.3 (C4′), 147.9 (C2′), 144.6 (C8a′), 142.0 (C18), 138.3 (C12), 135.4 (C15), 131.4 (C22 and C26), 131.6 (C8′), 131.1 (C21), 130.7 (C14 and C16), 129.4 (C4a′), 123.9 (C19), 121.4 (C7′), 121.0 (C11), 120.8 (C14 and C16), 119.6 (C3′), 114.6 (C23 and C25), 103.4 (C5′), 70.8 (C9), 61.2 (C8), 56.1 (C6′OCH_3_ and C24OCH_3_), 49.6 (C6), 48.3 (C2), 33.2 (C3), 28.2 (C4), 26.4 (C5), 24.2 (C7); HRMS exact mass calcd. for C_36_H_37_N_5_O_4_ [MH]^+^, requires *m*/*z*: 602.27618, found *m*/*z*: 602.27550.

(*E*)-3-{4-{4-{(1*S*,3*R*,4*S*,6*R*)-6-[(*R*)-Hydroxy(6-methoxyquinolin-4-yl)methyl]quinuclidin-3-yl}-1*H*-1,2,3-triazol-1-yl}phenyl}-1-(3,4,5-trimethoxyphenyl)prop-2-en-1-one (**10c**)

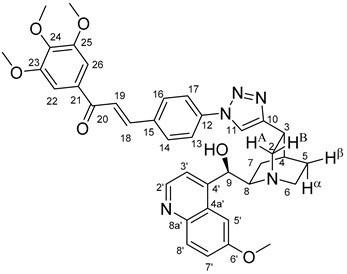

Yellow solid; Yield: 377 mg (57%); MP.: ~148 °C (dec., from diethyl ether); ^1^H-NMR (DMSO-*d*_6_ at 45 °C): 8.75 (s, 1H, H11), 8.65 (d, *J* = 4.5 Hz, 1H, H2′), 8.10 (d, *J* = 8.6 Hz, 2H, H14 and H16), 8.00 (d, *J* = 8.6 Hz, 2H, H13 and H17), 7.93 (d, *J* = 15.6 Hz, 1H, H19), 7.90 (d, *J* = 9.2 Hz, 1H, H8′), 7.77 (d, *J* = 15.6 Hz, 1H, H18), 7.61 (d, *J* = 2.7 Hz, 1H, H5′), 7.47 (d, *J* = 4.5 Hz, 1H, H3′), 7.41 (s, 2H, H22 and H26), 7.35 (dd, *J* = 9.2 Hz, 2.7 Hz, 1H, H7′), 5.66 (br s, 2H, C9OH and H9), 3.93 (s, 3H, C6′OCH_3_), 3.89 (s, 6H, C23OCH_3_ and C25OCH_3_), 3.77 (s, 3H, C24OCH_3_), 3.22 (br m, 1H, H8), 3.70 (br m, 1H, H2A), 3.17 (1H, H3, overlapped by HDO from solvent), 3.26 (1H, H2B, overlapped by HDO from solvent), 2.87 (br m, 2H, H6α and H6β), 2.18 (br m, 1H, H4), 1.99 (br m, 1H, H7β), 1.45 (br m, 1H, H7α), 1.70 (br m, 2H, H5α and H5β); ^13^C-NMR (DMSO-*d*_6_ at 45 °C): 188.5 (C20), 157.7 (C6′), 153.5 (C23 and C25), 150.7 (C10), 147.9 (C2′), 145.9 (C4′), 144.5 (C8a′), 143.2 (C24), 142.7 (C18), 138.4 (C12), 135.4 (C15), 133.4 (C21), 131.6 (C8′), 130.9 (C14 and C16), 127.3 (C4a′), 123.7 (C19), 121.7 (C7′), 120.8 (C11), 120.6 (C13 and C17), 119.6 (C3′), 107.4 (C22 and C26), 103.2 (C5′), 60.9 (C8), 60.7 (C24OCH_3_), 57.0 (C23OCH_3_ and C25OCH_3_), 56.3 (C6′OCH_3_), 49.5 (C6), 48.3 (C2), 32.1 (C3) 28.0 (C4), 25.8 (C5), 23.4 (C7); HRMS exact mass calcd. for C_38_H_40_N_5_O_6_ [MH]^+^, requires *m*/*z*: 662.29731, found *m*/*z*: 662.29668.

(*E*)-3-{4-{4-{(1*S*,3*R*,4*S*,6*R*)-6-[(*R*)-Hydroxy(6-methoxyquinolin-4-yl)methyl]quinuclidin-3-yl}-1*H*-1,2,3-triazol-1-yl}phenyl}-1-(4-hydroxy-3,5-dimethylphenyl)prop-2-en-1-one (**10d**)

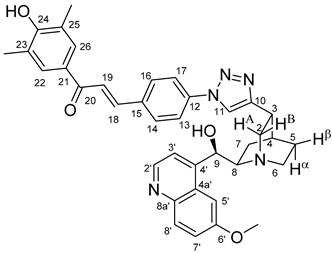

Yellow solid; Yield: 148 mg (24%); MP.: ~188 °C (dec., from diethyl ether); ^1^H-NMR (DMSO-*d*_6_ at 45 °C): 9.06 (br s, 3H, C24OH), 8.78 (s, 1H, H11), 8.64 (d, *J* = 4.5 Hz, 1H, H2′), 8.05 (d, *J* = 8.7 Hz, 2H, H14 and H16), 7.98 (d, *J* = 8.7 Hz, 2H, H13 and H17), 7.90 (d, *J* = 15.6 Hz, 1H, H19, overlapped by H8′), 7.90 (d, *J* = 9.2 Hz, 1H, H8′, overlapped by H19), 7.77 (s, 2H, H22 and H26), 7.68 (d, *J* = 15.6 Hz, 1H, H18, overlapped by H5′), 7.66 (1H, H5′, overlapped by H18), 7.49 (d, *J* = 4.5 Hz, 1H, H3′), 7.35 (dd, *J* = 9.2 Hz, 2.7 Hz, 1H, H7′), 6.11 (br s, 1H, H9), 6.05 (br s, 1H, C9OH), 4.14 (br m, 1H, H2A), 3.97 (s, 3H, C6′OCH_3_), 3.48 (br m, 1H, H2B), 3.40 (br m, 1H, H8), 3.35 (br m, 1H, H3), 3.18 (br m, 1H, H6α), 3.08 (br m, 1H, H6β), 2.29 (br m, 1H, H4), 2.24 (s, 6H, CH_3_), 2.19 (br m, 1H, H7β), 1.78–1.85 (br m, 2H, H5α and H5β), 1.34 (br m, 1H, H7α); ^13^C-NMR (DMSO-*d*_6_ at 45 °C): 188.0 (C20), 158.8 (C6′), 158.1 (C24), 149.7 (C10), 147.8 (C2′), 147.2 (C4′), 144.5 (C8a′), 141.2 (C18), 138.1 (C12), 135.8 (C15), 131.7 (C8′), 130.6 (C14 and C16), 130.0 (C22 and C26), 129.6 (C21), 126.8 (C4a′), 124.9 (C23 and C25), 124.2 (C19), 121.9 (C7′), 121.0 (C11), 120.6 (C13 and C17), 119.5 (C3′), 103.0 (C5′), 68.2 (C9), 60.5 (C8), 56.8 (C6′OCH_3_), 49.1 (C6), 48.0 (C2), 32.1 (C3) 27.7 (C4), 24.4 (C5), 20.6 (C7), 16.9 (CH_3_); HRMS exact mass calcd. for C_37_H_38_N_5_O_4_ [MH]^+^, requires *m*/*z*: 616.29183, found *m*/*z*: 616.29129.

(*E*)-3-{4-{5-{(1*S*,3*R*,4*S*,6*S*)-6-[(*S*)-Hydroxy(6-methoxyquinolin-4-yl)methyl]quinuclidin-3-yl}-1*H*-1,2,3-triazol-1-yl}phenyl}-1-(3,4,5-trimethoxyphenyl)prop-2-en-1-one (**11c**)

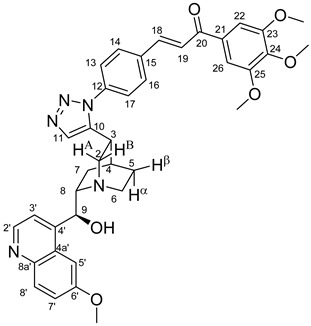

Brownish yellow solid; Yield: 264 mg (40%); MP.: ~152 °C (from diethyl ether); ^1^H-NMR (DMSO-*d*_6_ at 45 °C): 8.64 (d, *J* = 4.5 Hz, 1H, H2′), 8.06 (d, *J* = 8.4 Hz, 2H, H16 and H14), 7.95 (d, *J* = 15.6 Hz, 1H, H19), 7.89 (d, *J* = 9.2 Hz, 1H, H8′), 7.82 (s, 1H, H11), 7.76 (d, *J* = 15.6 Hz, 1H, H18), 7.59 (d, *J* = 8.4 Hz, 2H, H13 and H17), 7.49 (2H, H3′ and H5′), 7.41 (s, 2H, H22 and H26), 7.36 (dd, *J* = 9.2 Hz, 2.7 Hz, 1H, H7′), 5.54 (br s, 1H, C9OH), 5.27 (br s, 1H, H9), 3.88 (s, 3H, C6′OCH_3_), 3.87 (s, 6H, C23OCH_3_ and C25OCH_3_), 3.77 (s, 3H, C24OCH_3_), 3.25 (br s, 1H, H8), 3.13 (overlapped by HDO signal from solvent, 1H, H3), 1.85 (br s, 1H, H4), 1.68 (br s, 1H, H5α), 1.58 (t, *J* = 10.9 Hz, 1H, H7α), 1.38 (br s, 1H, H5β), 1.24 (m, 1H, H7β); ^13^C-NMR (DMSO-*d*_6_ at 45 °C): 188.4 (C20), 157.6 (C6′), 153.5 (C23 and C25), 147.9 (C2′), 144.5 (C8a′), 143.1 (C24), 142.5 (C18), 142.4 (C10), 138.1 (C12), 136.6 (C15), 133.3 (C21), 131.9 (C11), 131.7 (C8′), 130.4 (C14 and C16), 127.4 (C4a′), 126.4 (C13 and C17), 124.5 (C19), 121.3 (C7′), 119.7 (C3′), 107.3 (C22 and C26), 103.3 (C5′), 71.0 (C9), 60.7 (C8), 60.6 (C24OCH_3_), 57.0 (C23OCH_3_ and C25OCH_3_), 56.1 (C6′OCH_3_), 31.1 (C3), 26.6 (C4), 24.2 (C5), 22.0 (C7); HRMS exact mass calcd. for C_38_H_40_N_5_O_6_ [MH]^+^, requires *m*/*z*: 662.29731, found *m*/*z*: 662.29685.

(*E*)-3-{4-{5-{(1*S*,3*R*,4*S*,6*S*)-6-[(*S*)-Hydroxy(6-methoxyquinolin-4-yl)methyl]quinuclidin-3-yl}-1*H*-1,2,3-triazol-1-yl}phenyl}-1-(4-hydroxy-3,5-dimethylphenyl)prop-2-en-1-one (**11d**)

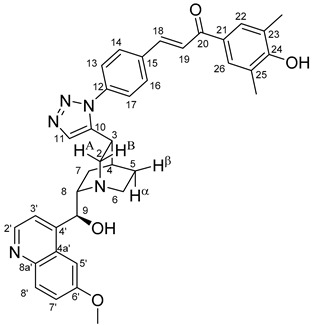

Brown solid; Yield: 193 mg (31%); MP.: ~176 °C (from diethyl ether); ^1^H-NMR (DMSO-*d*_6_ at 45 °C): 9.02 (br s, 1H, C24OH), 8.64 (d, *J* = 4.4 Hz, 1H, H2), 8.19 (d, *J* = 8.5 Hz, 2H, H14 and H16), 8.09 (s, 1H, H11), 7.92 (d, *J* = 15.5 Hz, 1H, H19), 7.89 (d, *J* = 9.2 Hz, 1H, H8′), 7.78 (s, 2H, H22 and H26), 7.68 (d, *J* = 15.5 Hz, 1H, H18), 7.49 (d, *J* = 4.5 Hz, 1H, H3′), 7.56 (d, *J* = 8.2 Hz, 2H, H13 and H17), 7.52 (d, *J* = 2.8 Hz, 1H, H5′), 7.35 (dd, *J* = 9.2 Hz, 2.7 Hz, 1H, H7′), 5.61 (br s, 1H, C9OH), 5.33 (br s, 1H, H9), 3.89 (s, 3H, C6′OCH_3_), 2.90-3.20 (6H, H2A and H2B and H3 and H6α and H6β and H8, overlapped by HDO signal from solvent), 2.23 (s, 6H, CH_3_), 1.83 (br s, 1H, H4), 1.68 (br s, 1H, H5α), 1.56–1.59 (2H, H5β and H7α), 1.37 (br m, 1H, H7β); ^13^C-NMR (DMSO-*d*_6_ at 45 °C, chemical shifts were determined from ^1^H-^13^C HSQC- and ^1^H-^13^C HMBC spectra): 187.9 (C20), 158.7 (C24), 157.5 (C6′), 147.9 (C2′), 144.5 (C8a′), 141.1 (C18), 139.8 (C10), 138.1 (C12), 136.8 (C15), 131.8 (C11), 131.6 (C8′), 130.2 (C14 and C16), 130.1 (C22 and C26), 129.5 (C21), 127.2 (C4a′), 126.4 (C13 and C17), 124.9 (C19 and C23 and C25), 121.4 (C7′), 119.7 (C3′), 103.3 (C5′), 70.1 (C9), 60.5 (C8), 56.2 (C6′OCH_3_), 54.4 (C2), 42.1 (C6), 31.0 (C3), 26.6 (C4), 25.3 (C5), 23.2 (C7), 16.9 (CH_3_); HRMS exact mass calcd. for C_37_H_38_N_5_O_4_ [MH]^+^, requires *m*/*z*: 616.29183, found *m*/*z*: 616.29165.

(*E*)-3-{4-{5-{(1*S*,3*R*,4*S*,6*R*)-6-[(*R*)-Hydroxy(6-methoxyquinolin-4-yl)methyl]quinuclidin-3-yl}-1*H*-1,2,3-triazol-1-yl}phenyl}-1-(3,4,5-trimethoxyphenyl)prop-2-en-1-one (**12c**)

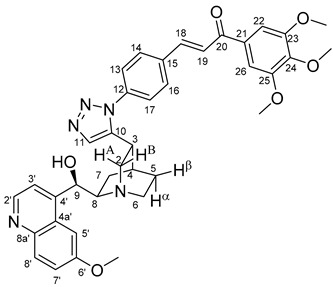

Yellow solid; Yield: 400 mg (60%); MP.: ~134 °C (dec., from diethyl ether); ^1^H-NMR (DMSO-*d*_6_ at 45 °C): 8.64 (d, *J* = 4.5 Hz, 1H, H2′), 8.07 (d, *J* = 8.4 Hz, 2H, H14 and H16, overlapped by H11), 8.06 (s, 1H, H11, overlapped by H14 and H16), 7.95 (d, *J* = 15.6 Hz, 1H, H19), 7.89 (d, *J* = 9.1 Hz, 1H, H8′), 7.78 (d, *J* = 15.6 Hz, 1H, H18), 7.62 (d, *J* = 8.4 Hz, 2H, H13 and H17), 7.49 (d, *J* = 4.5 Hz, 1H, H3′), 7.41 (s, 2H, H22 and H26), 7.38 (d, *J* = 2.7 Hz, 1H, H5′), 7.32 (dd, *J* = 9.2 Hz, 2.7 Hz, 1H, H7′), 5.61 (br s, 2H, C9OH), 5.34 (br s, 1H, H9), 3.87 (s, 6H, C23OCH_3_ and C25OCH_3_), 3.83 (s, 3H, C6′OCH_3_), 3.76 (s, 3H, C24OCH_3_), 3.31 (br m, 1H, H2A), 3.04 (t, *J* = 8.8 Hz, 1H, H3), 2.97 (qua, *J* = 7.2 Hz, 1H, H8), 2.81 (m, 1H, H2B), 2.64 (br m, 1H, H6α), 2.55 (m, 1H, H6β), 1.92 (dd, *J* = 12.6 Hz, 10.3 Hz, 1H, H7β), 1.85 (br s, 1H, H4), 1.44 (br m, 1H, H5β), 1.32 (br m, 2H, H7α and H5α); ^13^C-NMR (DMSO-*d*_6_ at 45 °C): 188.3 (C20), 157.3 (C6′), 153.4 (C23 and C25), 141.3 (C10), 147.9 (C2′), 149.6 (C4′), 144.2 (C8a′), 143.1 (C24), 142.6 (C18), 138.1 (C12), 136.4 (C15), 133.3 (C21), 131.6 (C8′), 130.5 (C14 and C16), 127.3 (C4a′), 124.1 (C19), 121.5 (C7′), 132.3 (C11), 126.3 (C13 and C17), 119.3 (C3′), 106.8 (C22 and C26), 102.8 (C5′), 71.1 (C9), 60.8 (C8), 60.7 (C24OCH_3_), 56.7 (C23OCH_3_ and C25OCH_3_), 55.9 (C6′OCH_3_), 49.2 (C6), 49.0 (C2), 31.5 (C3) 27.5 (C4), 26.3 (C5), 22.7 (C7); HRMS exact mass calcd. for C_38_H_40_N_5_O_6_ [MH]^+^, requires *m*/*z*: 662.29731, found *m*/*z*: 662.29687.

(*E*)-3-{4-{5-{(1*S*,3*R*,4*S*,6*R*)-6-[(*R*)-Hydroxy(6-methoxyquinolin-4-yl)methyl]quinuclidin-3-yl}-1*H*-1,2,3-triazol-1-yl}phenyl}-1-(4-hydroxy-3,5-dimethylphenyl)prop-2-en-1-one (**12d**)

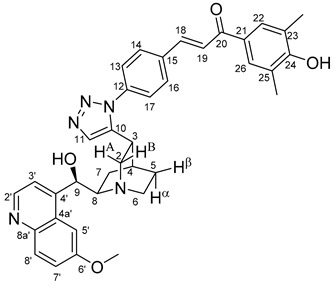

Yellow solid; Yield: 317 mg (51%); MP.: ~177 °C (dec., from diethyl ether); ^1^H-NMR (DMSO-*d*_6_ at 45 °C): 9.03 (br s, 1H, C24OH), 8.65 (d, *J* = 4.4 Hz, 1H, H2′), 8.09 (s, 1H, H11), 8.04 (d, *J* = 8.5 Hz, 2H, H14 and H16), 7.92 (d, *J* = 15.6 Hz, 1H, H19), 7.89 (d, *J* = 9.2 Hz, 1H, H8′), 7.78 (s, 2H, H22 and H26), 7.70 (d, *J* = 15.6 Hz, 1H, H18), 7.60 (d, *J* = 8.5 Hz, 2H, H13 and H17), 7.51 (d, *J* = 4.5 Hz, 1H, H3′), 7.42 (br s, 1H, H5′), 7.34 (dd, *J* = 9.2 Hz, 2.3 Hz, 1H, H7′), 5.83 (br s, 1H, C9OH), 5.58 (s, 1H, H9), 3.87 (s, 3H, C6′OCH_3_), 3.53 (br s, 1H, H2A), 3.15 (1H, H3, overlapped by HDO signal from solvent), 3.11 (1H, H8, overlapped by HDO from solvent), 3.00 (m, 1H, H2B), 2.80 (m, 1H, H6α), 2.70 (m, 1H, H6β), 2.23 (s, 6H, CH_3_), 2.02 (dd, *J* = 12.6 Hz, 9.2 Hz, 1H, H7β), 1.90 (s, 1H, H4), 1.53 (q, *J* = 9.2 Hz, 1H, H5α), 1.40 (t, *J* = 10.3 Hz, 1H, H5β), 1.29 (m, 1H, H7α); ^13^C-NMR (DMSO-*d*_6_ at 45 °C): 187.9 (C20), 158.9 (C24), 157.6 (C6′), 147.9 (C2′), 144.4 (C8a′), 141.1 (C18), 140.8 (C10), 137.7 (C12), 136.9 (C15), 132.3 (C11), 131.6 (C8′), 130.2 (C14 and C16), 130.1 (C22 and C26), 129.5 (C21), 127.1 (C4a′), 126.4 (C13 and C17), 124.9 (C23 and C25), 124.8 (C19), 121.5 (C7′), 119.7 (C3′), 103.0 (C5′), 70.2 (C9), 60.5 (C8), 56.2 (C6′OCH_3_), 49.0 (C6), 48.8 (C2), 31.1 (C3), 27.3 (C4), 25.5 (C5), 21.6 (C7), 16.9 (CH_3_); HRMS exact mass calcd. for C_38_H_40_N_5_O_6_ [MH]^+^, requires *m*/*z*: 616.29183, found *m*/*z*: 616.29137.

### 3.5. Cell Cultures

The PANC-1 (human pancreatic carcinoma of ductal origin), COLO 205 (human colorectal adenocarcinoma), A2058 (human metastatic melanoma) obtained from European Collection of Authenticated Cell Cultures (ECACC, Salisbury, UK) and EBC-1 (human lung squamous cell carcinoma) purchased from Japanese Research Resources Bank (Tokyo, Japan) were used to determine the tumor growth inhibitory effects of the cinchona alkaloid derivatives.

PANC-1 cells were maintained in Dulbecco’s Modified Eagle Medium (DMEM, Lonza, Basel, Switzerland); for the culturing of COLO-205 cell line DMEM medium formulated with 4500 mg/L d-glucose was used; EBC-1 cells were cultured in DMEM medium containing 1% non-essential amino acids (NEAA, Gibco^®^/Invitrogen Corporation, New York, NY, USA), 1 mM sodium pyruvate (Sigma-Aldrich, St. Louis, MO, USA), while A2058 cell line was grown in RPMI 1640 (Lonza, Basel, Switzerland). In case of all cell lines, the aforementioned basal media were supplemented with 10% fetal bovine serum (FBS, Gibco^®^/Invitrogen Corporation, New York, NY, USA), l-glutamine (2 mmol/L) (Lonza, Basel, Switzerland) and 100 µg/mL penicillin/streptomycin (Gibco^®^/Invitrogen Corporation, New York, NY, USA). (Gibco^®^/Invitrogen Corporation, New York, NY, USA). All cell lines were cultivated under standard conditions (37 °C, humidified 5% CO_2_ atmosphere) in plastic culture dishes (Sigma-Aldrich, St. Louis, MO, USA or Eppendorf AG, Hamburg, Germany).

### 3.6. Viability Assays

#### 3.6.1. Impedance-Based Assay

The cytotoxicity experiments on PANC-1 cells was conducted using the impedance-based xCELLigence SP System (ACEA Biosciences, San Diego, CA, USA). A more detailed description of the basis of impedimetric measurement is given in our previous paper [20]. Monitoring the impedance change, which is proportional to the number of adhered cells on an electrode surface, provides a sensitive way for cytotoxicity studies [21]. The change in the impedance is expressed as in the form of Cell Index (CI) calculated by the software (RTCA 2.0, ACEA Biosciences, San Diego, CA, USA) integrated to xCELLigence System.

For determination of IC_50_ (a concentration that decreases the cell viability by 50%) values the tested cinchona alkaloid-chalcones were solved in DMSO and further diluted in supplemented DMEM medium to prepare a concentration range from 2.5 × 10^−4^ to 5 × 10^−7^ M.

The steps of our impedimetric experiment proceeded in the same way as what was indicated in [22]. In brief, after gaining a constant CI value during the background measurement, the PANC-1 cells (1.5 × 10^4^ cells/well) were added to the so-called E-plate, and their adhesion/spreading was monitored for 24 h in order to settle the plateau phase of cell culture. In the last step, the cells in this balanced state were treated with the test compounds (final concentrations: 2.5 × 10^−5^ to 5 × 10^−8^ M) and the changes in CI were monitored for at least 72 hours at 10 kHz. In case of the control wells, the adequate volume ratio of DMSO was added. Three parallels were measured for each measurement. The CI values of each concentration obtained at 24, 48 and 72 h after the treatment were normalized to that of the DMSO control. The IC_50_ value was calculated for these normalized CI values by fitting a sigmoidal dose-response curve with the nonlinear regression function of OriginPro 8 (OriginLab Corporation, Northampton, MA, USA).

#### 3.6.2. Colorimetric Assay

The antiproliferative/cytotoxic effects of the cinchona alkaloid-chalcones on COLO-205, A2058 and EBC-1 cell lines were measured by the alamarBlue-assay. This colometric assay proved to be the more suitable method for the analysis of these cell lines than the xCELLigence System, because EBC-1 and COLO-205 cells show weak/negligible adhesion, while A2058 cell line fails to establish a stable plateau phase during the impedimetric analysis.

The inoculation of the cells and the procedure of alamarBlue-assay were analogous to the description reported in our previous paper [23]. The main steps are the following: (i) cell seeding on 96-well plates (Sarstedt AG, Nümbrecht, Germany) at 10^4^ cells/well concentration, (ii) treatment with the test substances at 2.5 × 10^−5^ to 5 × 10^−8^ M final concentrations for 24, 48 and 72 h, (iii) addition of alamarBlue reagent (0.15 mg/mL, Sigma-Aldrich, St. Louis, MO, USA) solved in PBS (phosphate-buffered saline, pH = 7.2) and (iv) reading the fluorescence intensity of the samples after 6–8 h incubation with alamarBlue reagent. LS-50B Luminescence Spectrometer (Perkin Elmer Ltd., Buckinghamshire United Kingdom) was applied for the fluorescence measurements with the following settings: excitation wavelength = 560 nm and emission wavelength = 590 nm. Each measurement was done in triplicates. Wells containing adequate volume ratio of DMSO served as control. The fluorescence intensity of each sample was expressed as a ratio of the fluorescence of DMSO control. The nonlinear regression function of OriginPro 8 (OriginLab Corporation, Northampton, MA, USA) was used for fitting sigmoidal dose-response curves to the normalized fluorescence intensities in order to calculate the IC_50_ values.

### 3.7. Cell Cycle Analysis

In order to determine the effect of cinchona-chalcone hybrids on the cell cycle progression of PANC-1 cells, the cellular samples of the different treatment groups were stained with propidium iodide (Sigma-Aldrich, St. Louis, MO, USA), which intercalates stoichiometrically to the double-stranded DNA. The DNA content of the cells was measured by FACSCalibur flow cytometer (Becton Dickinson, San Jose, CA, USA).

The steps of the sample preparation and the flow cytometric analysis were essentially the same as that used in our previous study [23] with some changes. PANC-1 cells in 2.5 x 10^5^ cells/well density were prepared in 12-well plate 24 h before the treatment. For the treatment, the most potent **12c** and a substantially less active hybrid (**10b**) were selected and their IC_50_ values (**12c**: 2.26 µM and **10b**: 13.30 µM), determined in the impedimetric assay after 72 h incubation, were used. After 72 h incubation with the compounds, the cells were washed twice with PBS and dissociated by trypsin/EDTA solution (Sigma-Aldrich, St. Louis, MO, USA). This step was followed by centrifugation; then, the cells were fixed in ice-cold 70% ethanol and stored at −20 °C for at least 24 h. To discard the fixative, the samples were centrifuged and resuspended in citric acid/sodium phosphate buffer (pH = 7.8) supplemented with RNase (100 μg/mL; Sigma-Aldrich, St. Louis, MO, USA). The cells were stained with propidium iodide in 10 μg/mL final concentration; then instantly a cytometric measurement was performed by collecting at 25,000 cells/sample. Samples added with adequate volume ratio of DMSO or with pure cell culture medium served as controls.

The cell cycle analysis was carried out by using two parallels. CellQuest Pro and Flowing 2.5.1 (Turku Centre of Biotechnology, Turku, Finland) software were applied to analyze the data. For aggregate/debris discrimination, FL2-Width vs FL2-Area plot was used and the gated cells were displayed in FL2-Area histogram to assign percentage values to each population of cell cycle stages.

### 3.8. Statistical Evaluation of Data

In case of the impedance-based viability assay, data analysis was performed by using RTCA 2.0 (ACEA Biosciences, San Diego, CA, USA), while CellQuest Pro and Flowing 2.5.1 (Turku Centre of Biotechnology, Turku, Finland) software were used for the evaluation of the flow cytometric histograms. Further evaluation of the results was done MS Excel, OriginPro 8 (OriginLab Corporation, Northampton, MA, USA) software. Data obtained from each experiment represent mathematical averages and ±SD values. To assess the statistical significance, one-way ANOVA coupled with Tukey’s post hoc test was used. The standard deviations of IC_50_ parameters were also obtained with the sigmoidal curve fitting. The levels of significance are shown as follows: *: *p* < 0.05; **: *p* < 0.01; ***: *p* < 0.001.

## 4. Conclusions

By means of convergent reaction sequences terminated by well-established copper- and ruthenium-catalyzed azide-alkyne cycloadditions, a series of novel hybrids comprising cinchona and chalcone fragments tethered with triazole linkers were prepared and evaluated for their antiproliferative/cytotoxic activity on human malignant cell lines PANC-1, COLO-205, A2058 and EBC-1. A quinidine-based hybrid containing 1,5-disubstituted 1,2,3-triazole linker and an (*E*)-3-phenyl-1-(3,4,5-trimethoxyphenyl)propenone moiety (**12c**) was identified as the most potent model displaying substantial cytotoxicity on all the investigated cells characterised by IC_50_ values in a low-to-submicromolar range. Besides the structure-activity relationships (SAR) set up on the basis of the IC_50_ values, the results of comparative cell-cycle analyses in PANC-1 cells that disclosed extensive inhibitory effects of the most potent hybrid in subG1, S and G2/M phases, might also be utilized in rational structure-refinement in the development of more potent antiproliferative agents with enhanced activity, selectivity and established mechanism of action.

## Figures and Tables

**Table 1:** Designation and isolated yields of azidochalcones **2a**–**d** and **4a**–**d** prepared by the appropriate method.

Product	R^1^	R^2^	R^3^	R^4^	Method	Yield (%)
**2a**	OCH_3_	H	H	H	(i)	67
**2b**	H	H	OCH_3_	H	(i)	55
**2c**	H	OCH_3_	OCH_3_	OCH_3_	(i)	43
**2d**	H	CH_3_	OH	CH_3_	(ii)	25
**4a**	OCH_3_	H	H	H	(i)	70
**4b**	H	H	OCH_3_	H	(i)	75
**4c**	H	OCH_3_	OCH_3_	OCH_3_	(i)	75
**4d**	H	CH_3_	OH	CH_3_	(ii)	25

**Table 2:** Designation and isolated yields of cinchona-chalcone hybrids types **6**, **7**, **9** and **10** with 1,4-disubstituted [1,2,3]triazole linker (see: Scheme 2).

Product	R^1^	R^2^	R^3^	R^4^	Yield (%)
**6a**	OCH_3_	H	H	H	91
**6b**	H	H	OCH_3_	H	72
**6c**	H	OCH_3_	OCH_3_	OCH_3_	74
**6d**	H	CH_3_	OH	CH_3_	30
6*d	H	CH_3_	OH	CH_3_	50
**7a**	OCH_3_	H	H	H	56
**7b**	H	H	OCH_3_	H	79
**7c**	H	OCH_3_	OCH_3_	OCH_3_	61
**7d**	H	CH3	OH	CH_3_	21
**9a**	OCH_3_	H	H	H	62
**9b**	H	H	OCH_3_	H	43
**9c**	H	OCH_3_	OCH_3_	OCH_3_	50
9*c	H	OCH_3_	OCH_3_	OCH_3_	17
**9d**	H	CH_3_	OH	CH_3_	37
9*d	H	CH_3_	OH	CH_3_	53
**10a**	OCH_3_	H	H	H	19
**10b**	H	H	OCH3	H	62
**10c**	H	OCH_3_	OCH3	OCH_3_	57
**10d**	H	CH_3_	OH	CH_3_	24

**Table 3:** List of cinchona-chalcone hybrids with 1,5-disubstituted-1,2,3-triazole linker and yields.

Product	R^1^	R^2^	R^3^	Yield (%)
**11c**	OCH_3_	OCH_3_	OCH_3_	40
**11d**	CH_3_	OH	CH_3_	31
**12c**	OCH_3_	OCH_3_	OCH_3_	60
**12d**	CH_3_	OH	CH_3_	51

**Table 4:** Cytotoxic activity of cinchona-chalcone hybrids expressed in IC_50_ values on four human tumour cell lines.

Tested Compound	IC_50_ [µM] ^a^			
	PANC-1	COLO-205	A2058	EBC-1
**2c**	9.67 ± 0.95	3.61 ± 0.71	11.03 ± 1.46	2.51 ± 0.21
**2d**	43.18 ± 1.21	4.37 ± 0.98	10.84 ±1.52	2.74 ± 0.23
**4c**	23.35 ± 0.59	8.32 ± 0.75	23.42 ± 5.09	3.47 ± 0.44
**6a**	6.83 ± 0.65	3.85 ± 1.14	3.49 ± 0.01	2.01 ± 0.16
**6b**	8.48 ± 1.73	1.90 ± 0.11	5.87 ± 0.48	1.49 ± 0.33
**6c**	2.45 ± 0.12	1.09 ± 0.06	2.51 ± 0.06	0.95 ± 0.02
**6d**	9.77 ± 0.44	2.06 ± 0.12	4.92 ± 0.08	1.73 ± 0.20
6*d	6.95 ± 1.91	1.65 ± 0.10	3.90 ± 0.09	1.53 ± 0.23
**7a**	11.19 ±1.83	7.39 ± 0.64	4.42 ± 0.40	1.44 ± 0.03
**7b**	9.16 ± 0.51	6.54 ± 0.59	15.39 ± 2.26	4.15 ± 0.38
**7c**	2.43 ± 0.69	2.21 ± 0.12	2.64 ± 0.12	1.66 ± 0.30
**7d**	9.01 ± 1.58	4.46 ± 0.20	9.72 ± 0.54	7.80 ± 0.11
**9a**	5.74 ± 0.31	4.77 ± 0.63	2.93 ± 0.01	2.49 ± 0.07
**9b**	5.06 ± 0.38	1.54 ± 0.07	12.46 ± 1.98	2.93 ± 0.21
**9c**	6.59 ± 0.85	1.33 ± 0.40	3.44 ± 0.13	0.87 ± 0.04
**9d**	4.85 ± 0.18	1.81 ± 0.34	7.10 ± 0.27	1.58 ± 0.26
9*d	5.38 ± 0.35	1.42 ± 0.41	7.58 ± 0.16	1.61 ± 0.21
**10a**	4.74 ± 0.19	5.04 ± 0.64	2.48 ± 0.01	1.70 ± 0.06
**10b**	13.35 ±2.19	5.96 ± 0.53	18.87 ± 3.51	4.50 ± 0.12
**10c**	3.72 ± 0.81	1.04 ± 0.02	2.63 ± 0.04	1.89 ± 0.72
**10d**	4.44 ± 0.17	2.53 ± 0.14	4.50 ± 0.31	6.50 ± 0.80
**11c**	9.27 ± 0.33	3.56 ± 0.28	5.60 ± 0.79	2.99 ± 0.54
**11d**	2.75 ± 0.15	10.98 ± 3.16	15.60 ± 1.60	6.14 ± 0.18
**12c**	2.26 ± 0.22	0.90 ± 0.12	1.81 ± 0.18	0.55 ± 0.12
**12d**	9.46 ± 0.78	0.66 ± 0.02	1.42 ± 0.27	1.47 ± 0.48

^a^ IC_50_ value measured after a treatment of 72 h.

## Supplementary Materials

The following are available online; Copies of NMR spectra; experimental details of the cell culturing, the in vitro assays and cell cycle analyses.

## References

[1] Isah T. (2016). Anticancer Alkaloids from Trees: Development into Drugs. Pharmacogn. Rev..

[2] Kacprzak K., Ruszkowski P., Valentini L., Huczyński A. (2018). Cytotoxic and trypanocidal activities of cinchona alkaloid derivatives. Chem. Biol. Drug Des..

[3] Miller T.P., Chase E.M., Dorr R., Dalton W.S., Lam K.S., Salmon S.E. (1998). A phase I/II trial of paclitaxel for non-Hodgkin’s lymphoma followed by paclitaxel plus quinine in drug-resistant disease. Anti-Cancer Drugs.

[4] Lehnert M., Dalton W.S., Roe D., Emerson S., Salmon S.-E. (1991). Synergistic inhibition by verapamil and quinine of P-glycoprotein- mediated multidrug resistance in a human myeloma cell line model. Blood.

[5] Dorr R.T., Liddil J.D. (1991). Modulation of mitomycin C-induced multidrug resistance in vitro. Cancer Chemother. Pharmacol..

[6] Károlyi B.I., Bősze S., Orbán E., Sohár P., Drahos L., Gál E., Csámpai A. (2010). Acylated mono-, bis- and tris- Cinchona-Based Amines Containing Ferrocene or Organic Residues: Synthesis, Structure and in Vitro Antitumor Activity on Selected Human Cancer Cell Lines. Molecules.

[7] Kocsis L., Szabó I., Bősze S., Jernei T., Hudecz F., Csámpai A. (2015). Synthesis, structure and in vitro cytostatic activity of ferrocene—Cinchona hybrids. Bioorg. Med. Chem. Lett..

[8] Mahapatra D.K., Bharti S.K., Asati V. (2015). Anti-cancer chalcones: Structural and molecular target perspectives. Eur. J. Med. Chem..

[9] Srinivasan B., Johnson T.E., Lad R., Xing C. (2009). Structure−Activity Relationship Studies of Chalcone Leading to 3-Hydroxy-4,3′,4′,5′-tetramethoxychalcone and Its Analogues as Potent Nuclear Factor κB Inhibitors and Their Anticancer Activities. J. Med. Chem..

[10] Boumendjel A., Ronot X., Boutonnat J. (2009). Chalcones Derivatives Acting as Cell Cycle Blockers: Potential Anti Cancer Drugs?. Curr. Drug Targets.

[11] Hijova E. (2016). Bioactivity of chalcones. Bratisl. Med. J..

[12] Podolski-Renić A., Bősze S., Dinić J., Kocsis L., Hudecz F., Csámpai A., Pešić M. (2017). Ferrocene–cinchona hybrids with triazolyl-chalcone linkers act as pro-oxidants and sensitize human cancer cell lines to paclitaxel. Metallomics.

[13] Rostovtsev V.V., Green L.G., Fokin V.V., Sharpless K.B. (2002). A Stepwise Huisgen Cycloaddition Process: Copper(I)-Catalyzed Regioselective “Ligation” of Azides and Terminal Alkynes. Angew. Chem. Int. Ed..

[14] Caner H., Biedermann P.U., Agranat I. (2003). Conformational spaces of Cinchona alkaloids. Chirality.

[15] Becke A.D. (1993). Density-functional thermochemistry. III. The role of exact exchange. J. Chem. Phys..

[16] Lee C., Yang W., Parr R.G. (1988). Development of the Colle-Salvetti correlation-energy formula into a functional of the electron density. Phys. Rev. B.

[17] Stephens P.J., Devlin F.J., Chahalowsky C.F., Frisch M.J. (1994). Ab Initio Calculation of Vibrational Absorption and Circular Dichroism Spectra Using Density Functional Force Fields. J. Phys. Chem..

[18] Hehre W.J., Radom L., Schleyer P.V.R., Pople J.A. (1986). Ab Initio Molecular Orbital Theory.

[19] Frisch M.J., Trucks G.W., Schlegel H.B., Scuseria G.E., Robb M.A., Cheeseman J.R., Scalmani G., Barone V., Petersson G.A., Nakatsuji H. (2016). Gaussian 09.

[20] Lajkó E., Szabó I., Andódy K., Pungor A., Mező G., Kőhidai L. (2013). Investigation on chemotactic drug targeting (chemotaxis and adhesion) inducer effect of GnRH-III derivatives in Tetrahymena and human leukemia cell line. J. Pept. Sci..

[21] Urcan E., Haertel U., Styllou M., Hickel R., Scherthan H., Reichl F.X. (2010). Real-time xCELLigence impedance analysis of the cytotoxicity of dental composite components on human gingival fibroblasts. Dent. Mater..

[22] Bárány P., Oláh R.S., Kovács I., Czuczi T., Szabó C.L., Takács A., Lajkó E., Láng O., Kőhidai L., Schlosser G. (2018). Ferrocene-Containing Impiridone (ONC201) Hybrids: Synthesis, DFT Modelling, In Vitro Evaluation, and Structure–Activity Relationships. Molecules.

[23] Lajkó E., Spring S., Hegedüs R., Biri-Kovács B., Ingebrandt S., Mező G., Kőhidai L. (2018). Comparative cell biological study of in vitro antitumor and antimetastatic activity on melanoma cells of GnRH-III-containing conjugates modified with short-chain fatty acids. Beilstein J. Org. Chem..

