# Supplementary material for: Synthesis, Structure and In Vitro Cytotoxic Activity of Novel Cinchona—Chalcone Hybrids with 1,4-Disubstituted- and 1,5-Disubstituted 1,2,3-Triazole Linkers
Source: Molecules. 2019 Nov 11;24(22):4077. doi: 10.3390/molecules24224077 (PMC6891474; doi:10.3390/molecules24224077)

^{1}H-NMR spectra of **2a**

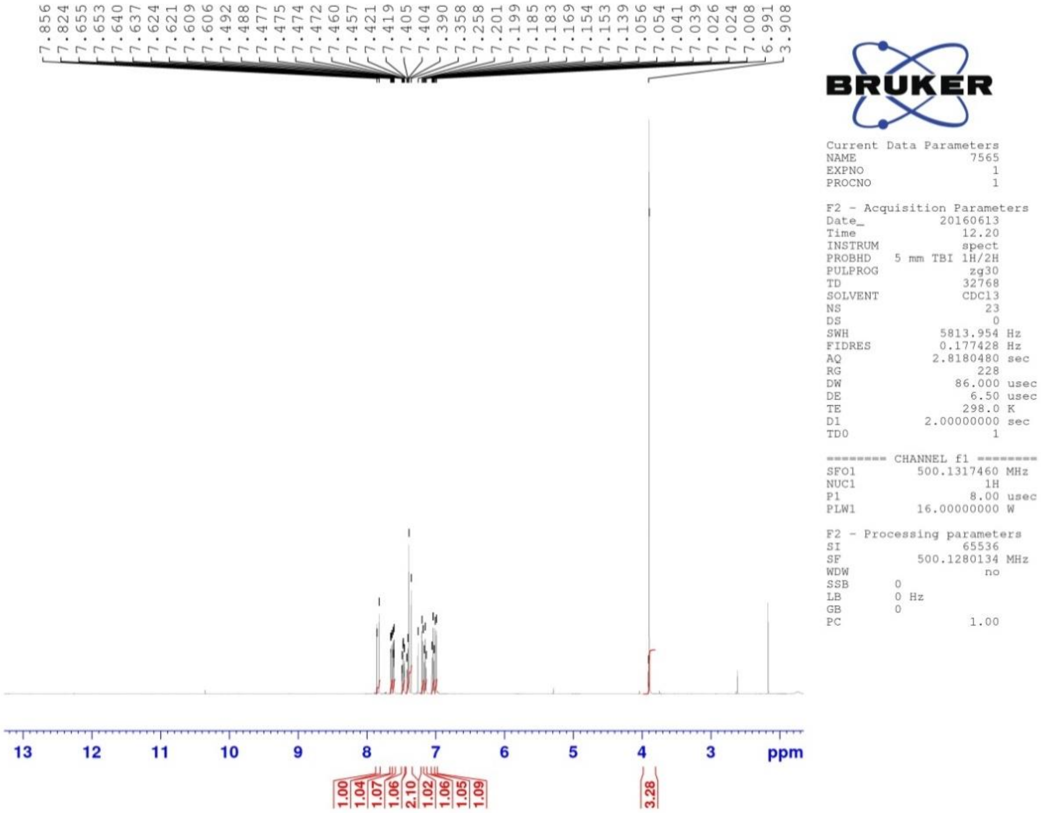

^{13}C-NMR spectra of **2a**

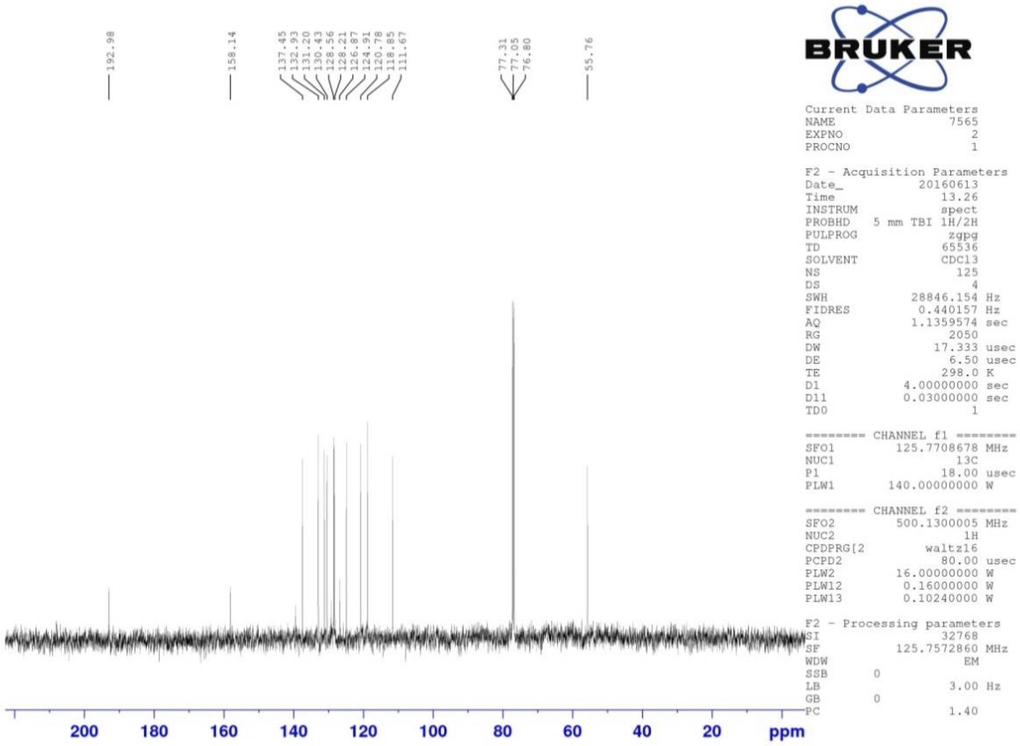

^{1}H-NMR spectra of **2b**

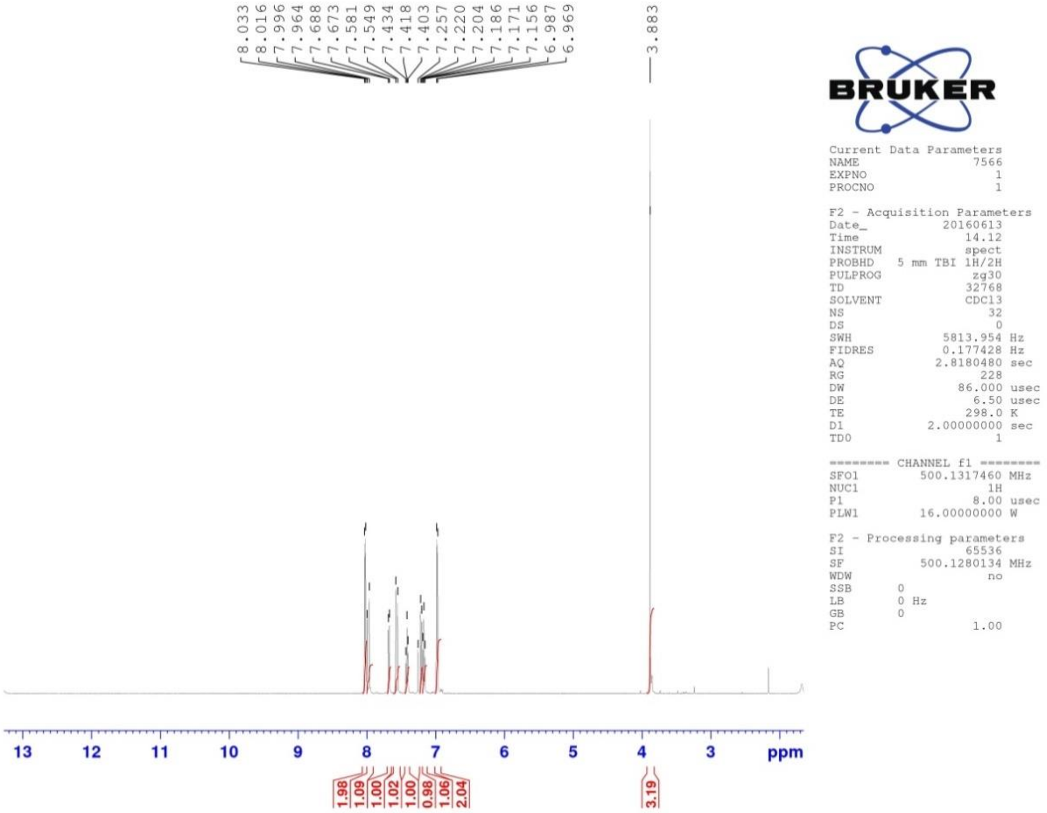

^{13}C-NMR spectra of **2b**

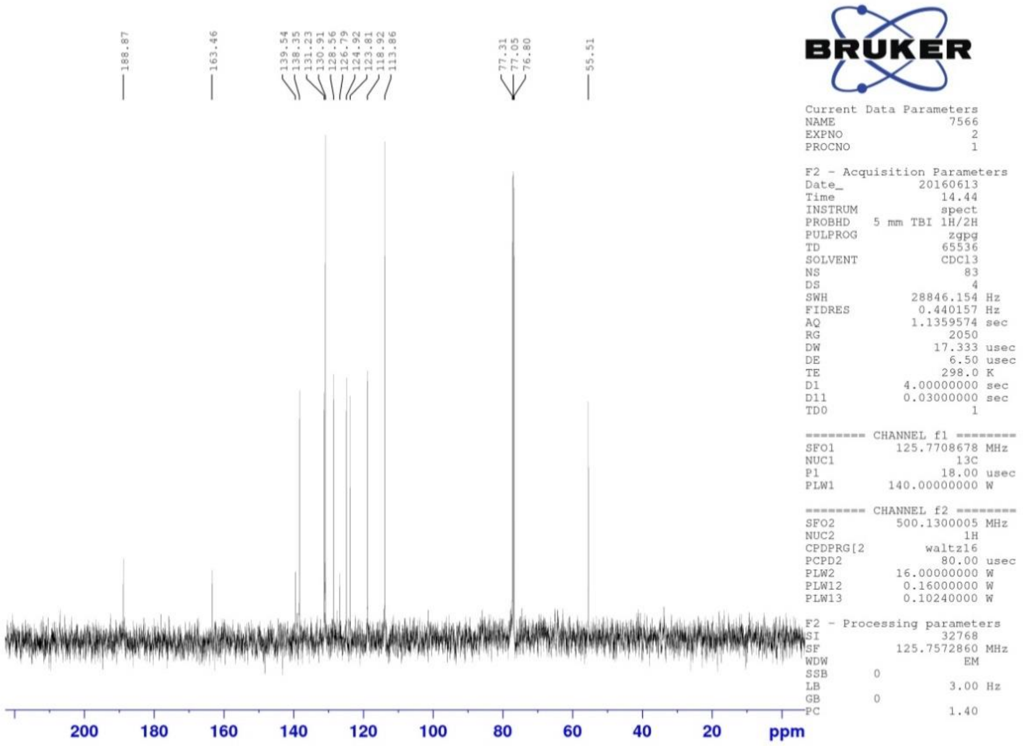

^{1}H-NMR spectra of **2c**

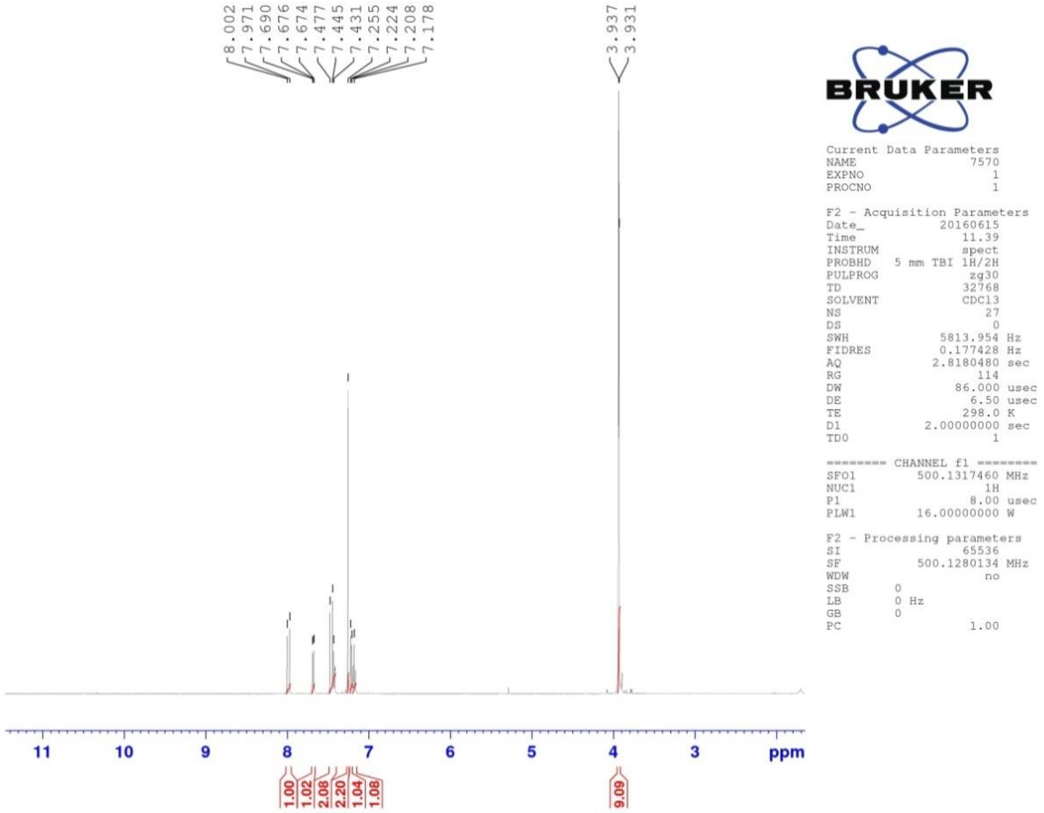

^{13}C-NMR spectra of **2c**

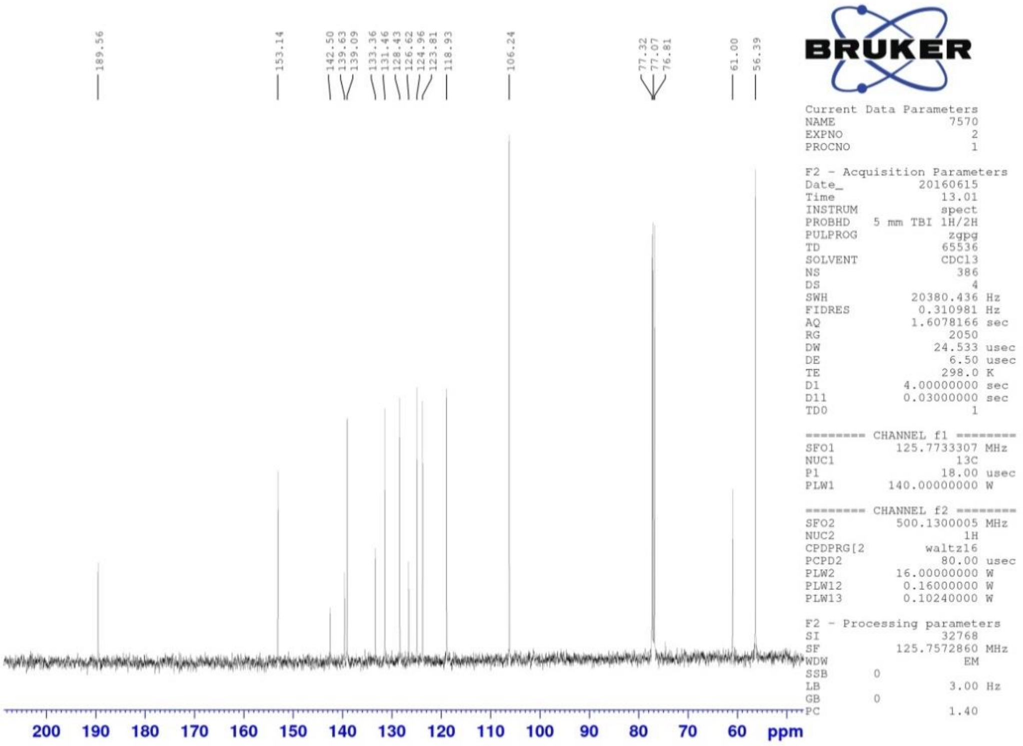

^{1}H-NMR spectra of **2d**

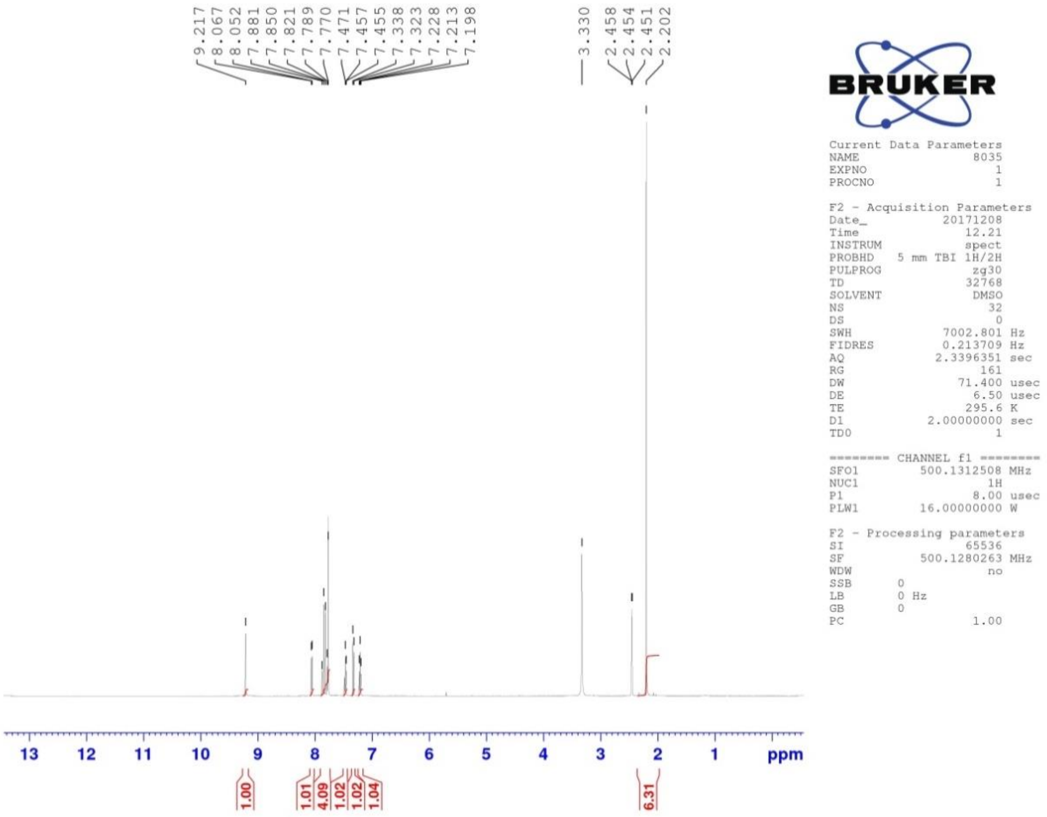

^{13}C-NMR spectra of **2d**

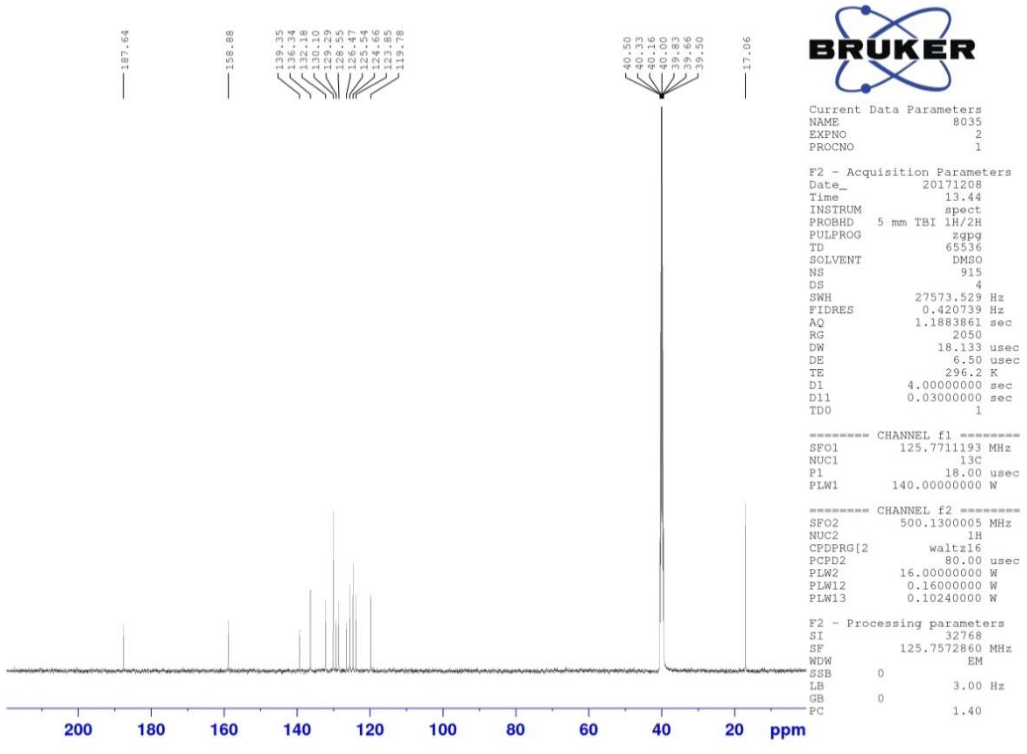

^{1}H-NMR spectra of **4a**

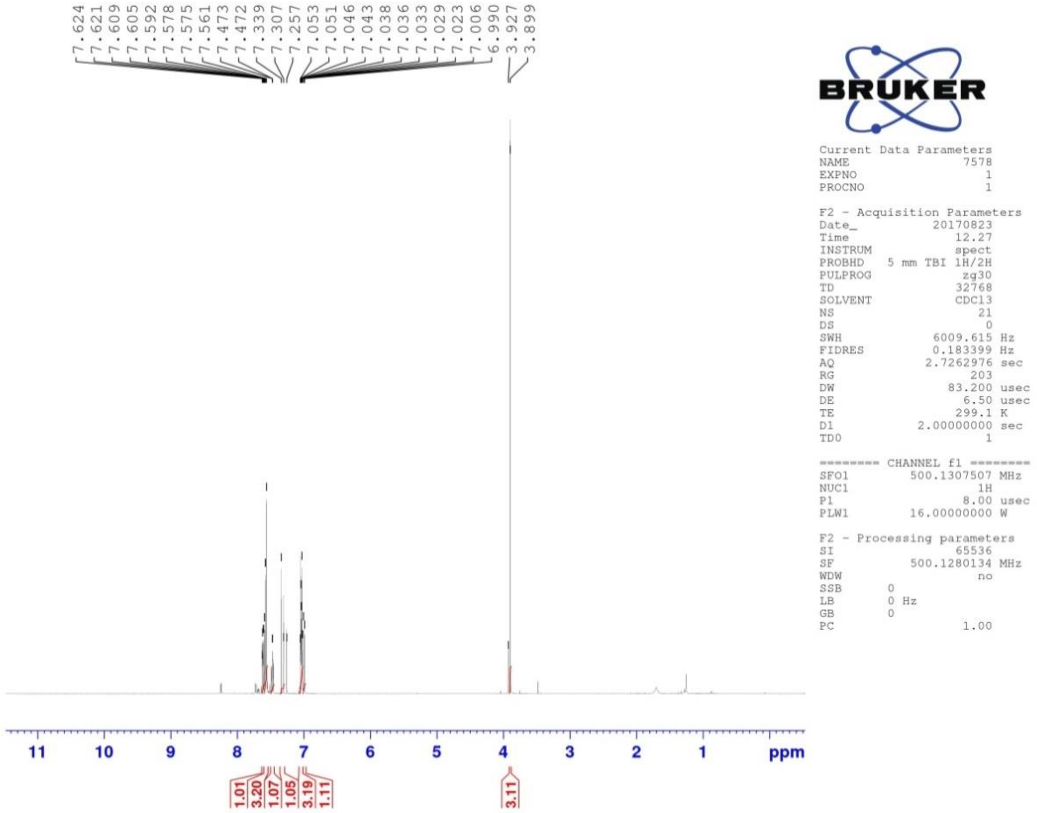

^{13}C-NMR spectra of **4a**

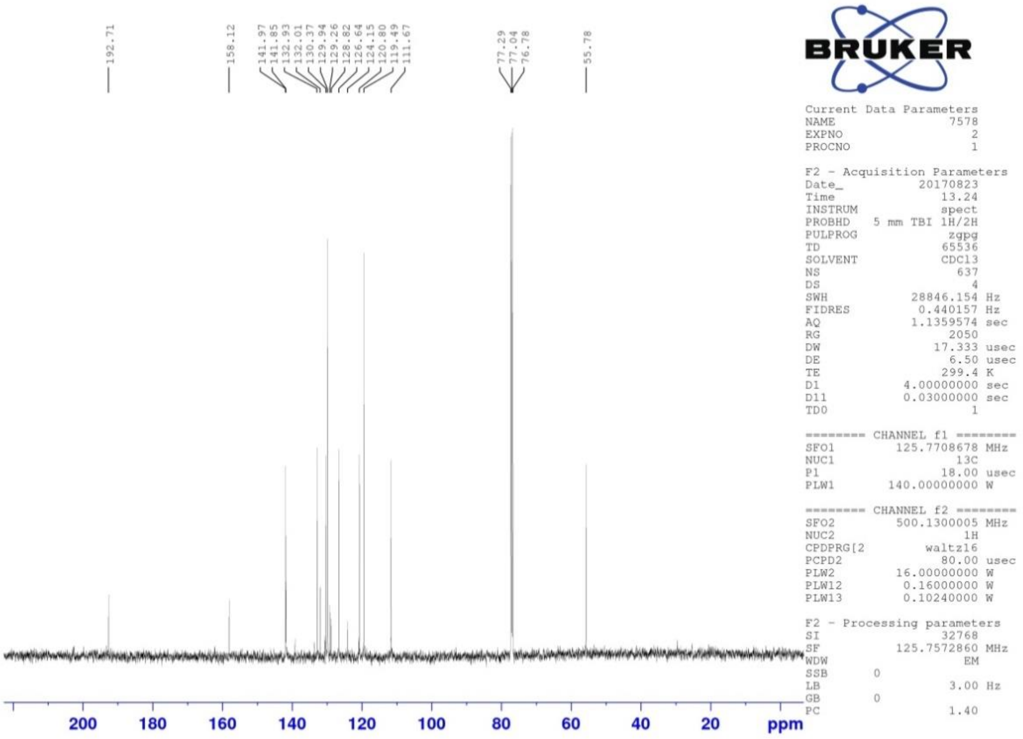

^{1}H-NMR spectra of **4b**

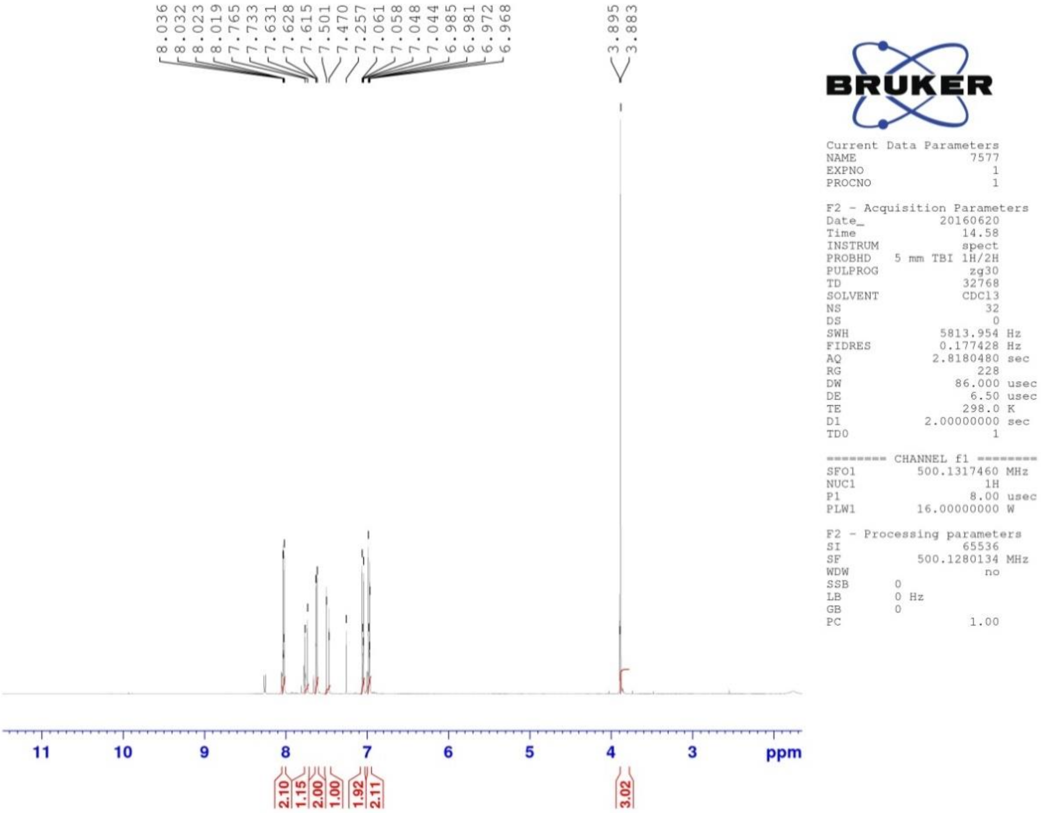

^{13}C-NMR spectra of **4b**

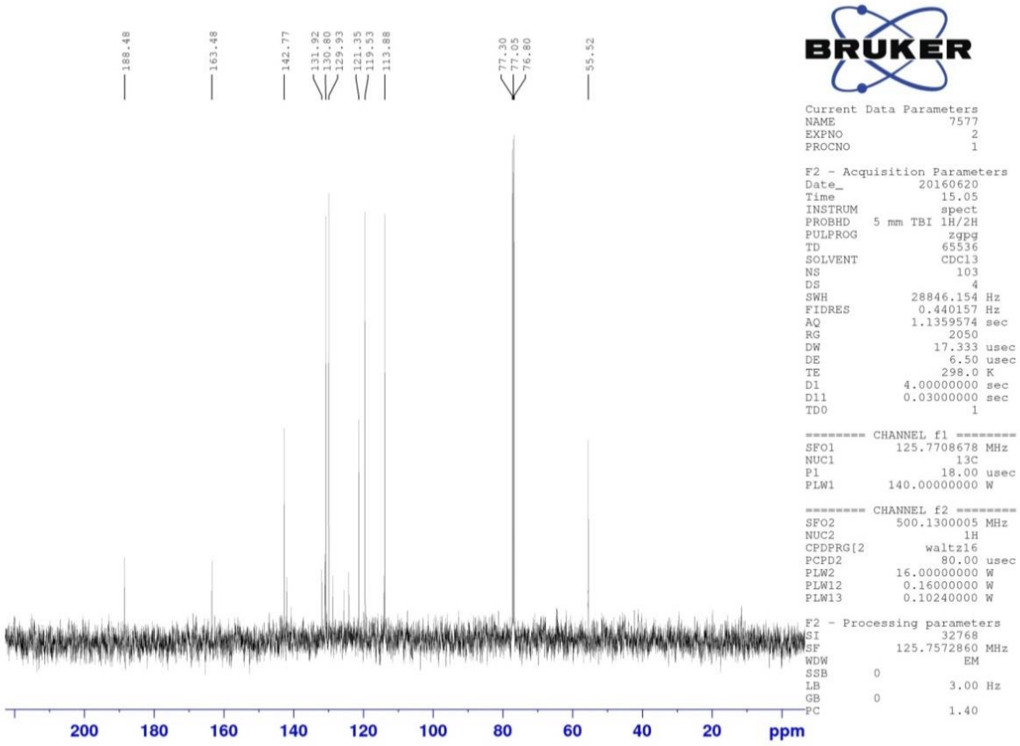

^{1}H-NMR spectra of **4c**

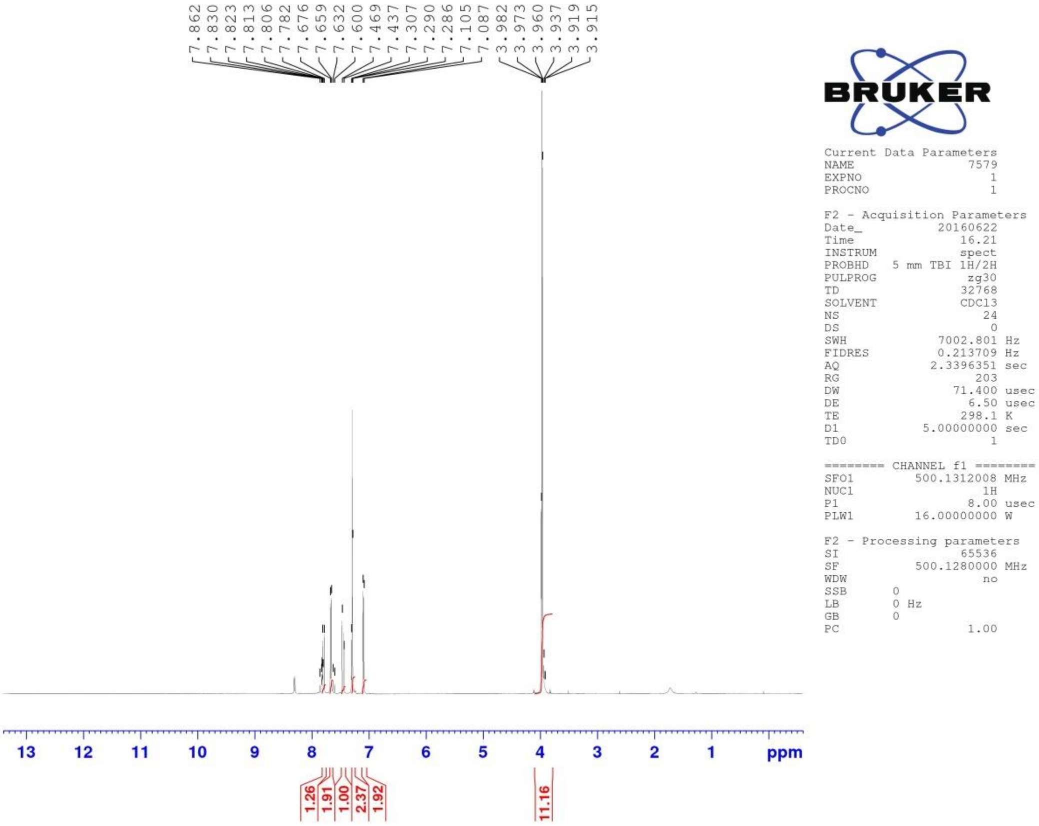

^{13}C-NMR spectra of **4c**

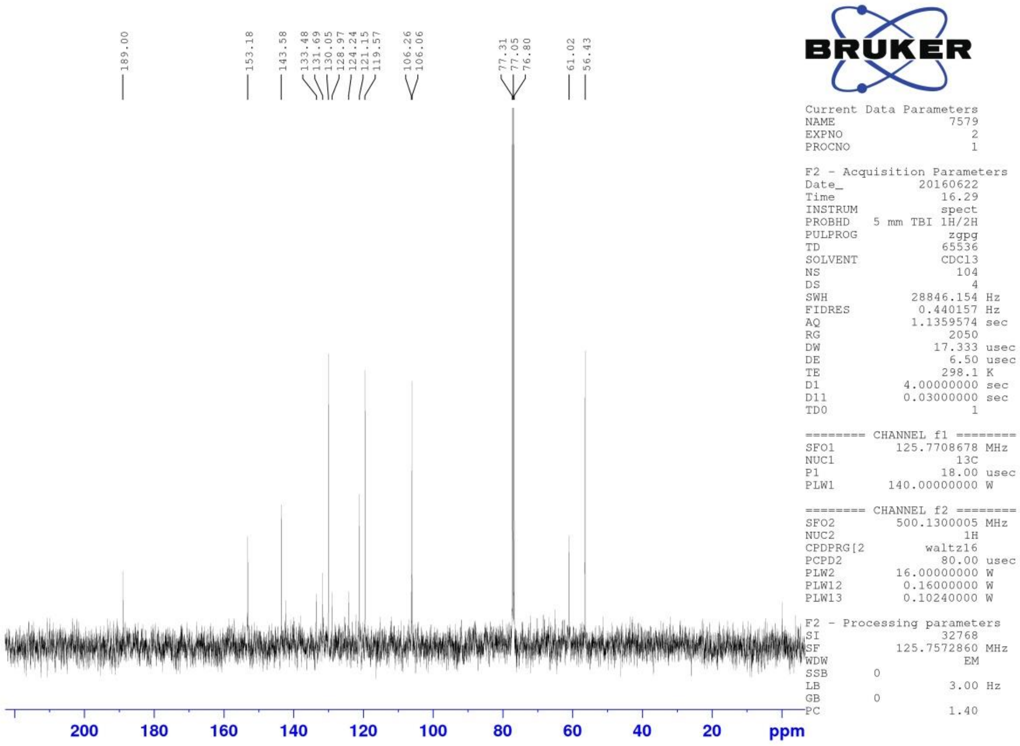

^{1}H-NMR spectra of **4d**

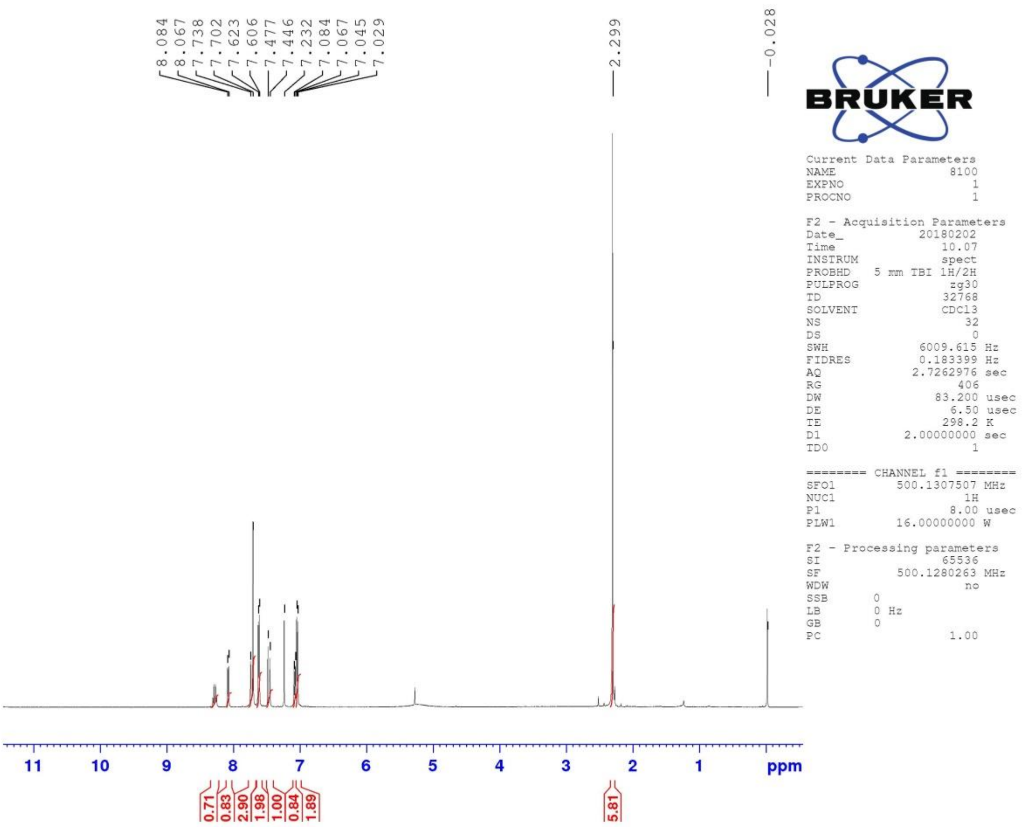

^{13}C-NMR spectra of **4d**

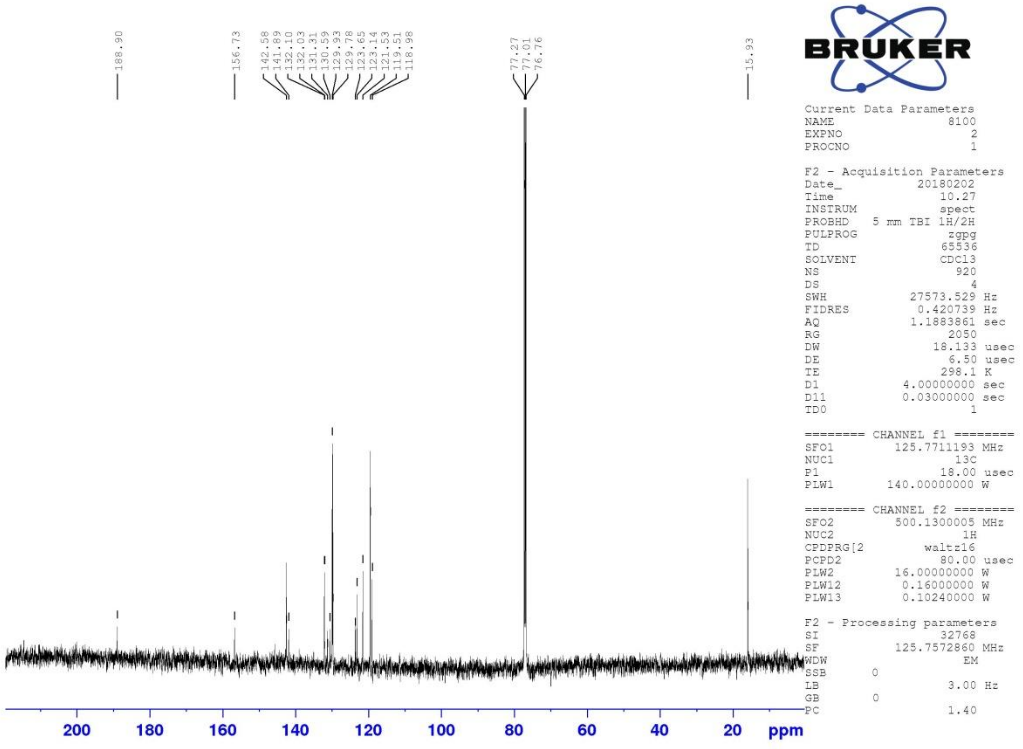

^{1}H-NMR spectra of **6a**

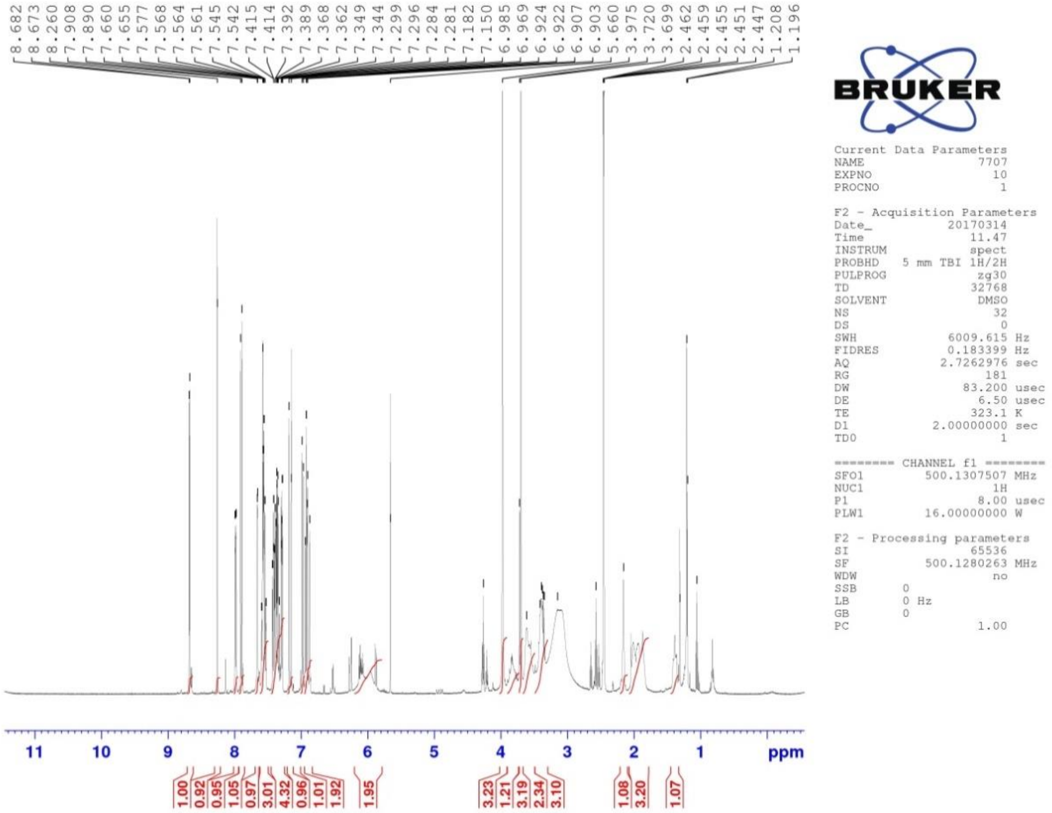

^{13}C-NMR spectra of **6a**

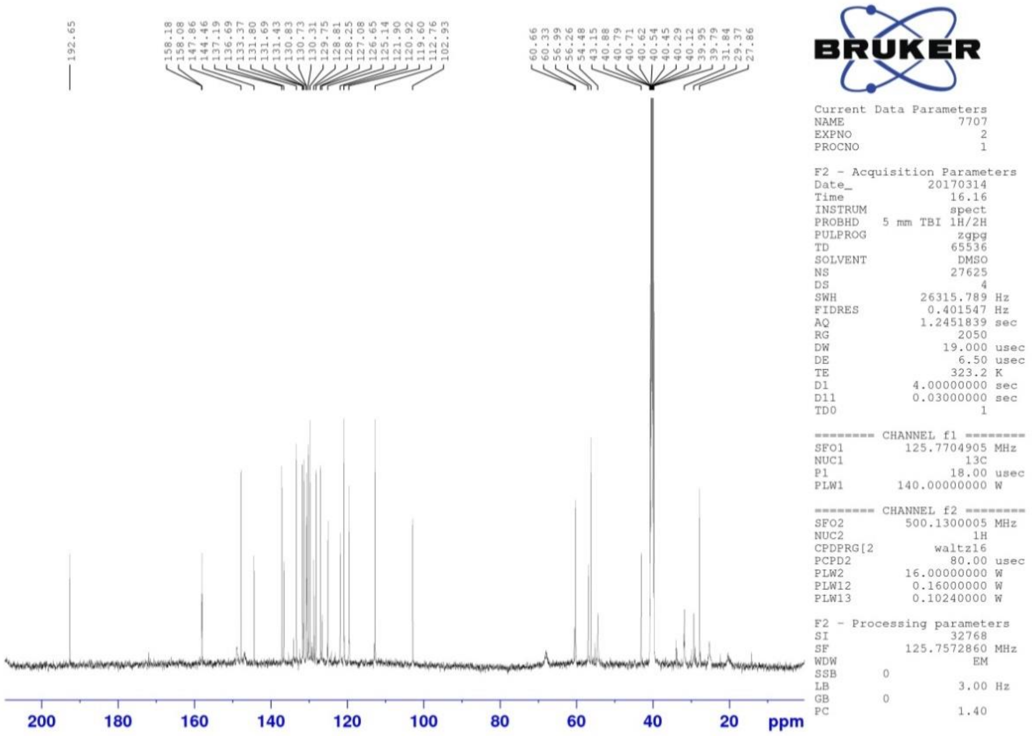

^{1}H-NMR spectra of **6b**

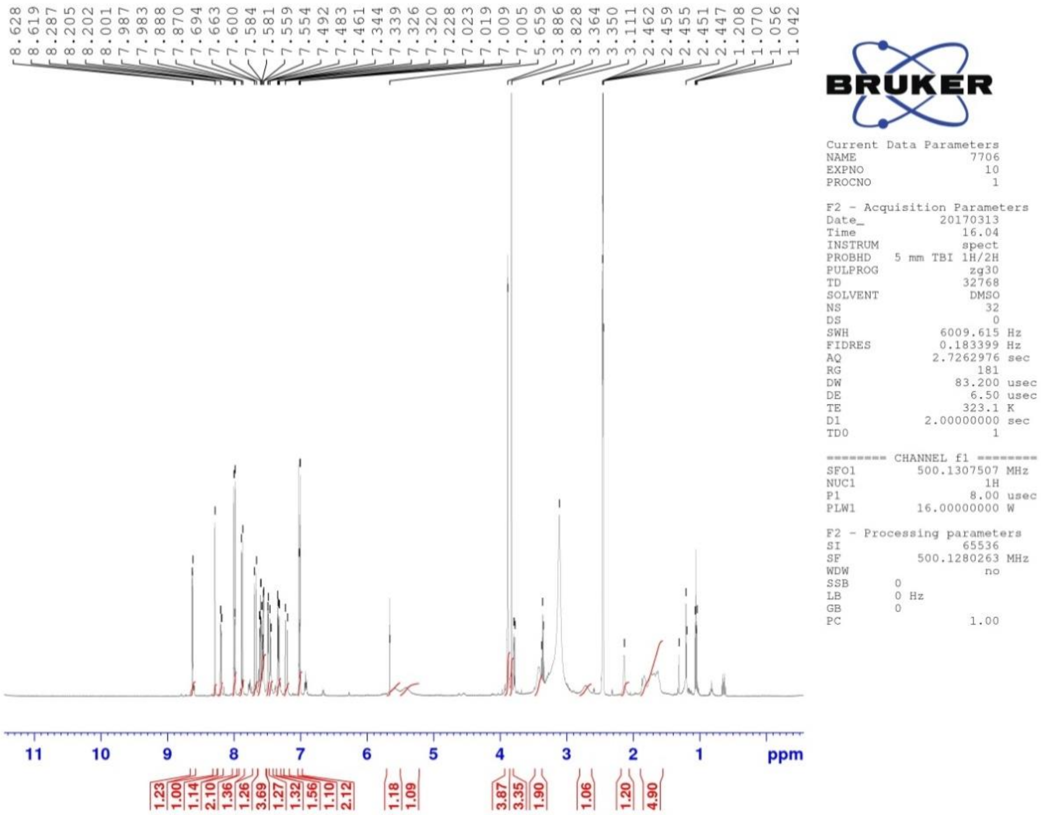

^{13}C-NMR spectra of **6b**

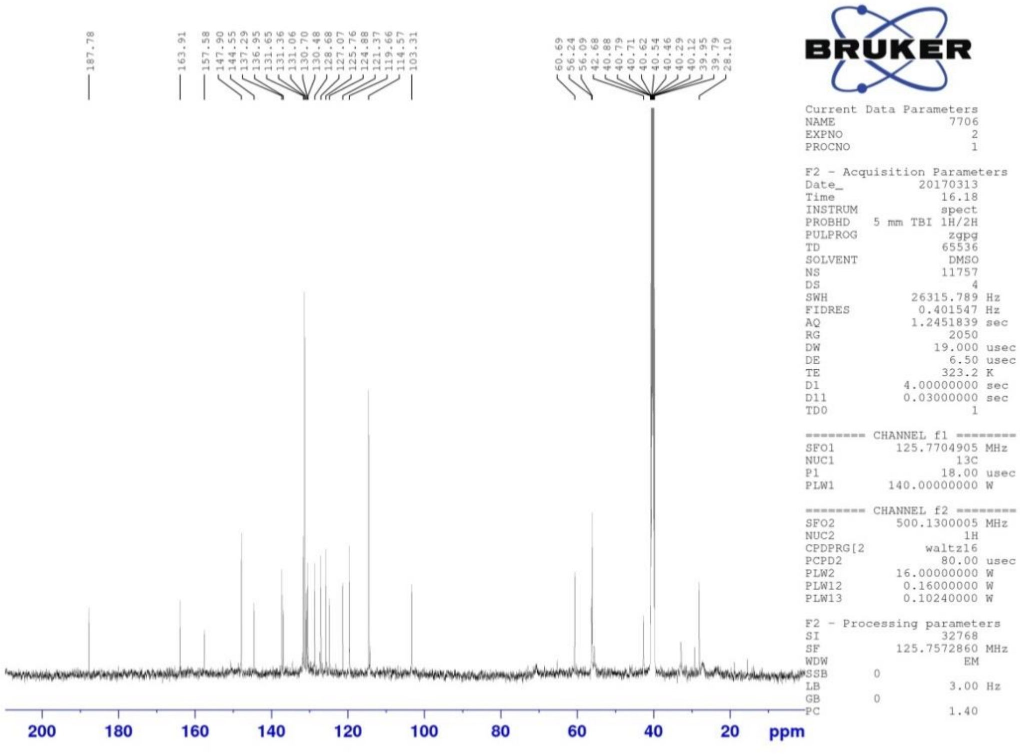

^{1}H-NMR spectra of **6c**

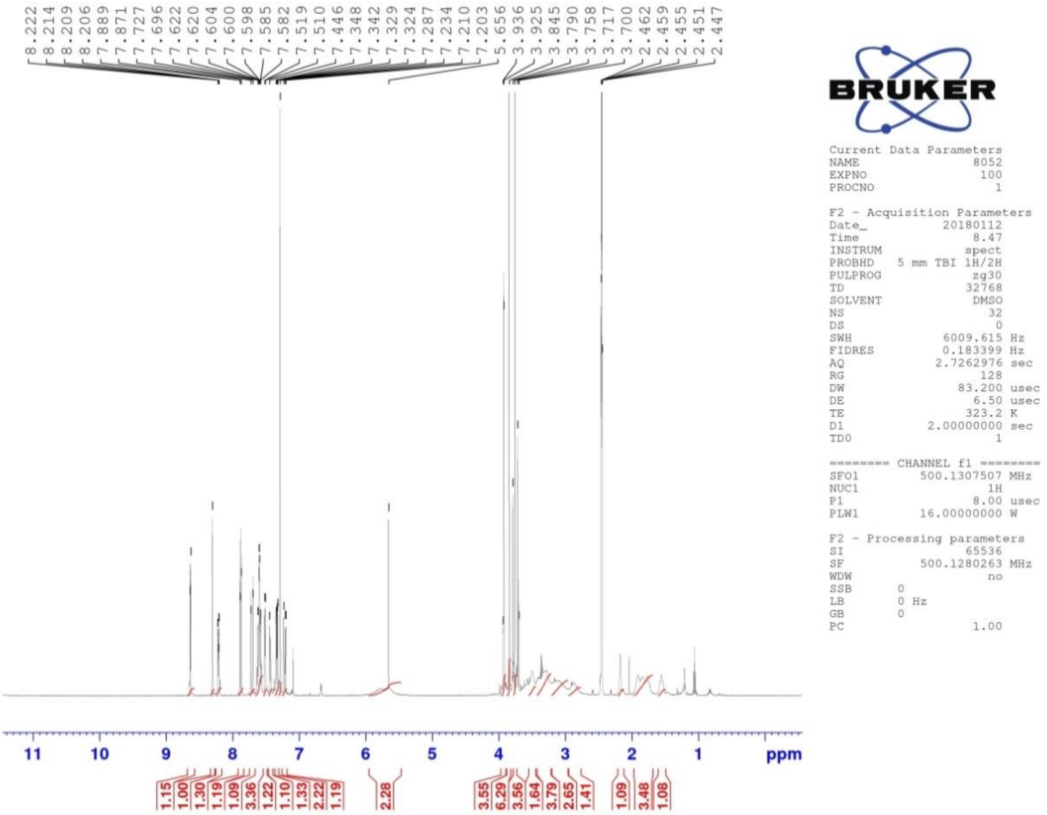

^{13}C-NMR spectra of **6c**

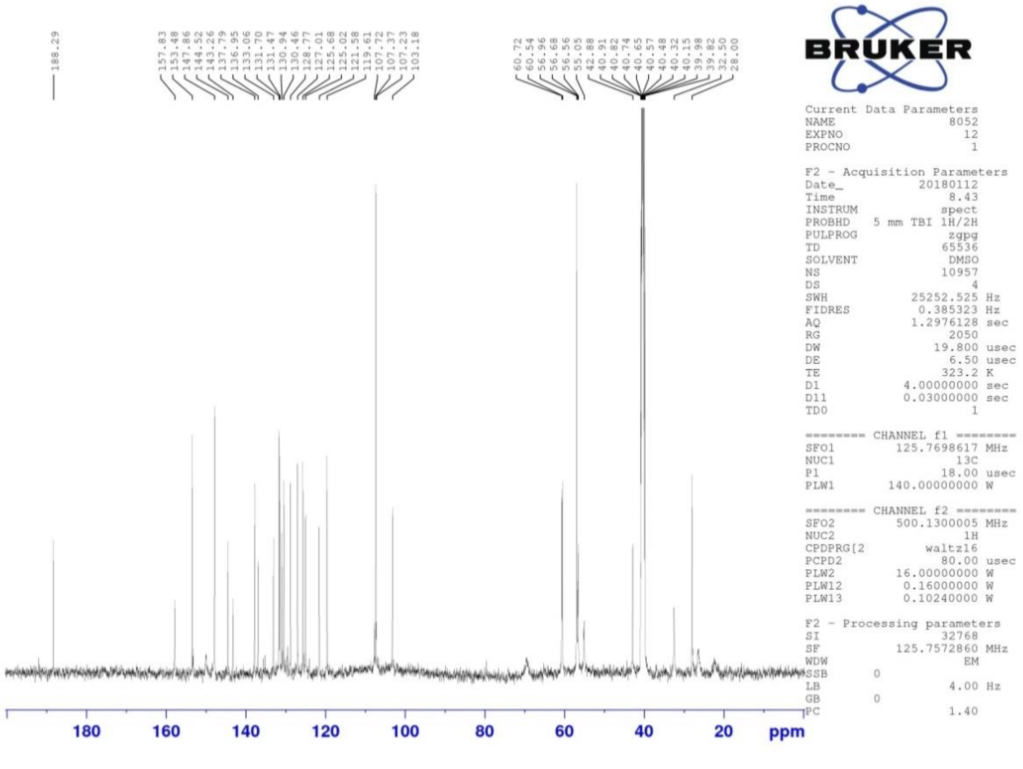

^{1}H-NMR spectra of **6d**

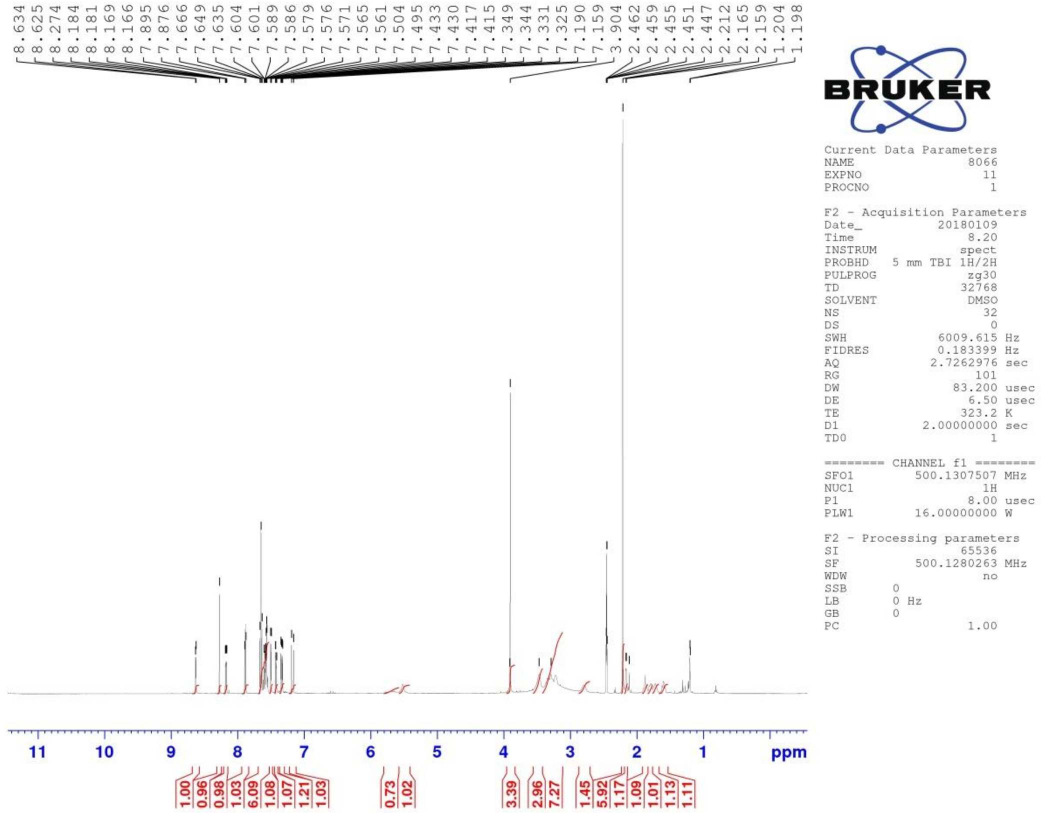

^{13}C-NMR spectra of **6d**

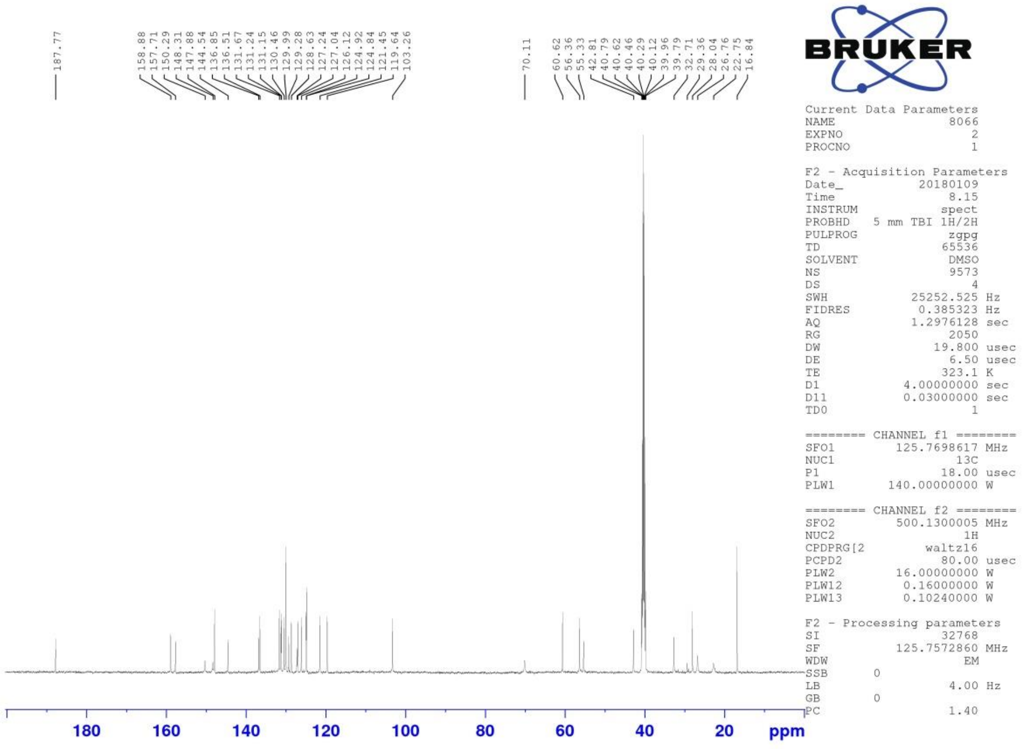

^{1}H-^{13}C-HSQC spectra of **6d**

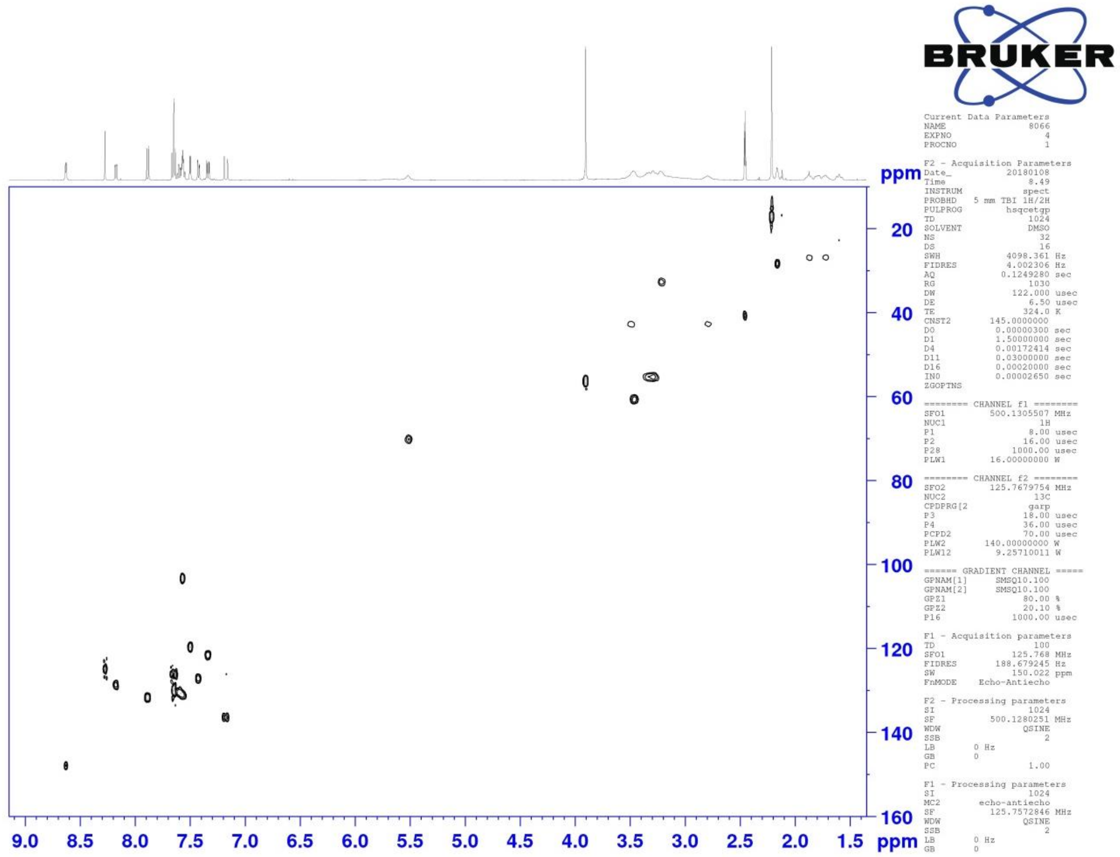

^{1}H-^{13}C-HMBC spectra of **6d**

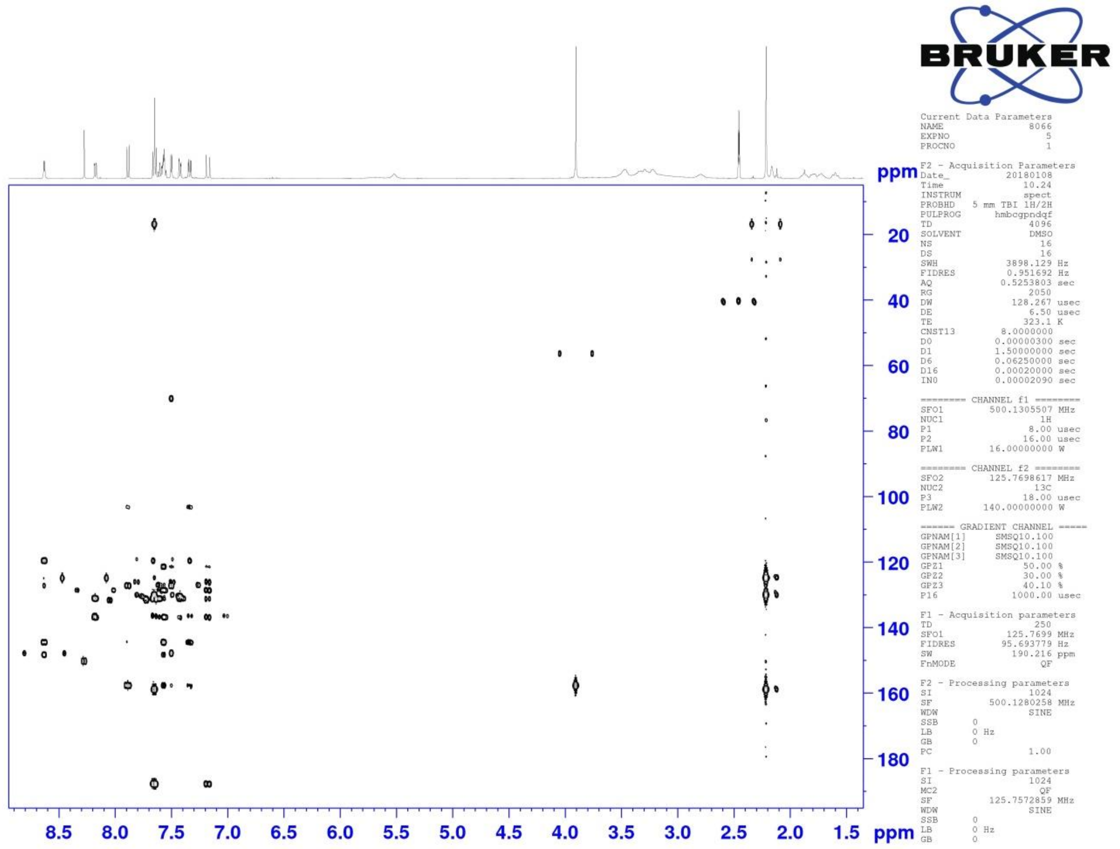

^{1}H-^{1}H-NOESY spectra of **6d**

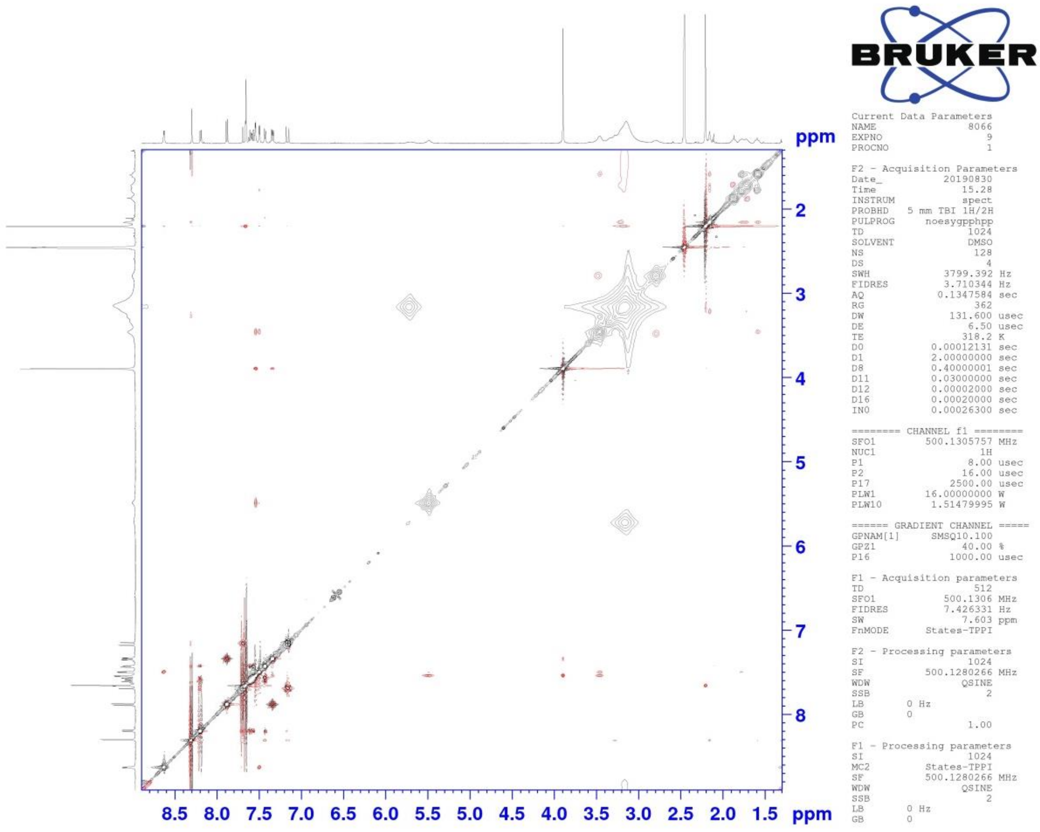

^{1}H-NMR spectra of **6*d**

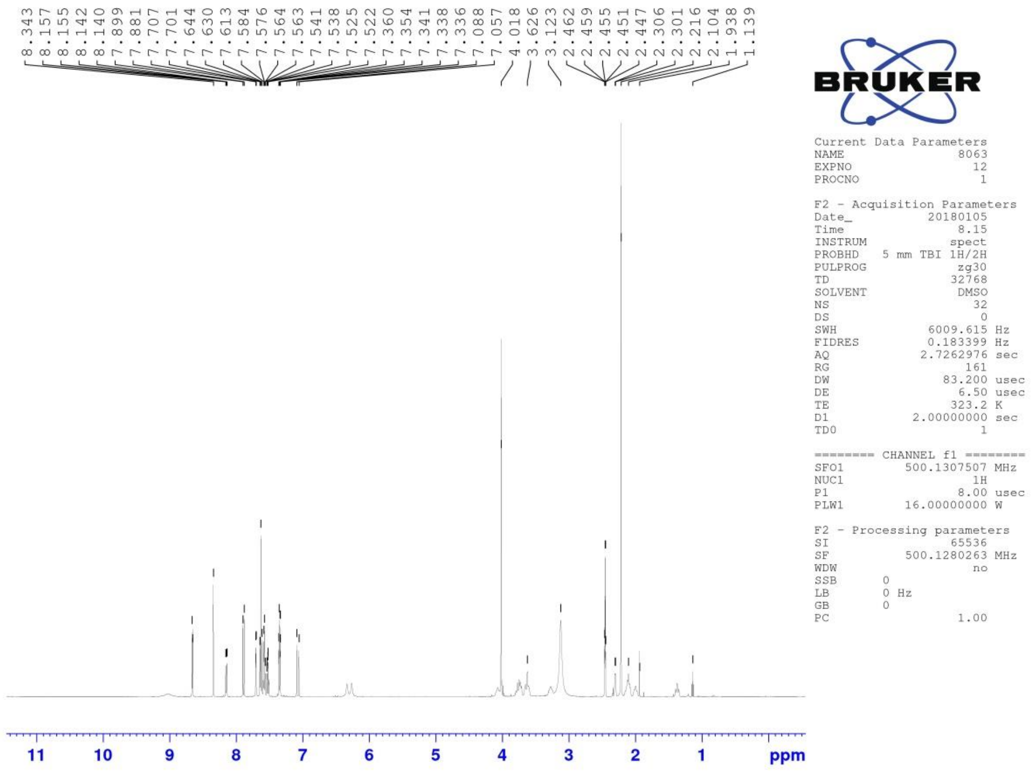

^{13}C-NMR spectra of **6*d**

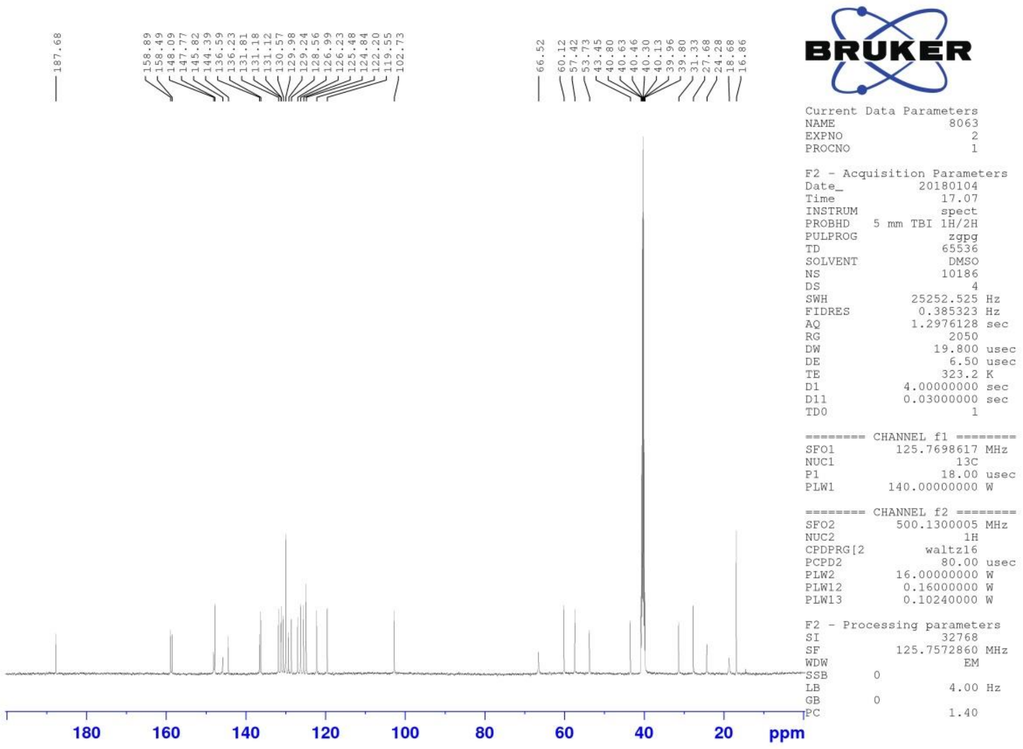

^{1}H-^{13}C-HSQC spectra of **6d***

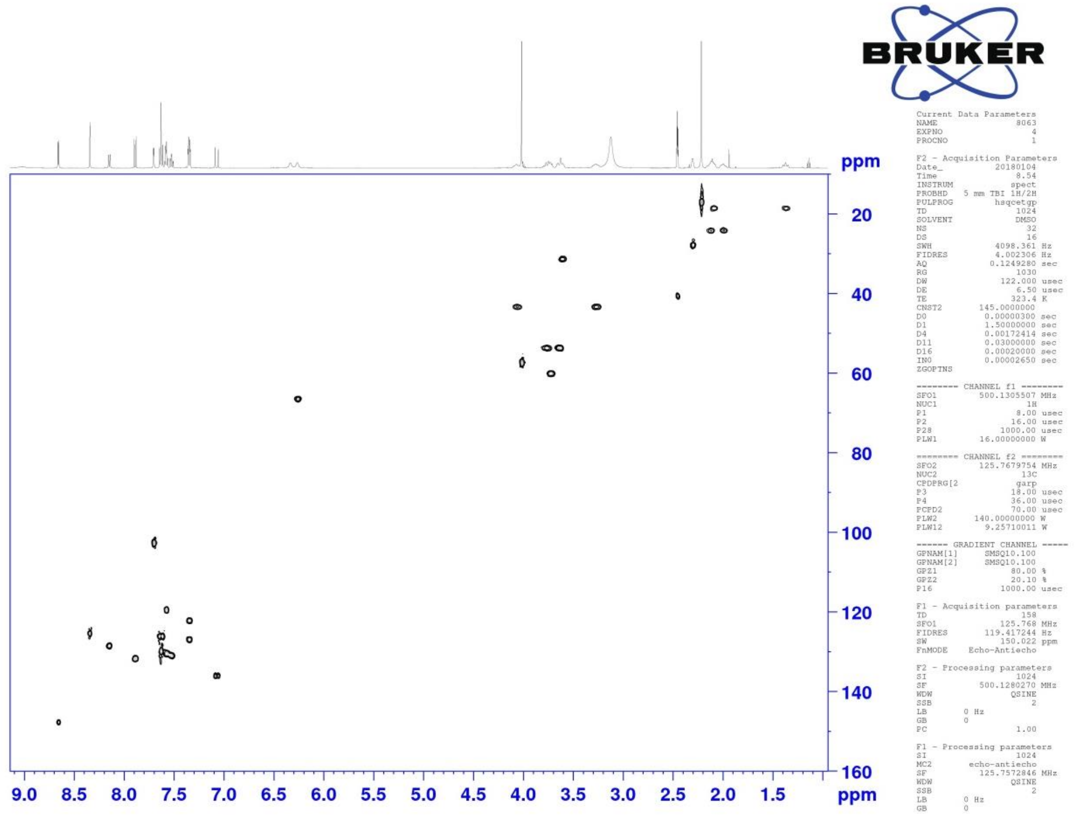

^{1}H-^{13}C-HMBC spectra of **6d***

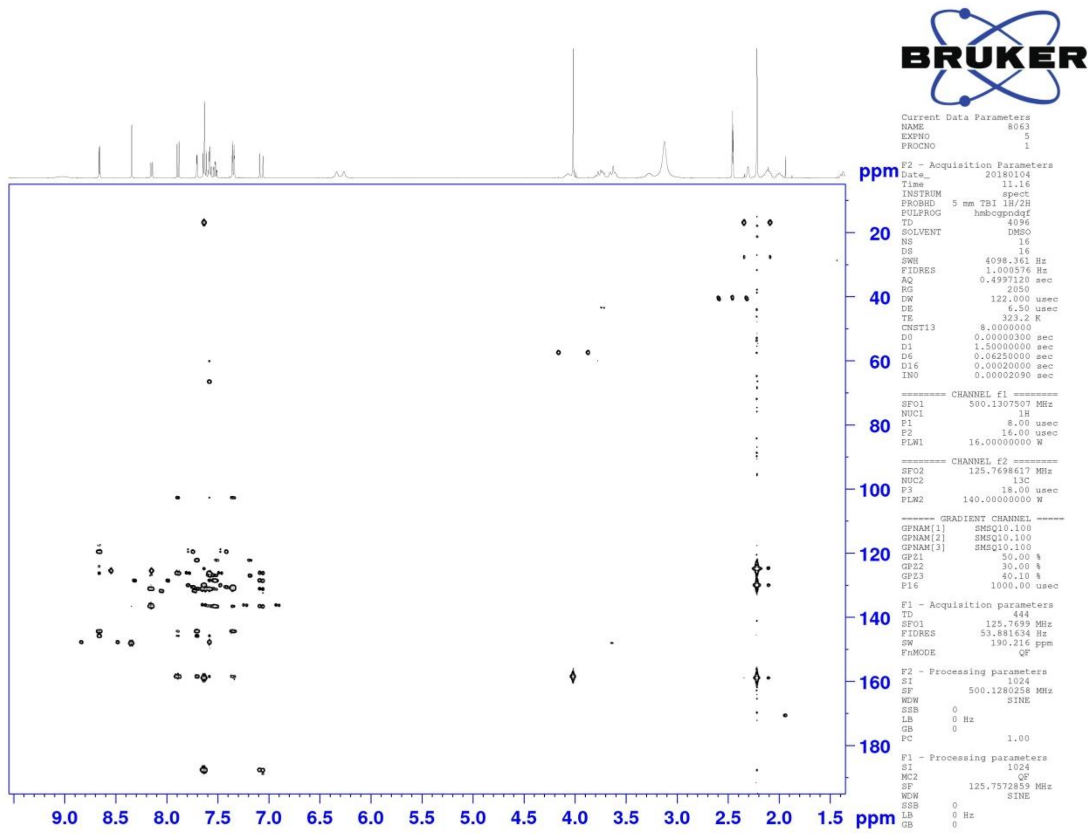

^{1}H-^{1}H-NOESY spectra of **6d***

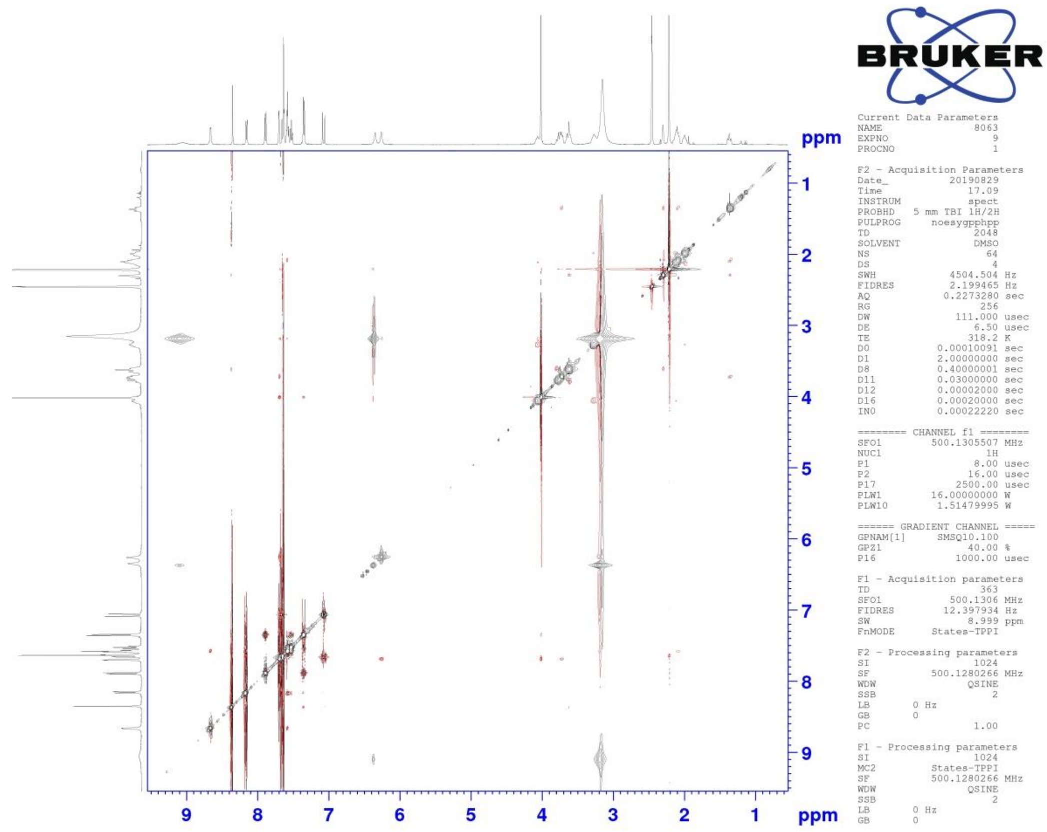

^{1}H-NMR spectra of **7a**

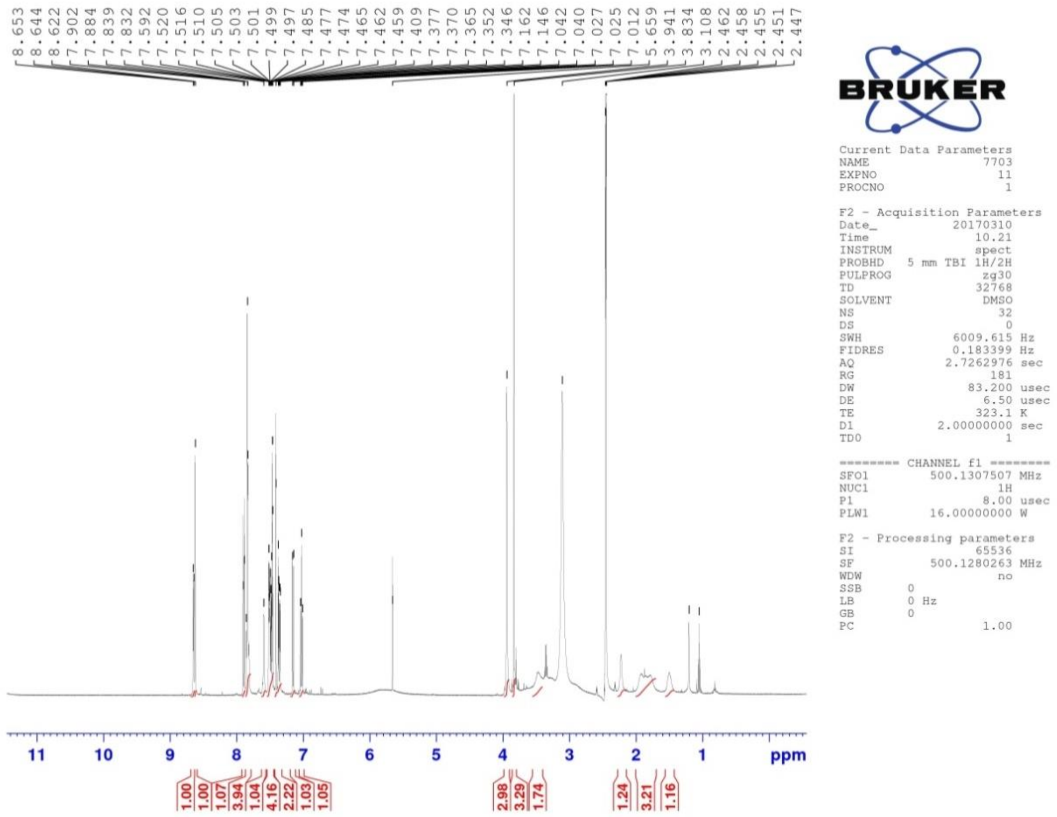

^{13}C-NMR spectra of **7a**

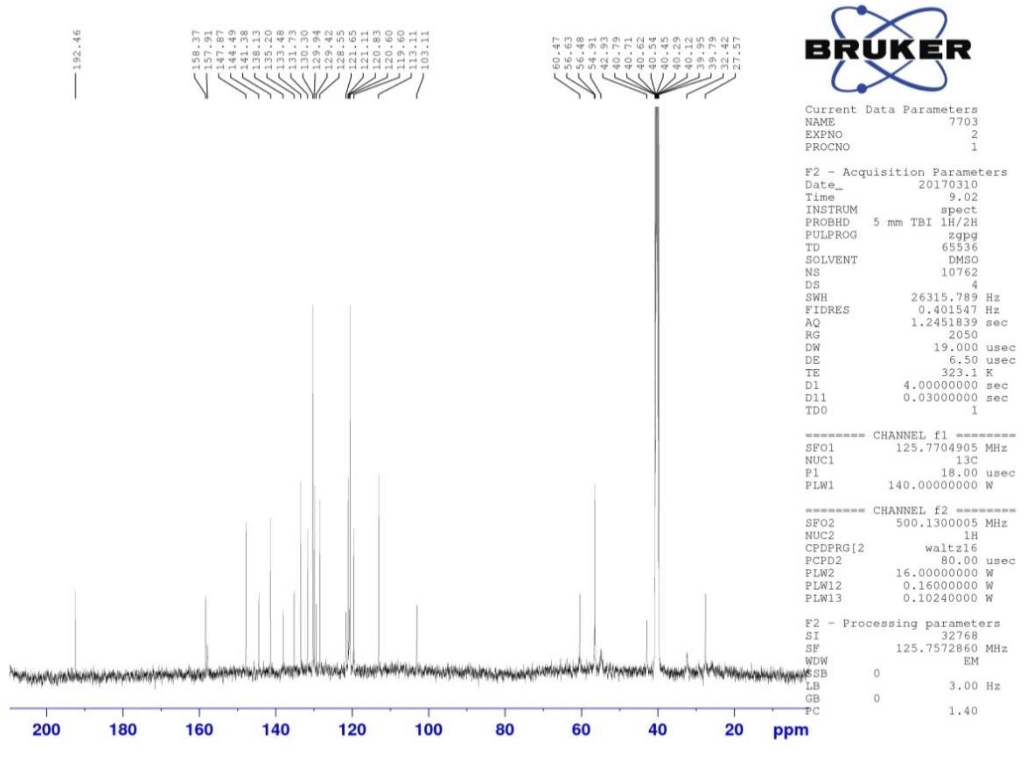

^{1}H-NMR spectra of **7b**

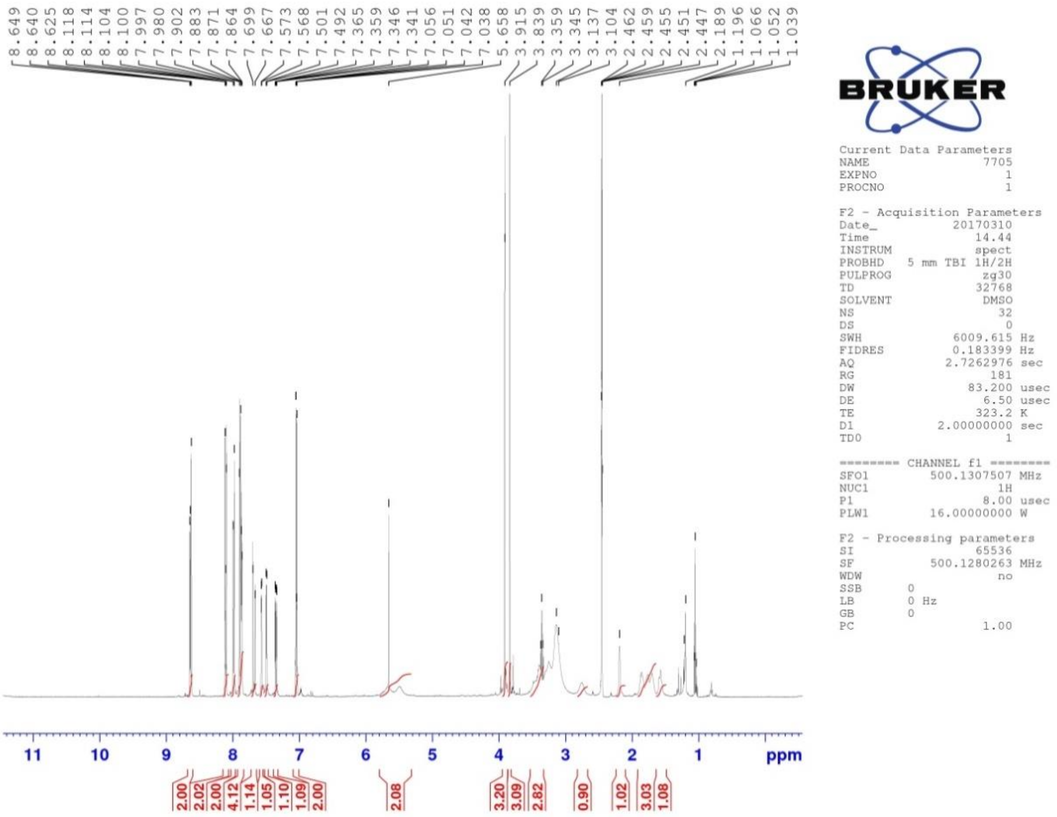

^{13}C-NMR spectra of **7b**

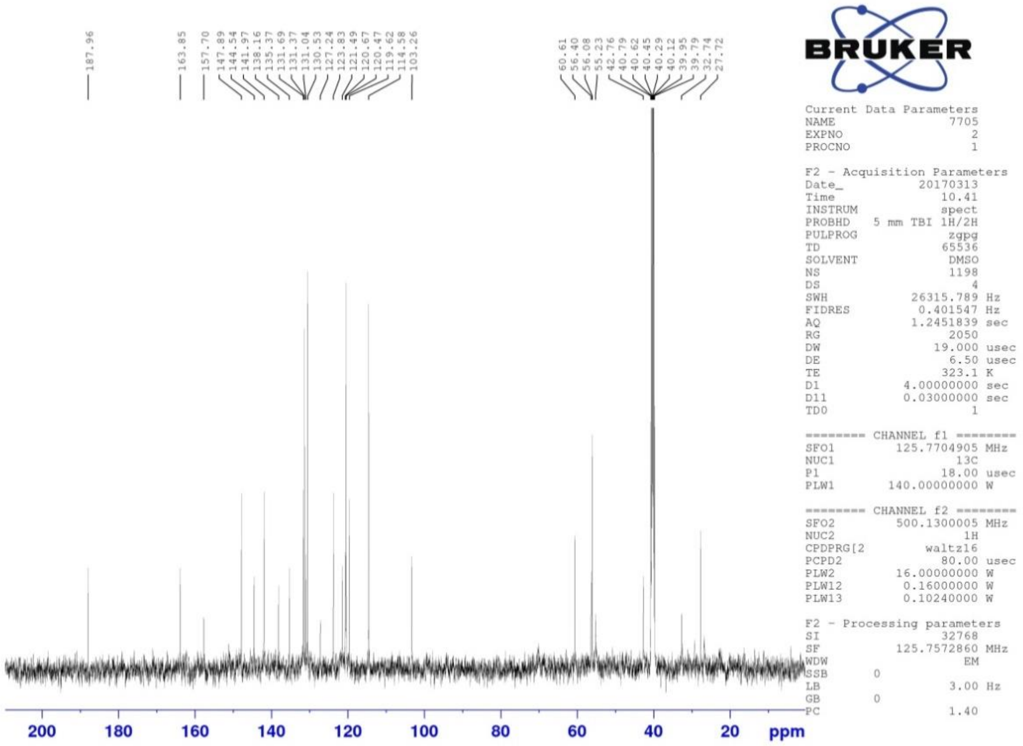

^{1}H-NMR spectra of **7c**

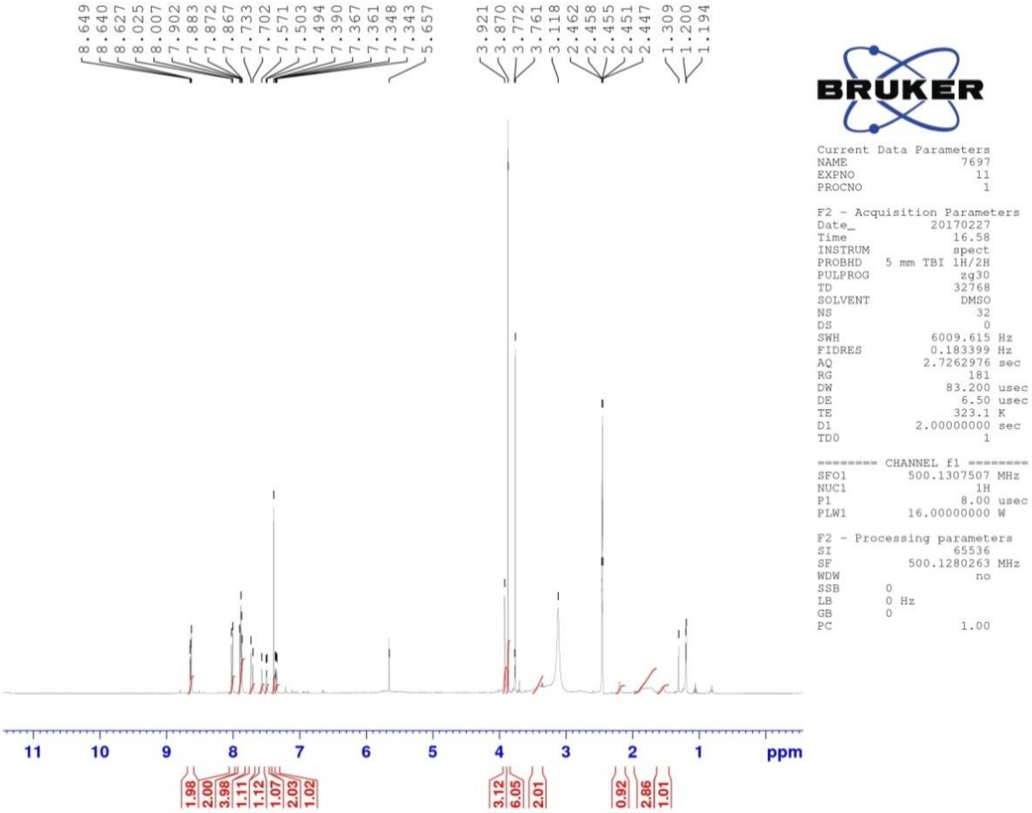

^{13}C-NMR spectra of **7c**

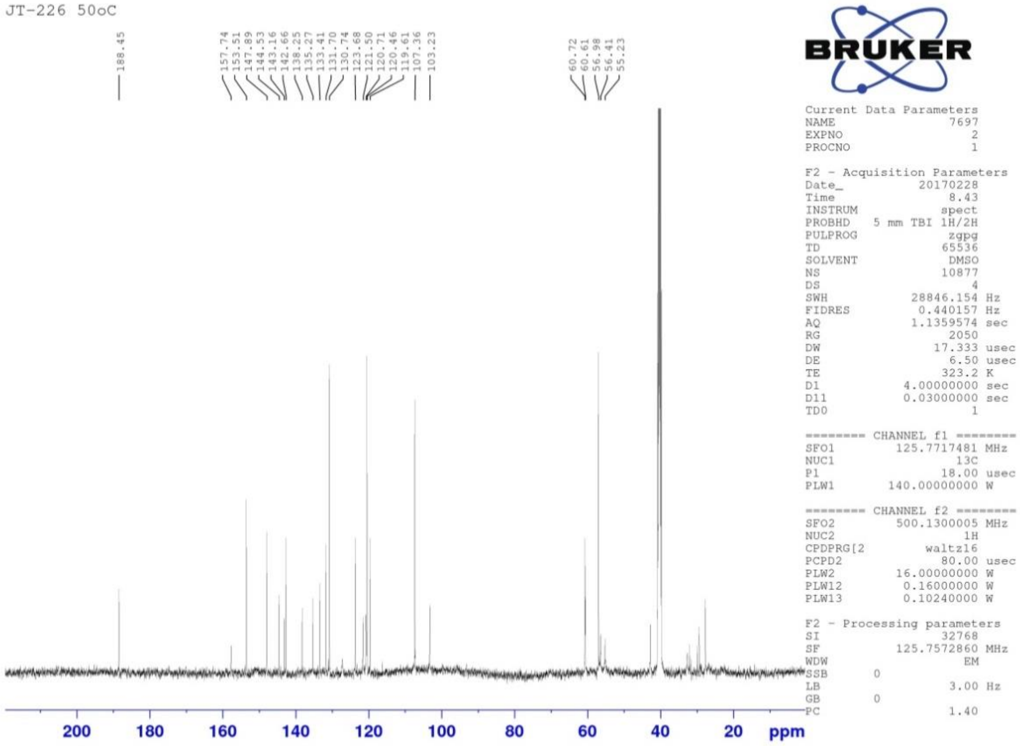

^{1}H-NMR spectra of **7d**

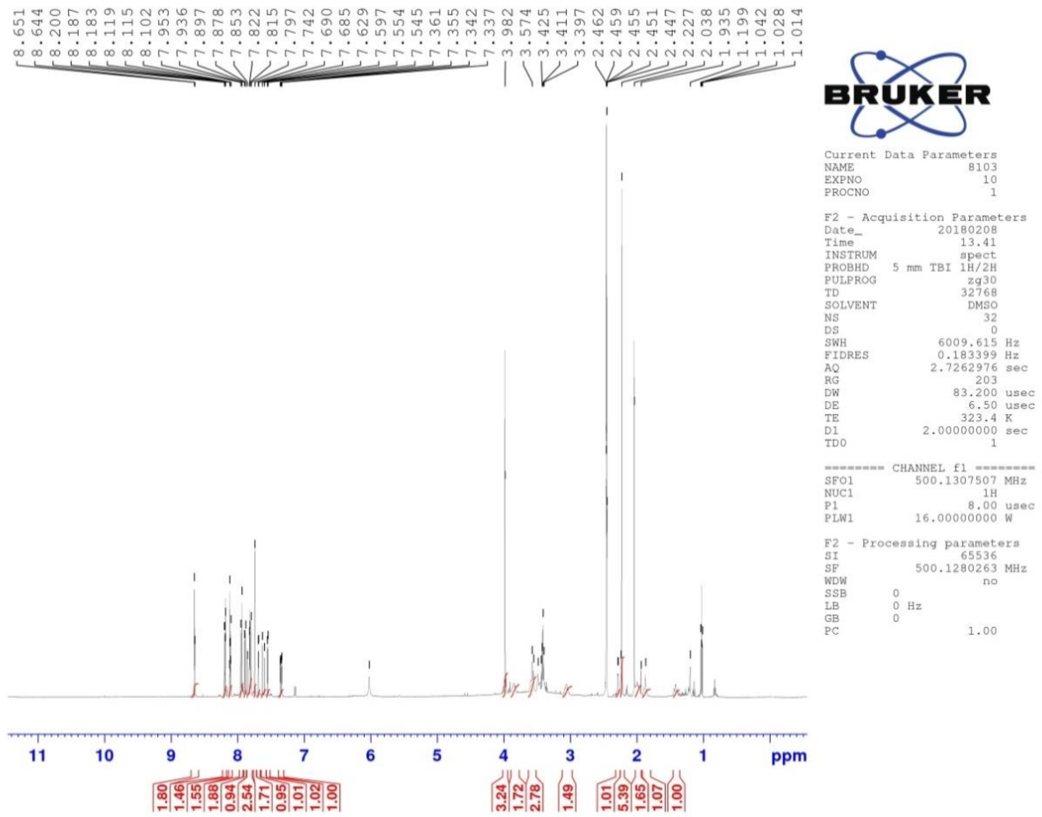

^{13}C-NMR spectra of **7d**

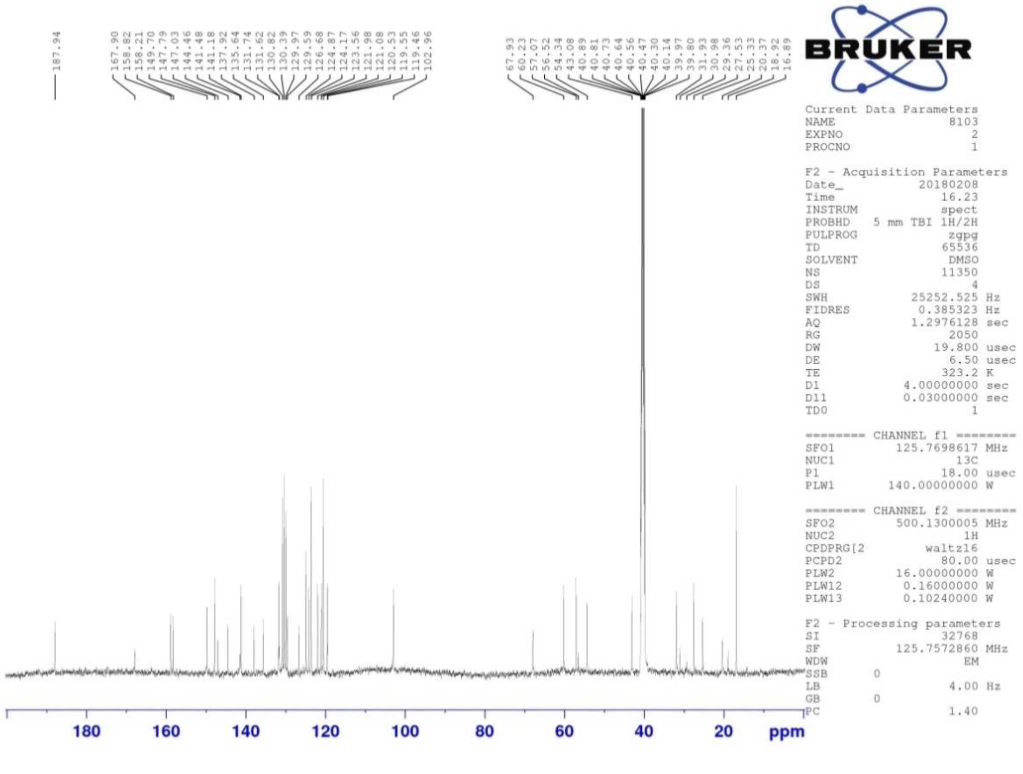

^{1}H-NMR spectra of **9a**

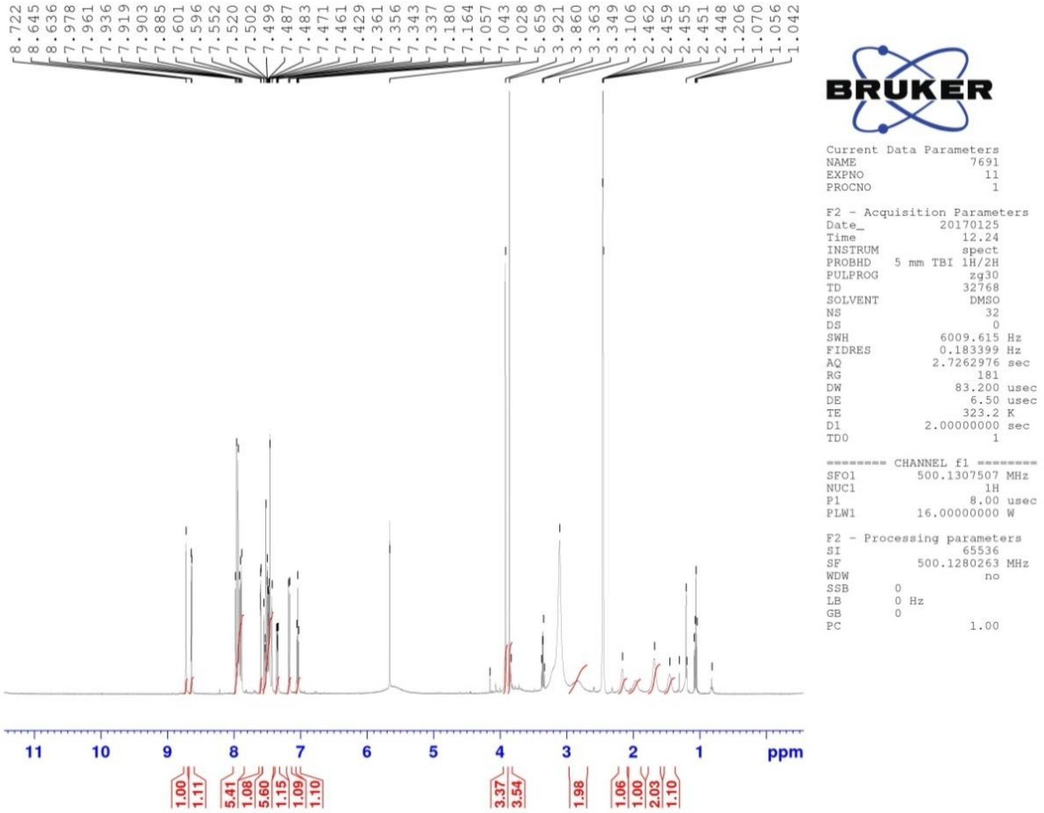

^{13}C-NMR spectra of **9a**

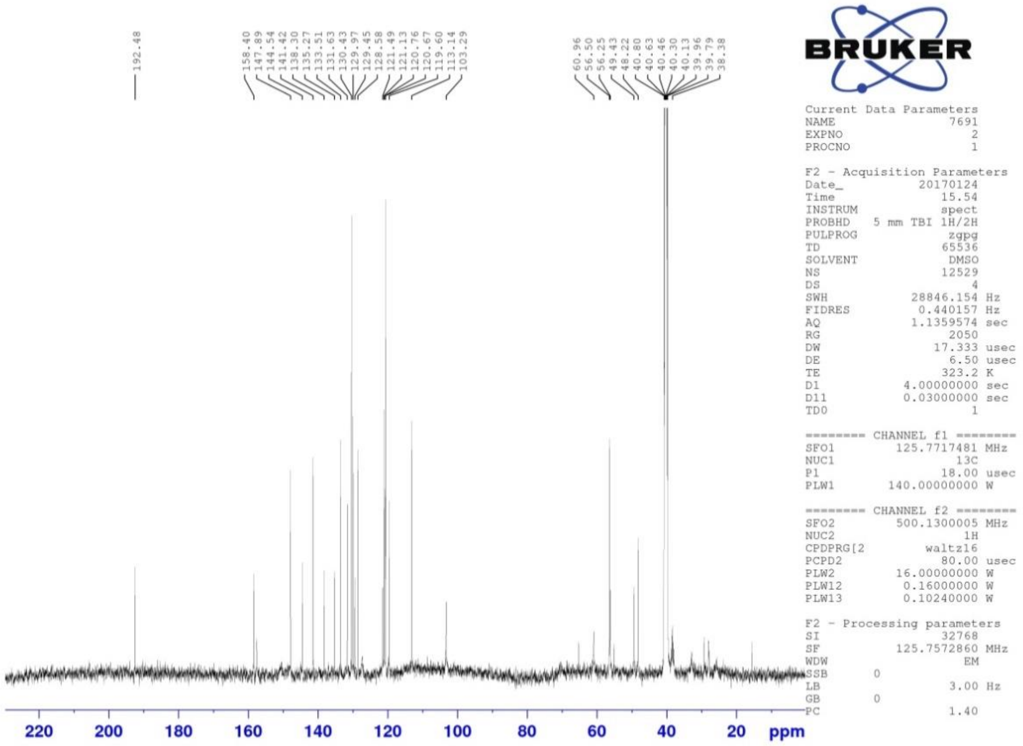

^{1}H-NMR spectra of **9b**

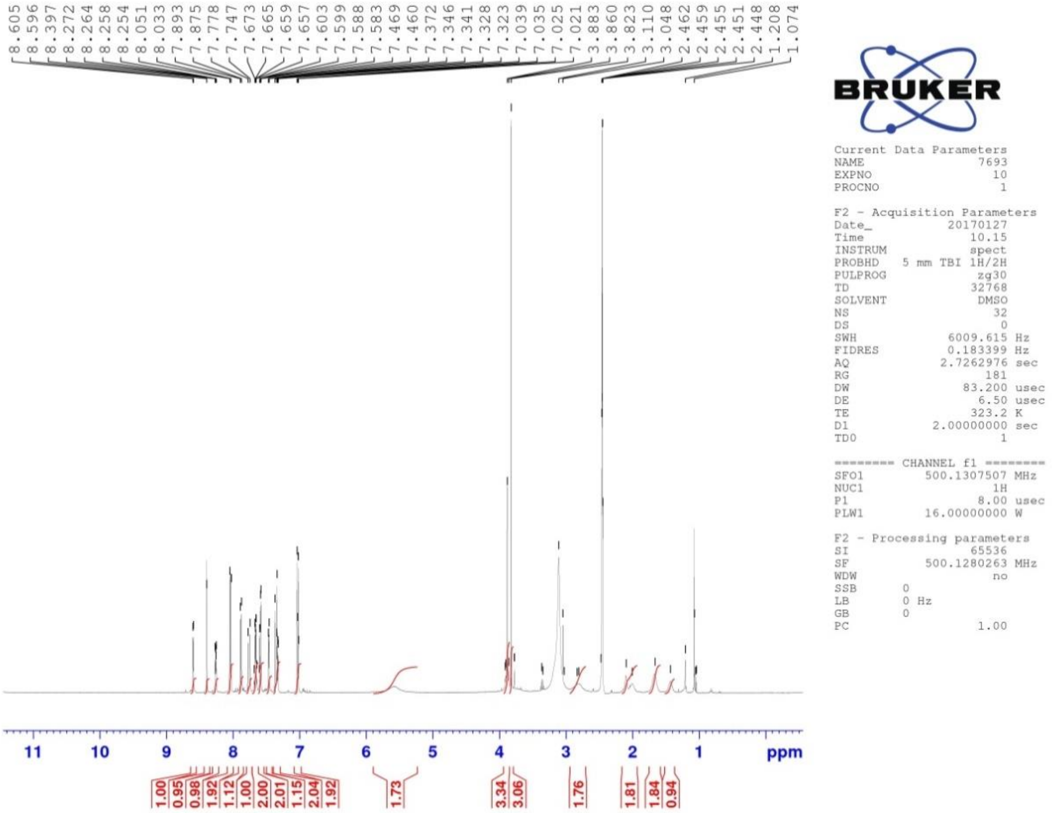

^{13}C-NMR spectra of **9b**

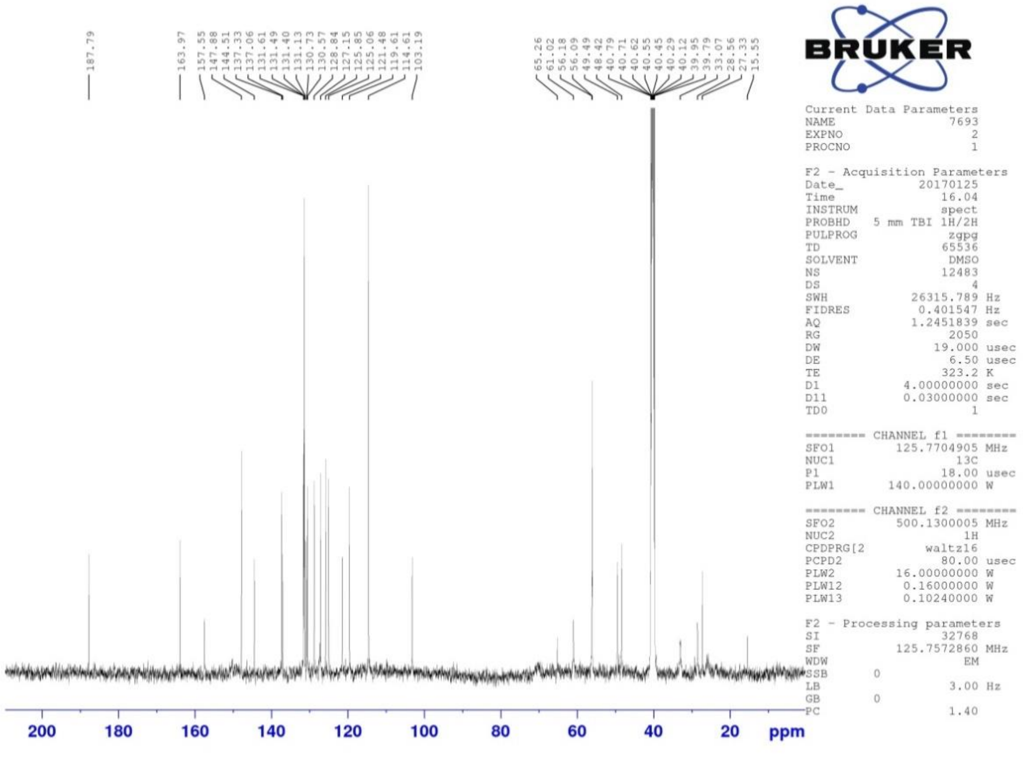

^{1}H-NMR spectra of **9c**

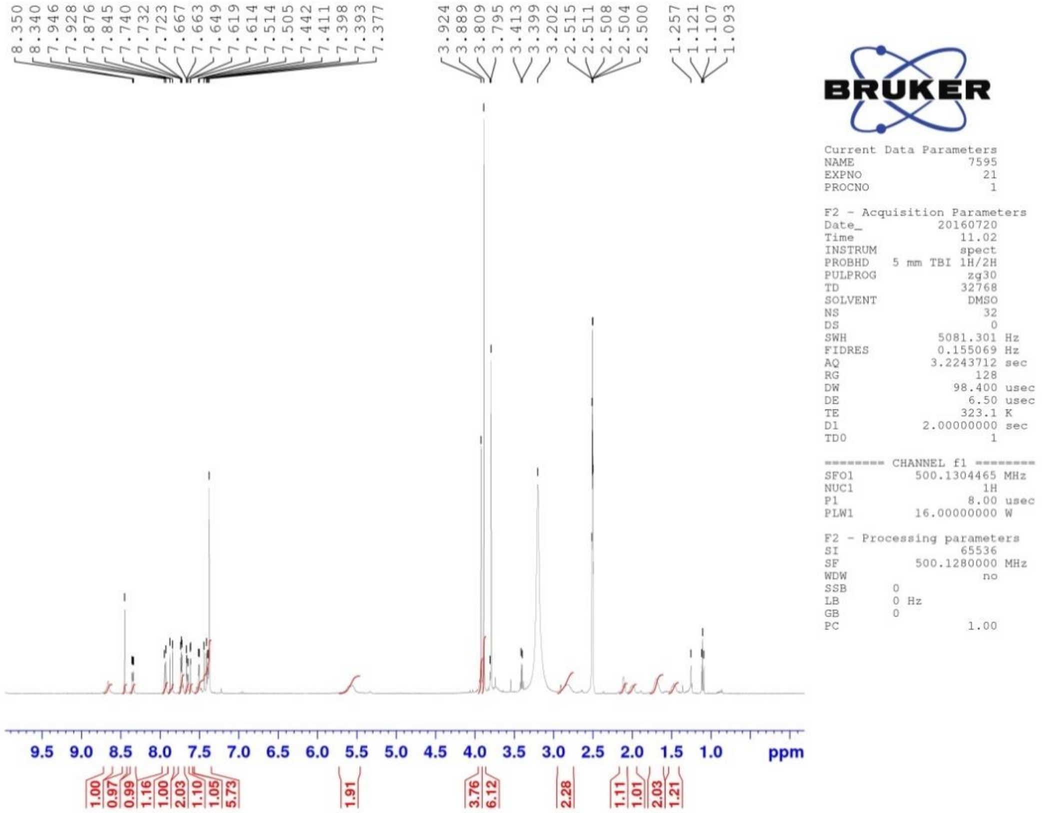

^{13}C-NMR spectra of **9c**

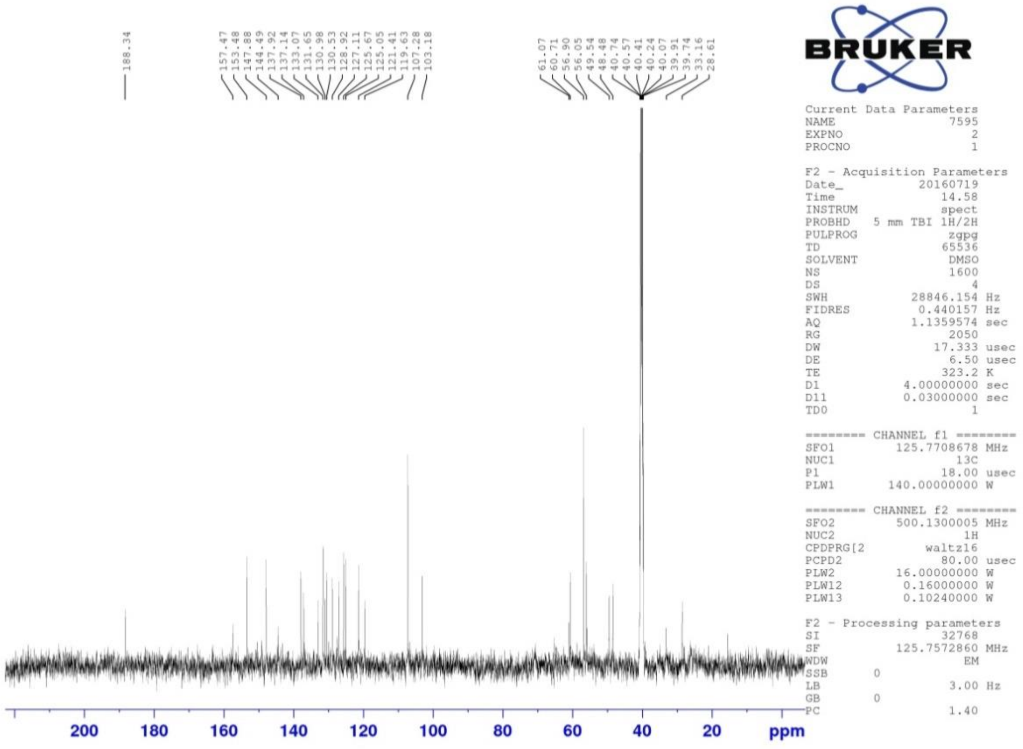

^{1}H-NMR spectra of **9*c**

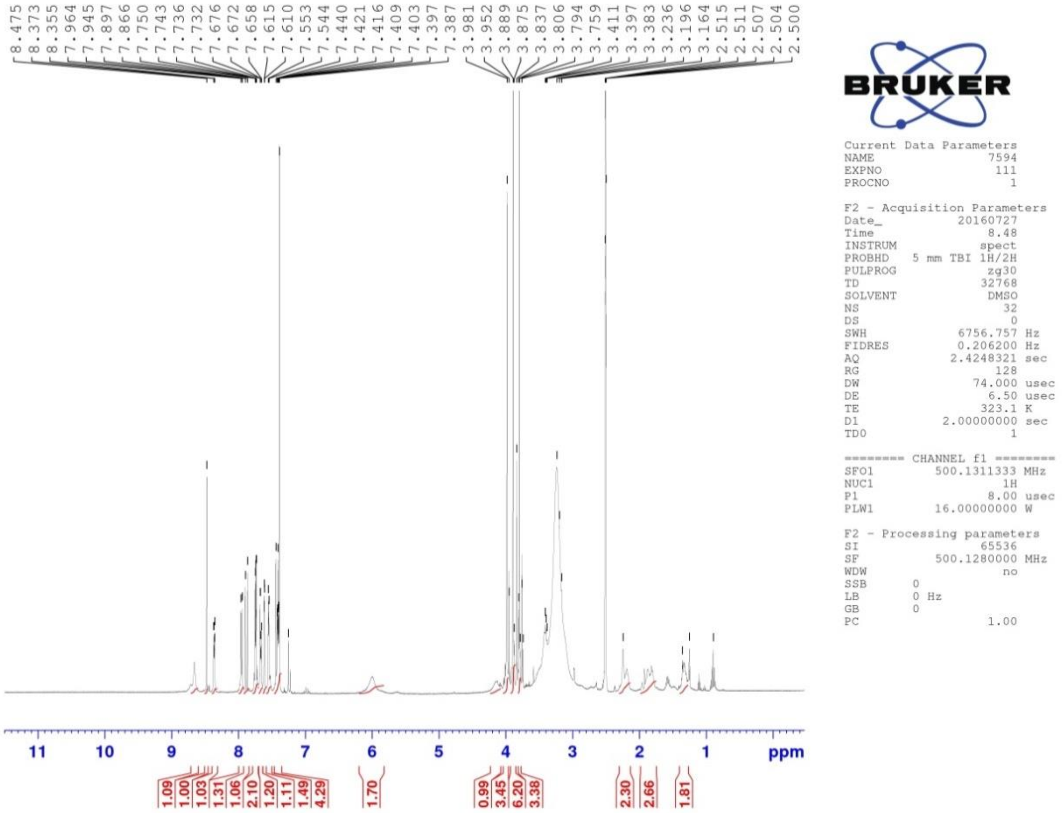

^{13}C-NMR spectra of **9*c**

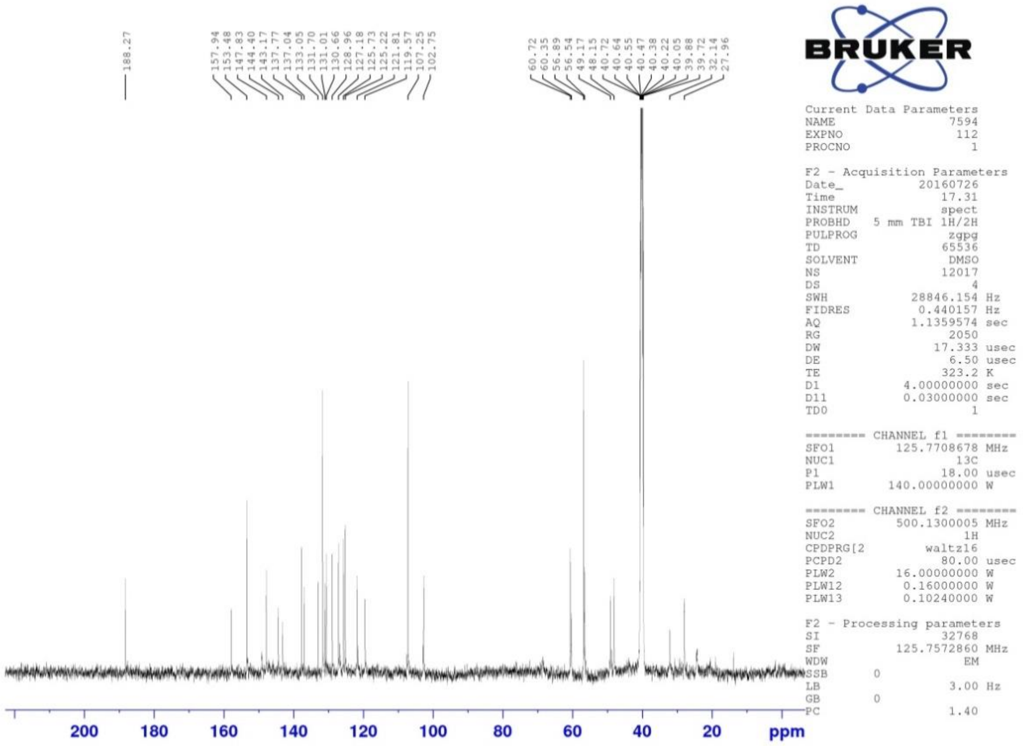

^{1}H-NMR spectra of **9d**

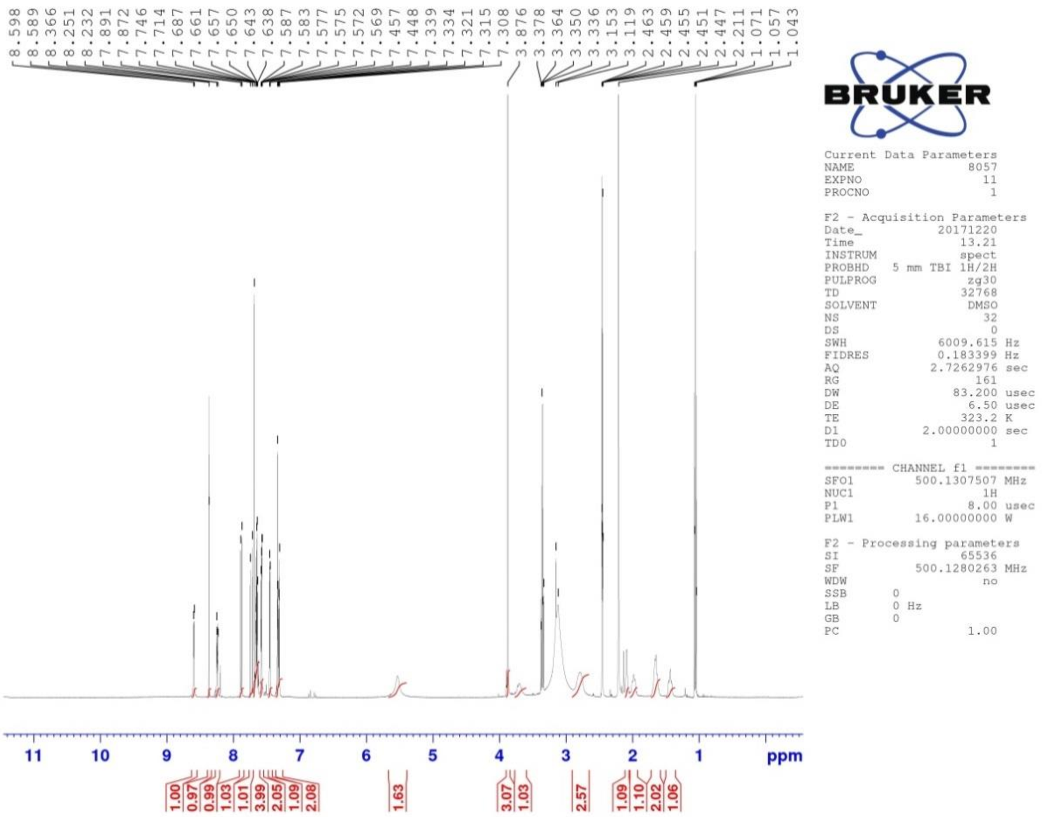

^{13}C-NMR spectra of **9d**

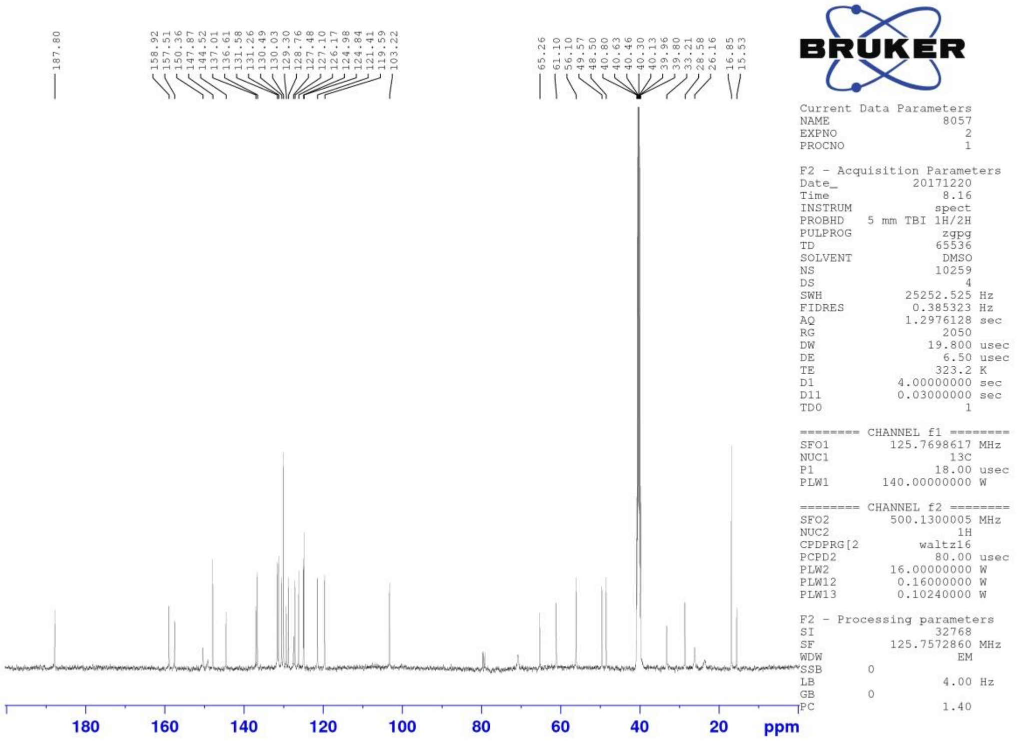

^{1}H-NMR spectra of **9*d**

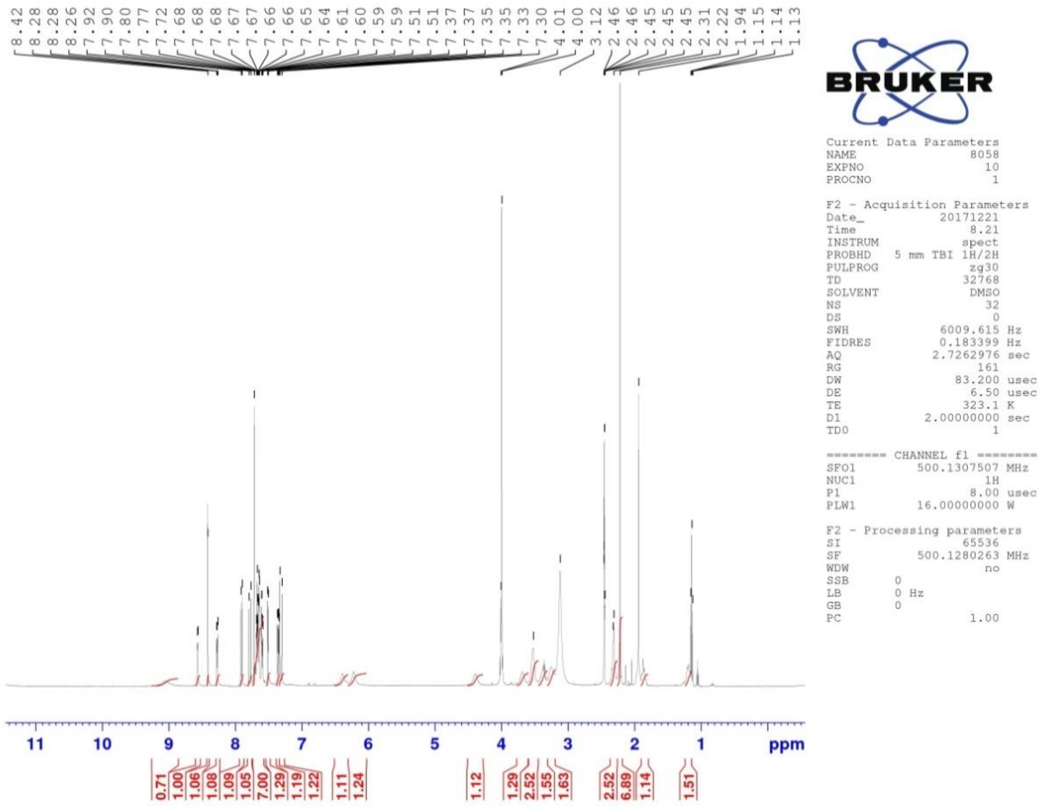

^{13}C-NMR spectra of **9*d**

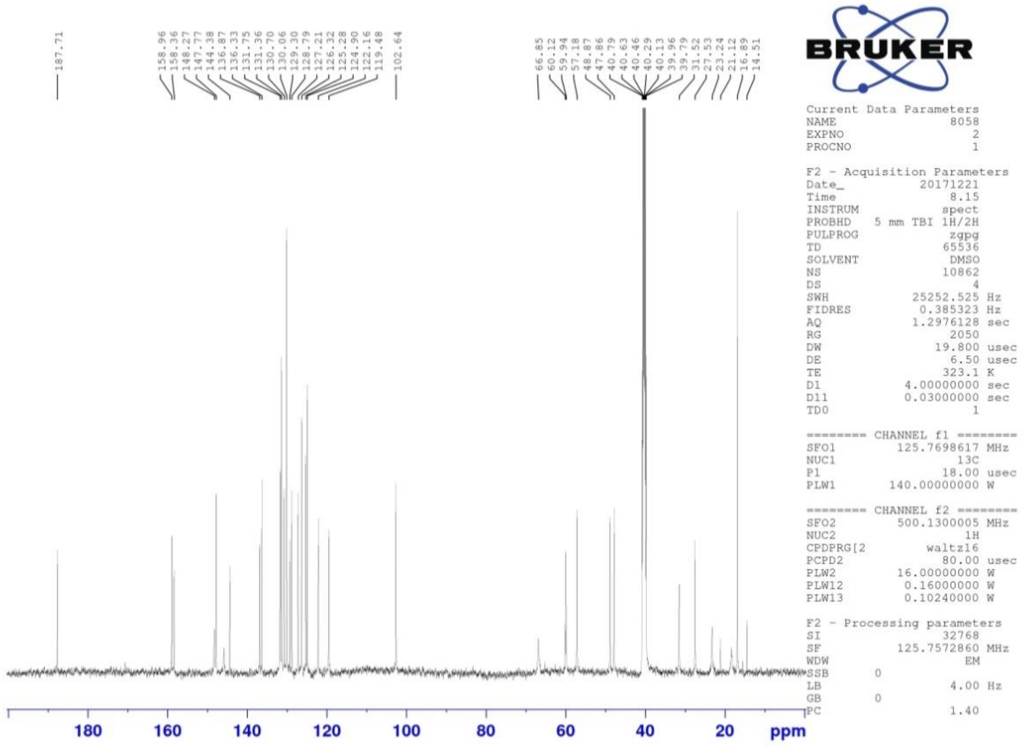

^{1}H-NMR spectra of **10a**

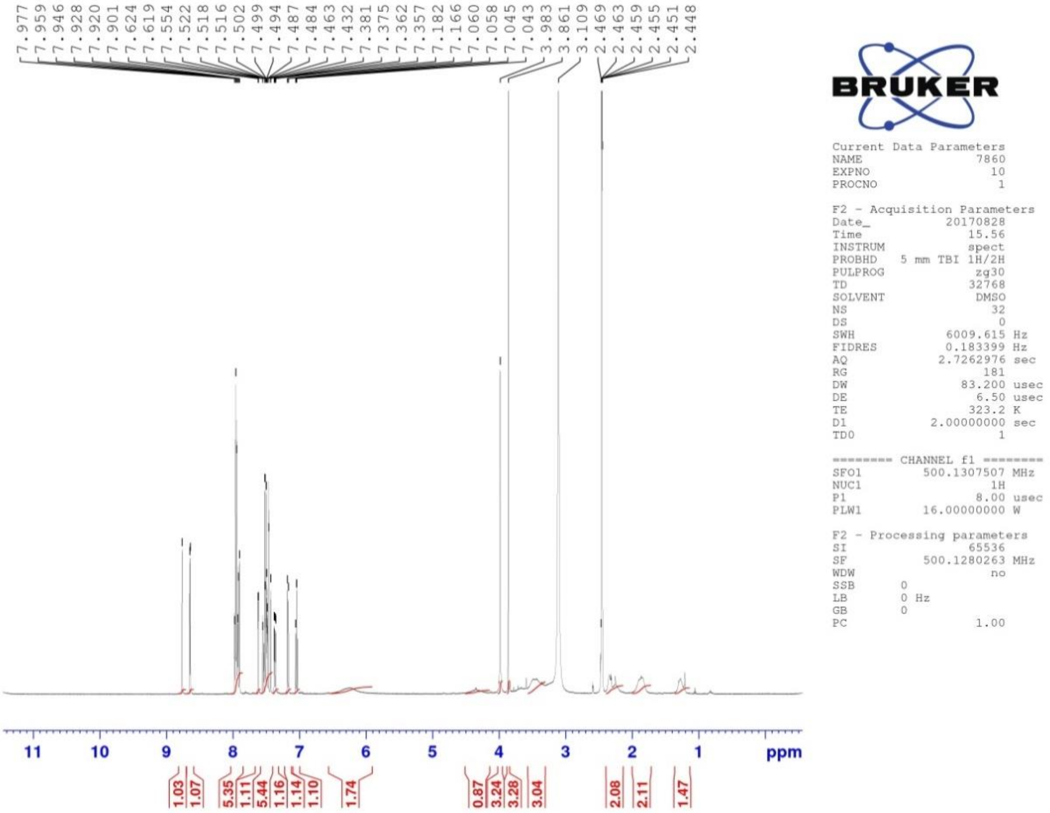

^{13}C-NMR spectra of **10a**

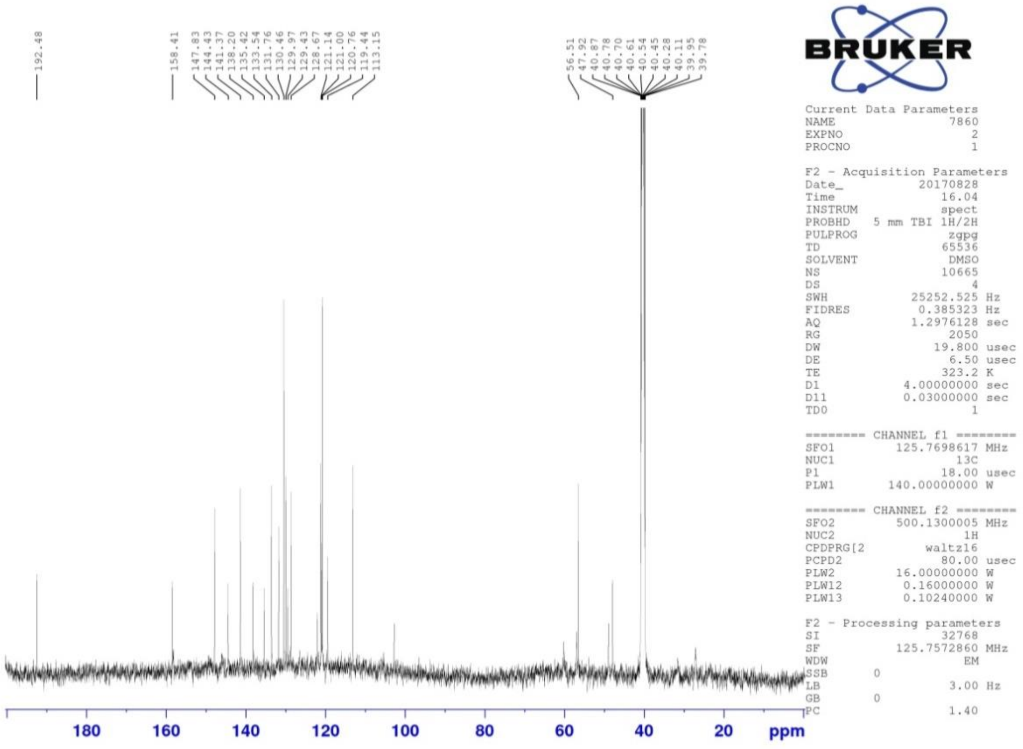

^{1}H-NMR spectra of **10b**

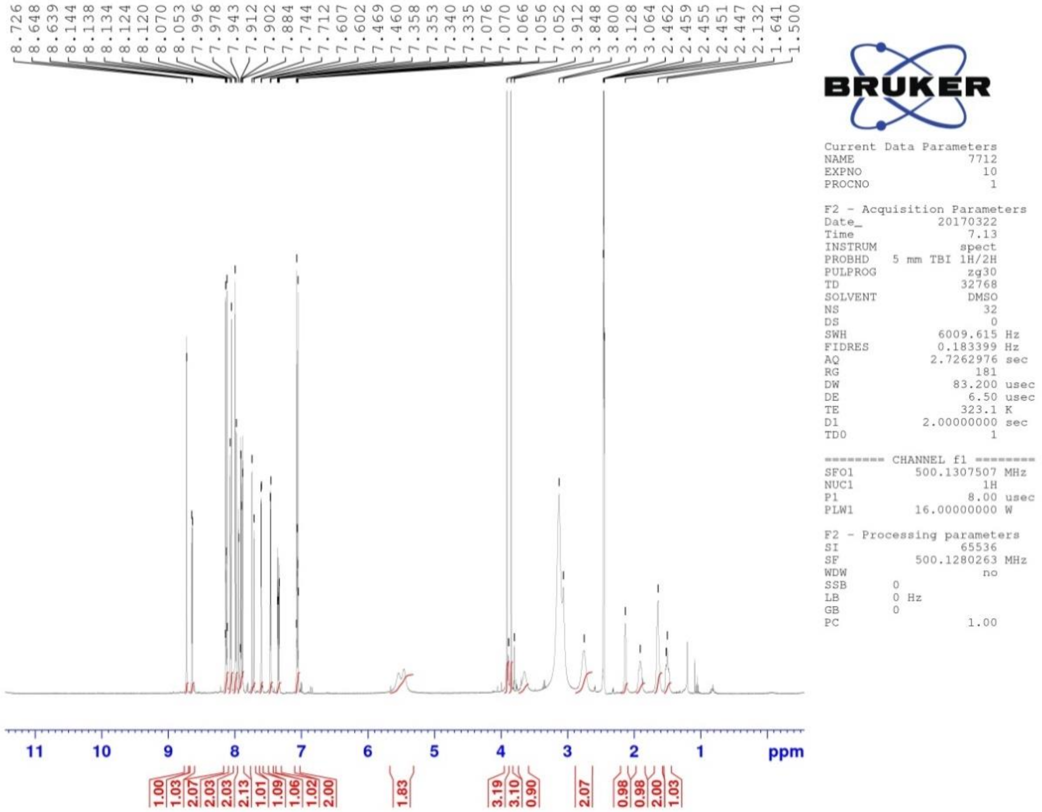

^{13}C-NMR spectra of **10b**

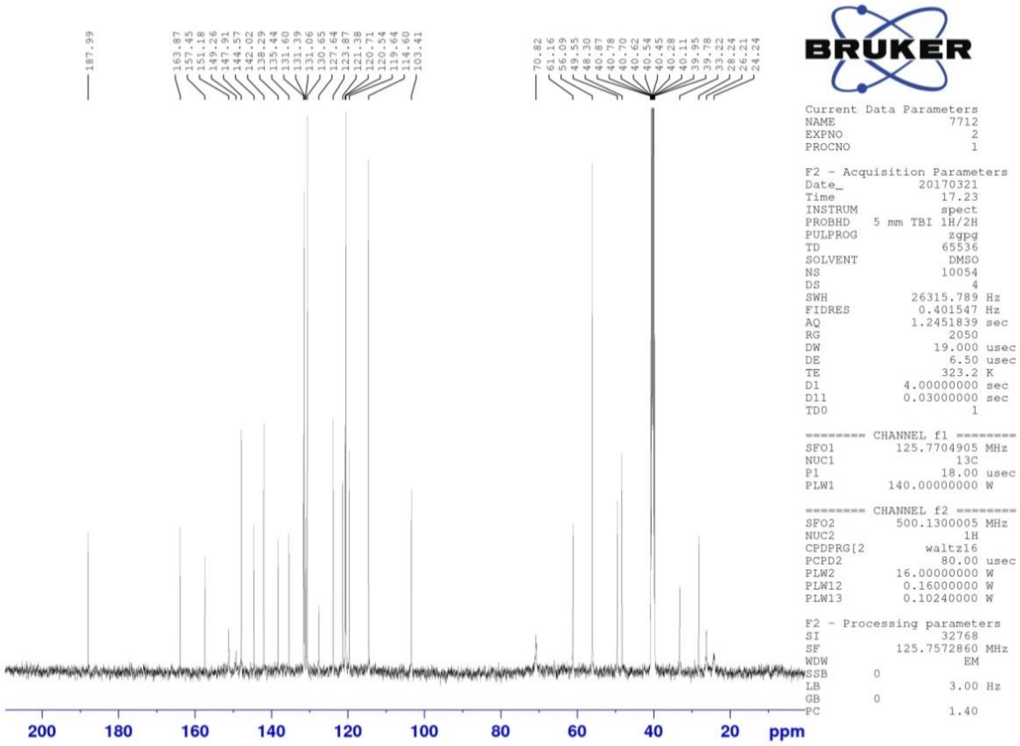

^{1}H-NMR spectra of **10c**

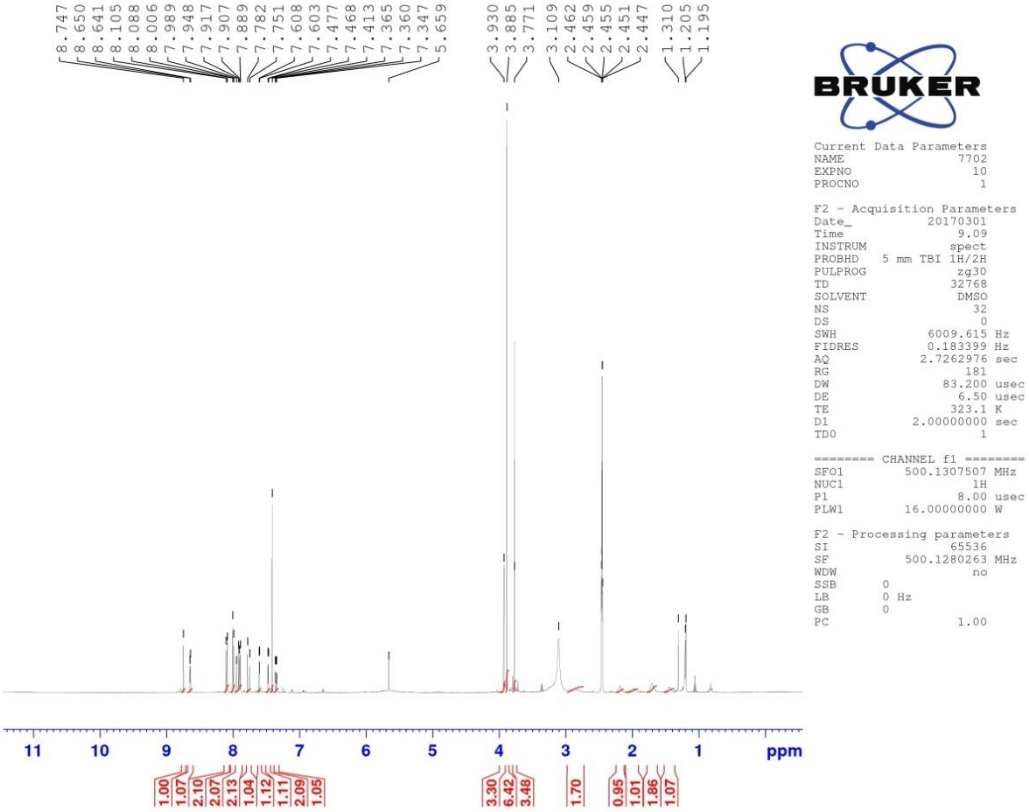

^{13}C-NMR spectra of **10c**

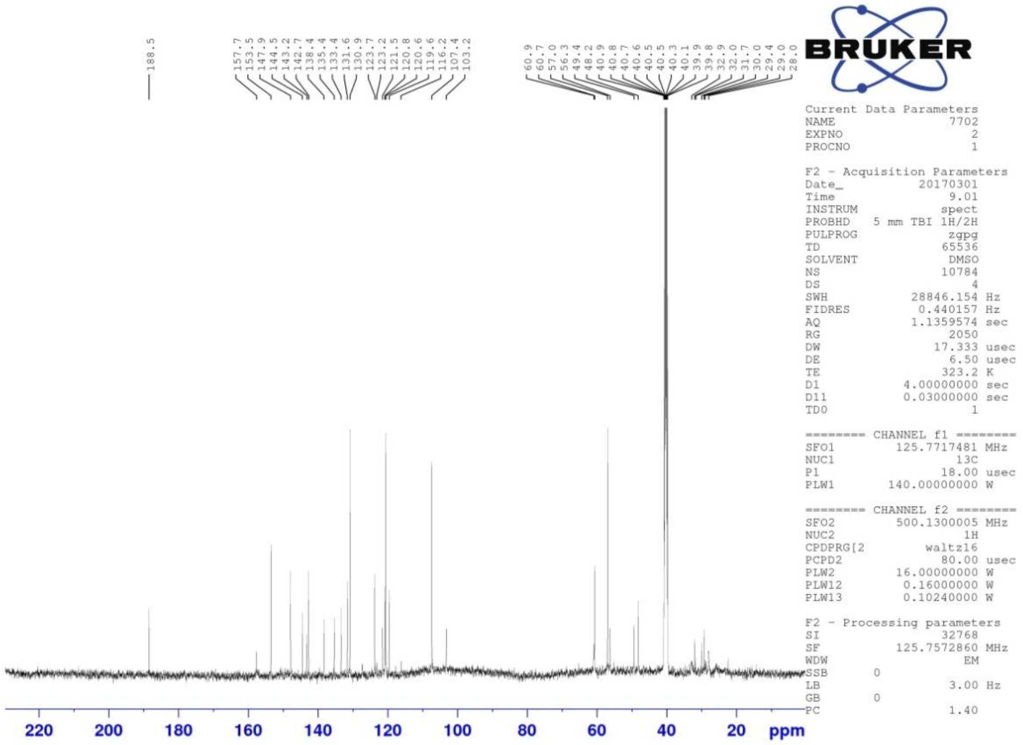

^{1}H-NMR spectra of **10d**

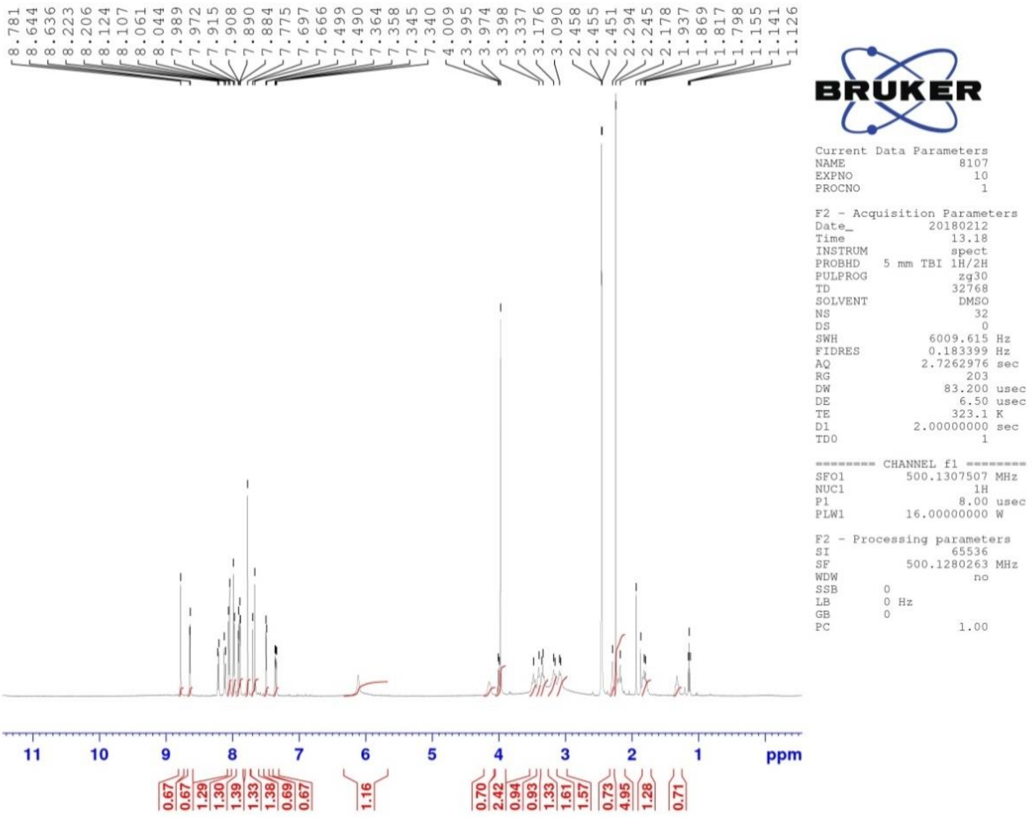

^{13}C-NMR spectra of **10d**

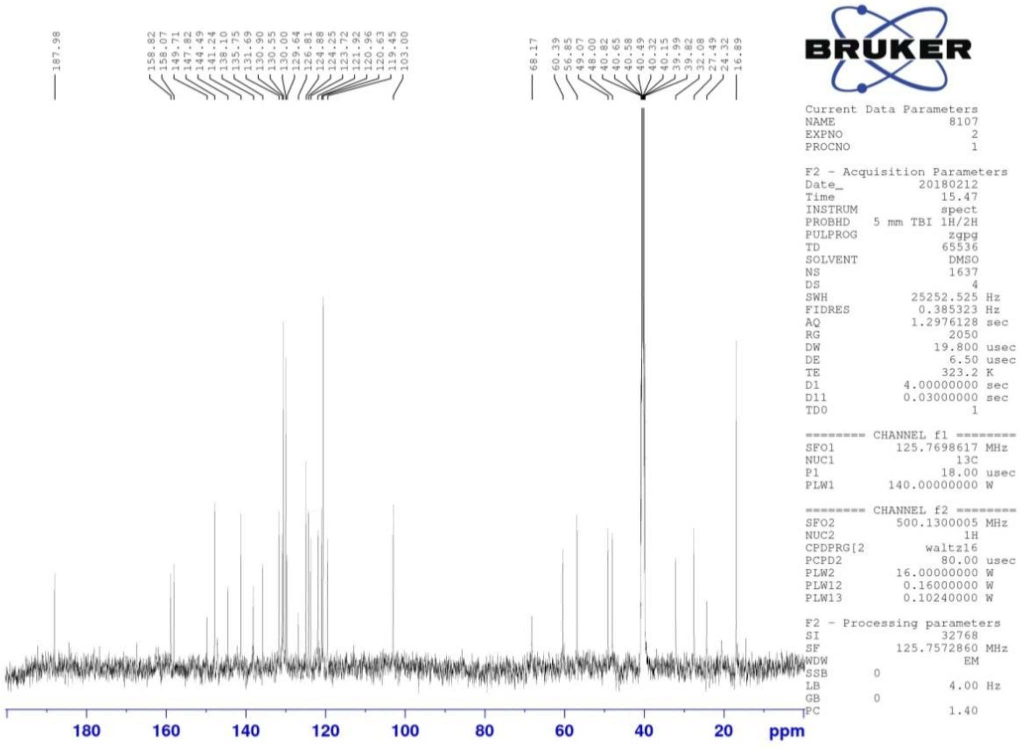

^{1}H-NMR spectra of **11c**

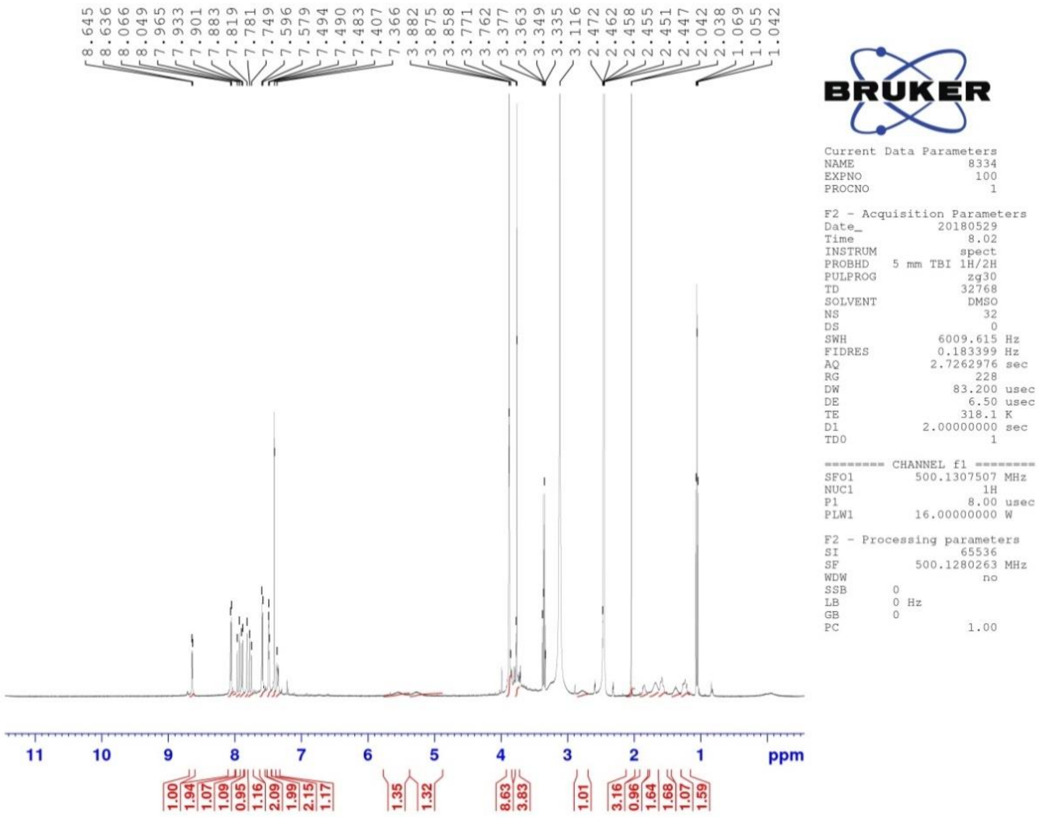

^{13}C-NMR spectra of **11c**

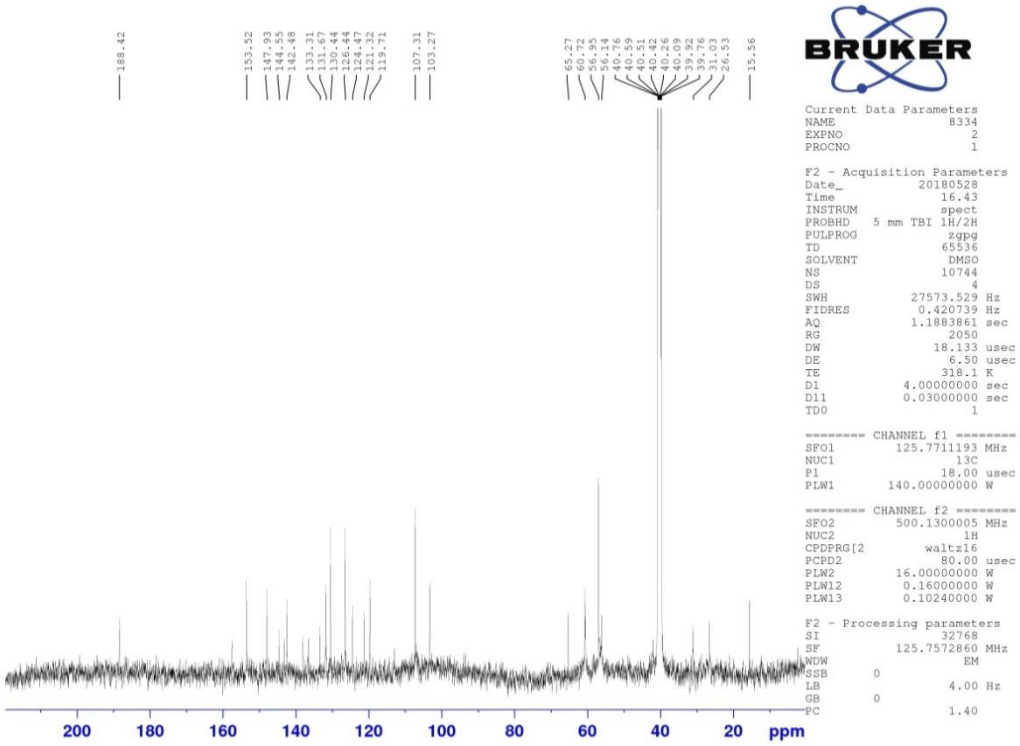

^{1}H-NMR spectra of **11d**

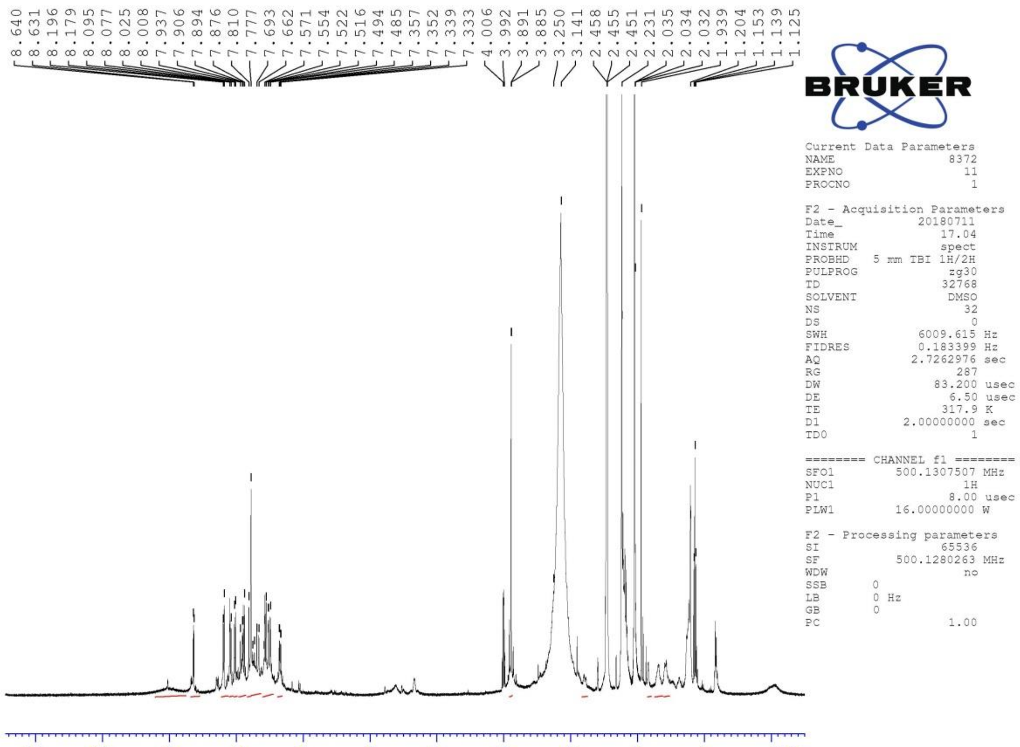

^{1}H-^{13}C-HSQC spectra of **11d**

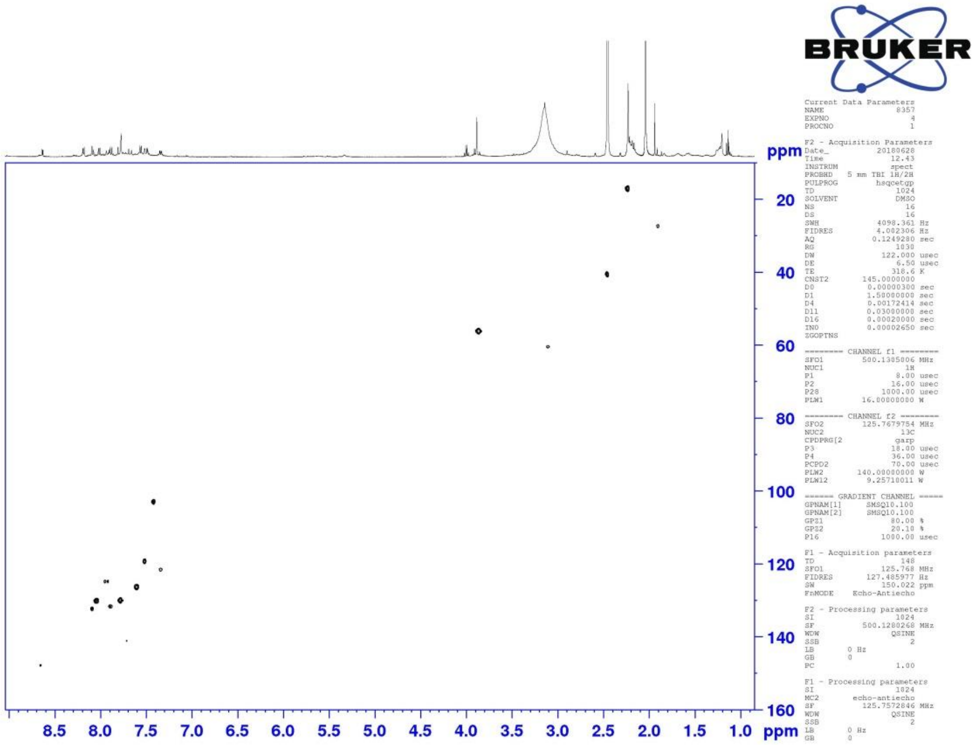

^{1}H-^{13}C-HMBC spectra of **11d**

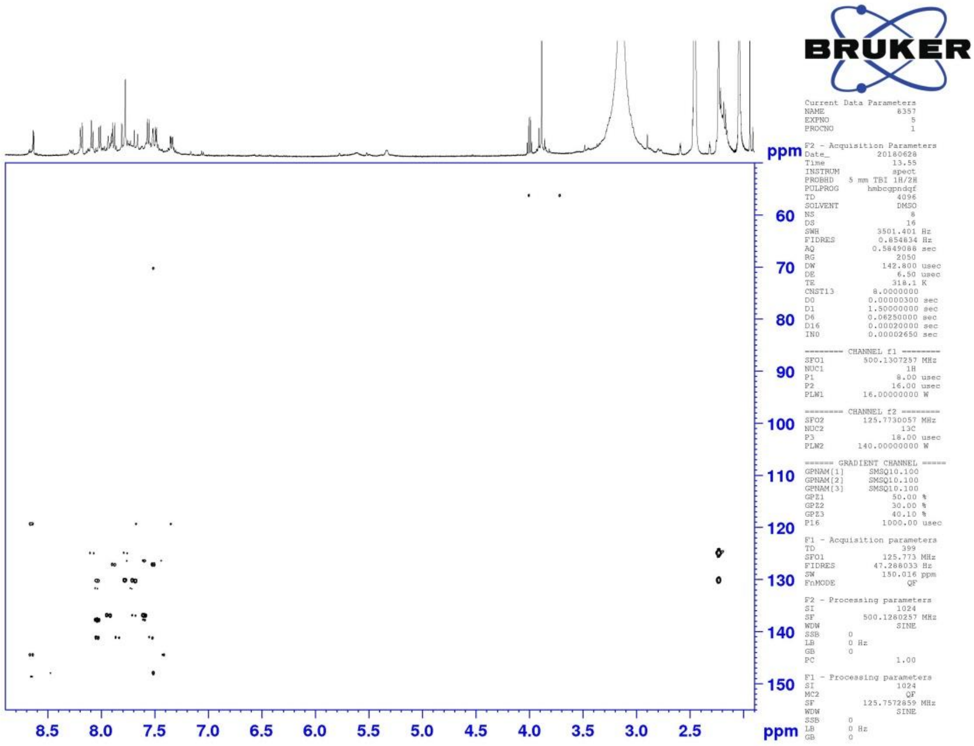

^{1}H-NMR spectra of **12c**

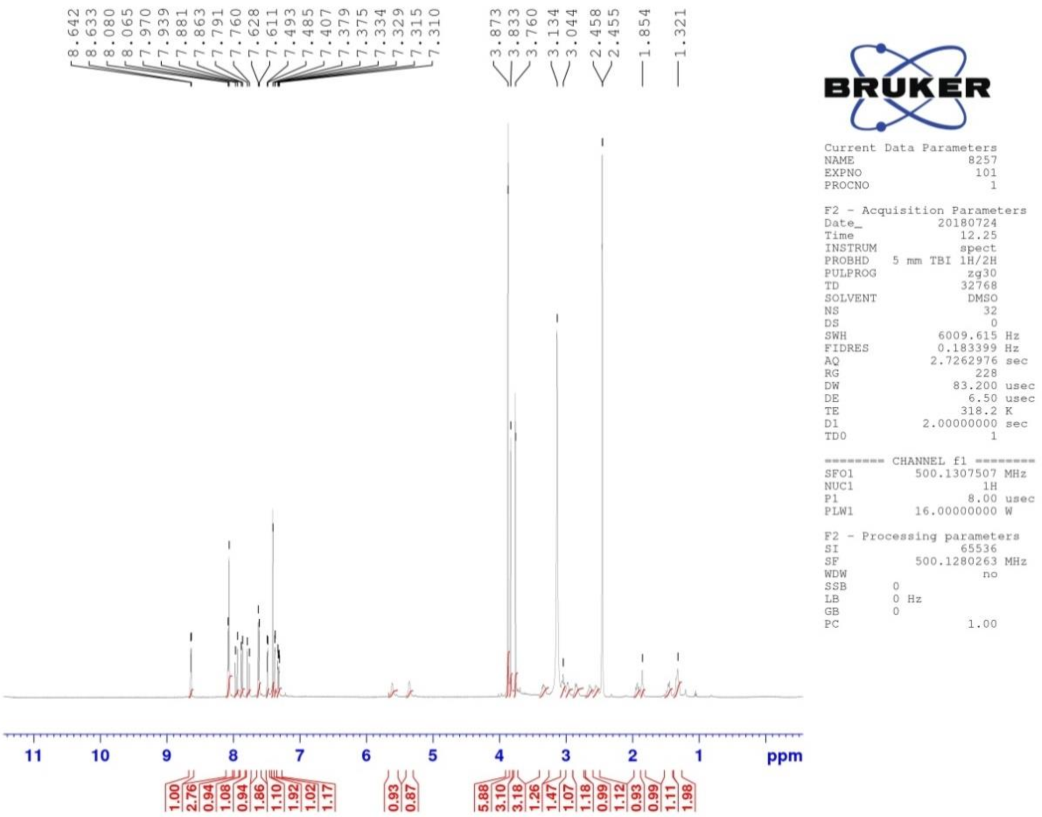

13C-NMR spectra of **12c**

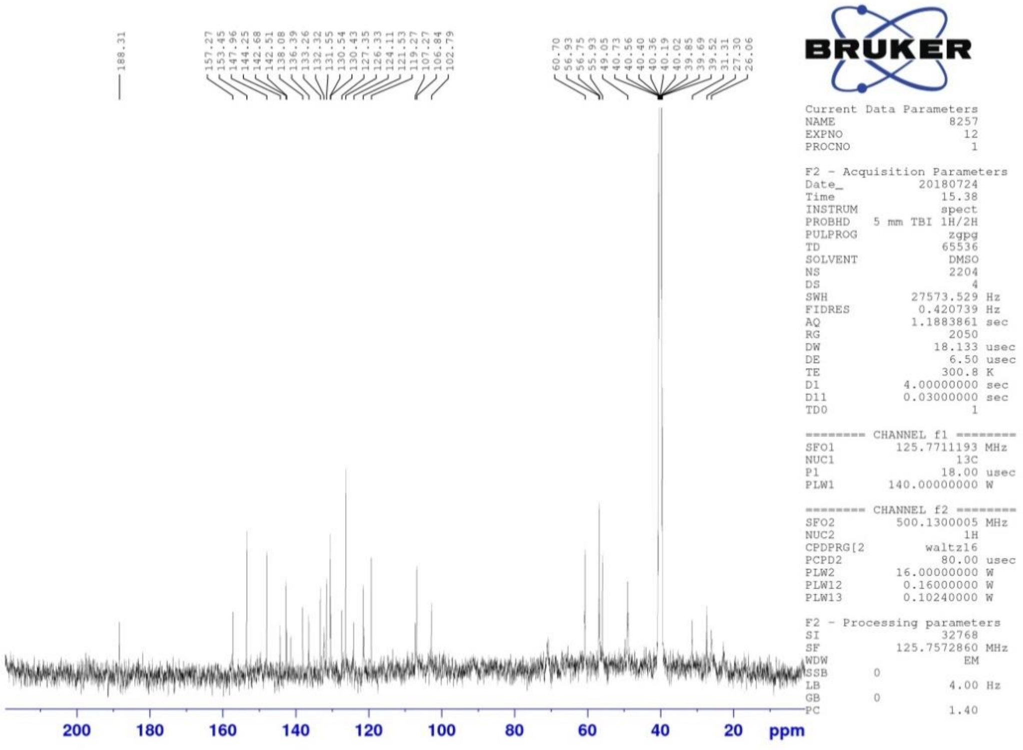

1H-NMR spectra of **12d**

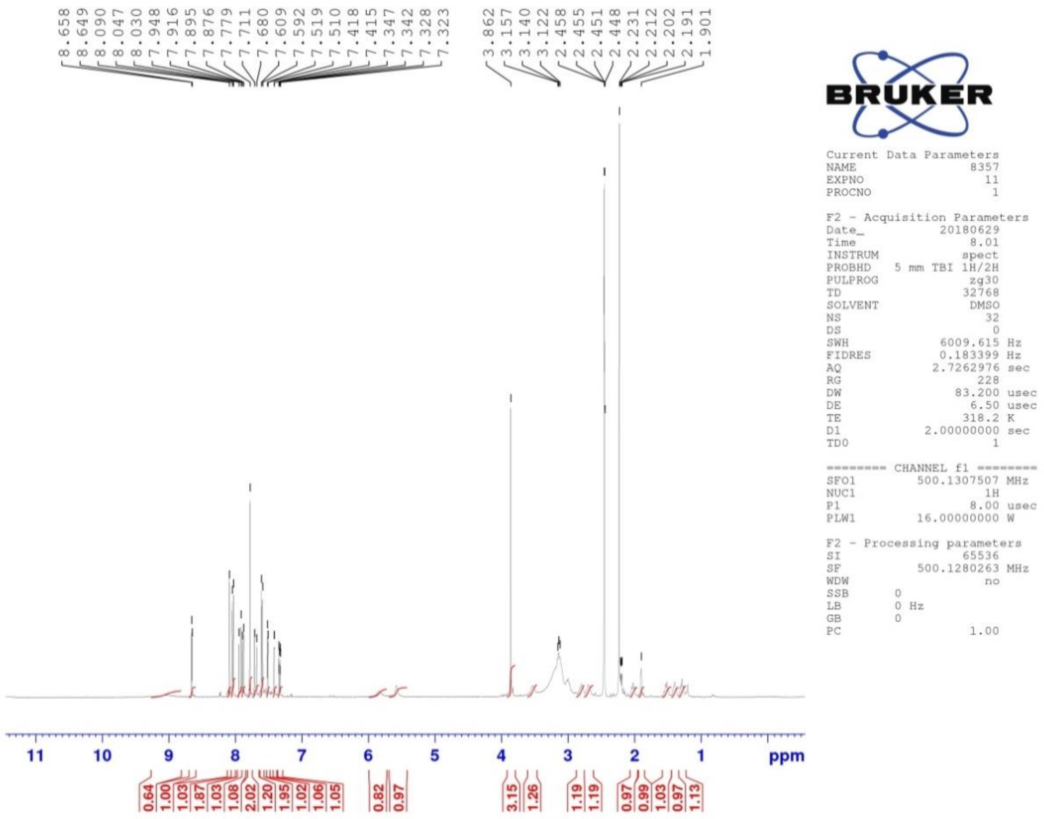

^{13}C-NMR spectra of **12d**

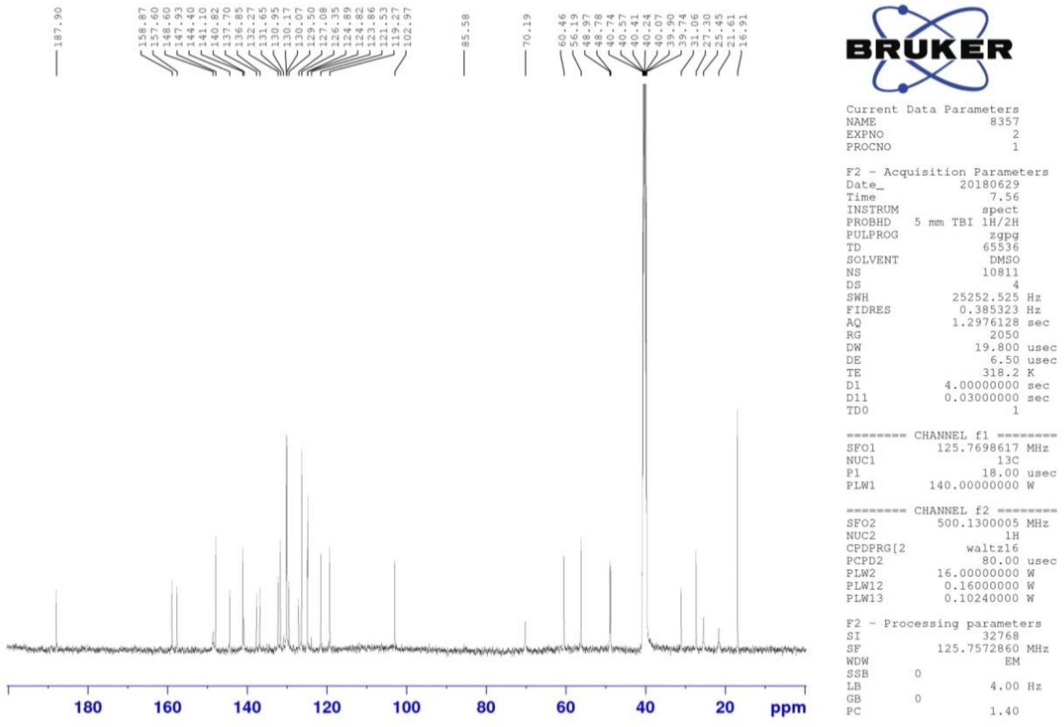

^{1}H-^{13}C HSQC spectra of **12d**

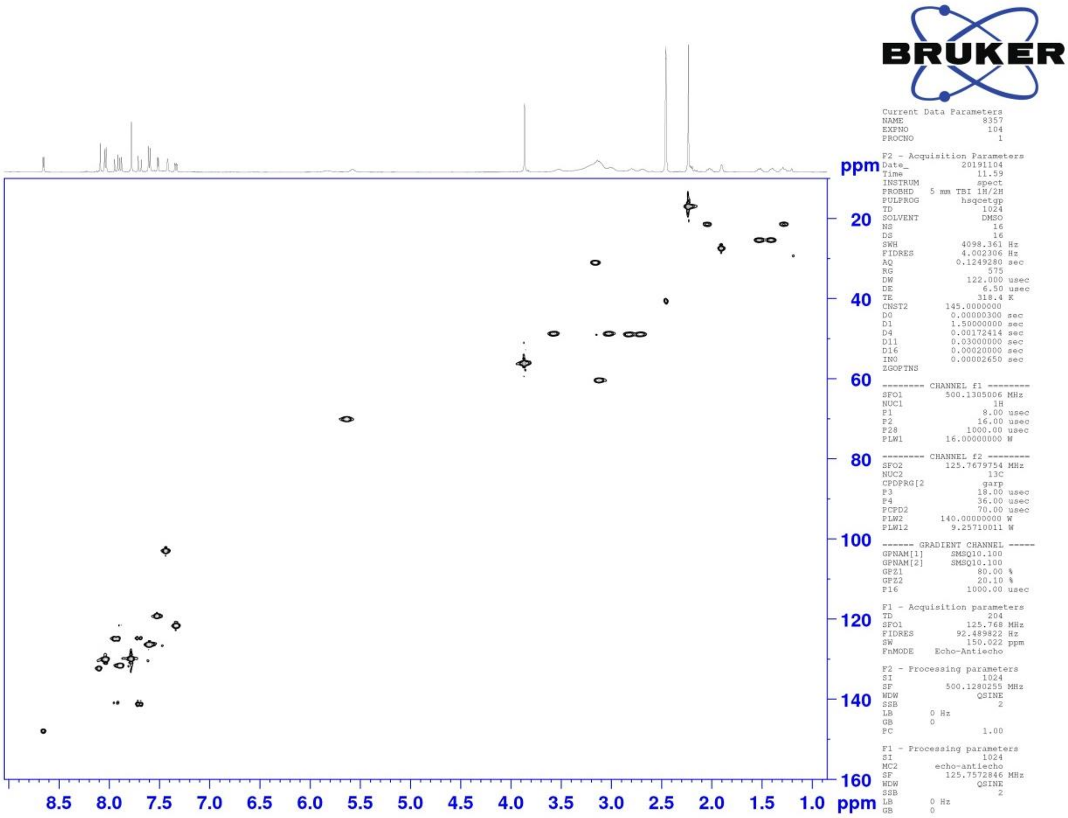

^{1}H-^{13}C HMBC spectra of **12b**

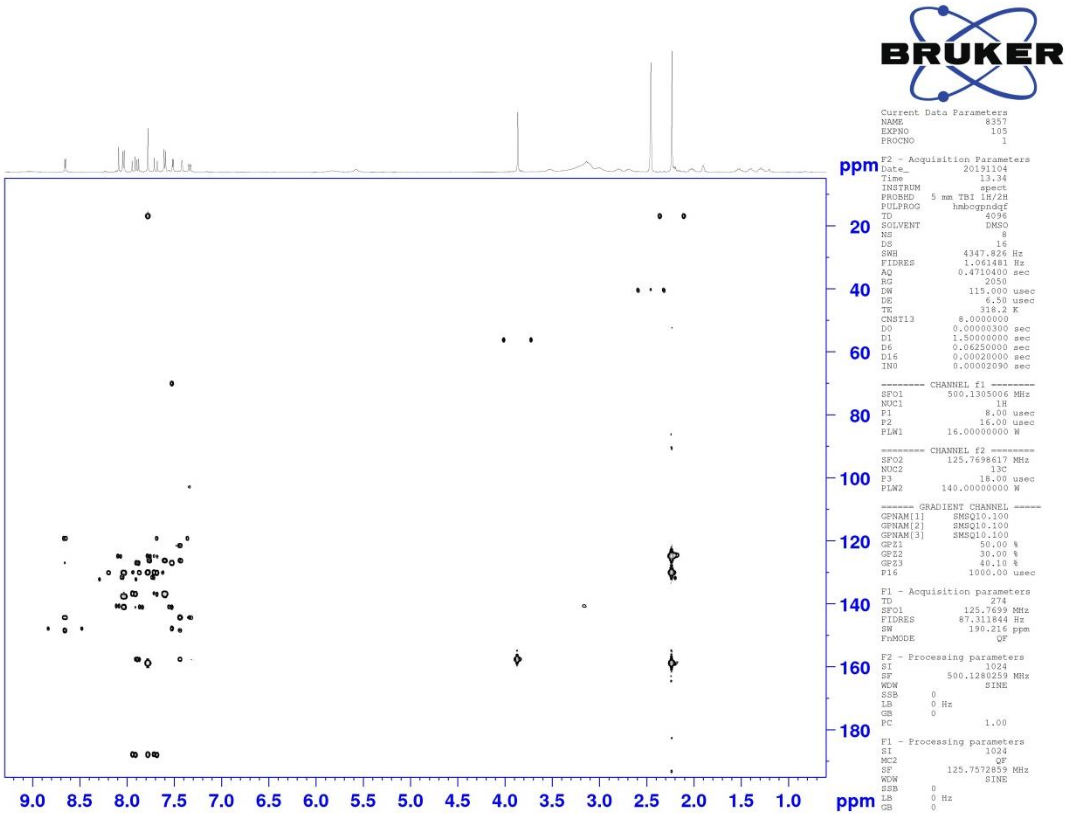

^{1}H-^{1}H NOESY spectra of **12d**

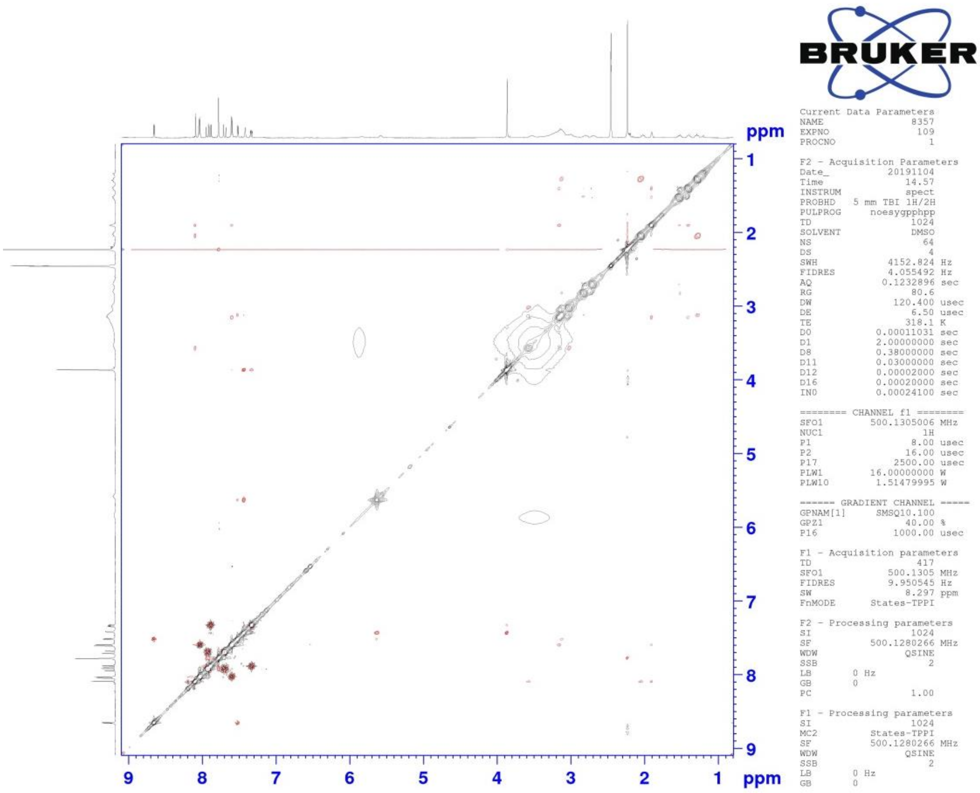

Supplement: Supplementary file 1 [file molecules-24-04077-s001.pdf]
